# Restoring the glioblastoma tumor microenvironment by targeting C5a with the antagonist W54011

**DOI:** 10.1038/s41598-025-30853-1

**Published:** 2025-12-06

**Authors:** Yoojung Oh, Jihwan Yoo, Dongkyu Lee, Bongki Ko, Jun Pyo Hong, Ju Hyung Moon, Eui Hyun Kim, Jong Hee Chang, Yong-Chul Kim, Seok-Gu Kang

**Affiliations:** 1 Department of Neurosurgery, Brain Tumor Center, Severance Hospital, Yonsei University College of Medicine, 50-1 Yonsei-ro, Seodaemun-gu, Seoul, 03722 Republic of Korea; 2 Brain Tumor Translational Research Laboratory, Department of Biomedical Sciences, Yonsei University College of Medicine, Seoul, 03722 Republic of Korea; 3 Brain Research Institute, Yonsei University College of Medicine, Seoul, 03722 Republic of Korea; 4 Department of Neurosurgery, Gangnam Severance Hospital, Yonsei University College of Medicine, Seoul, 06273 Republic of Korea; 5 School of Life Sciences, Gwangju Institute of Science & Technology, 123 Cheomdangwagi-ro, Buk-gu, Gwangju, 61005 Republic of Korea; 6 Department of Neurosurgery, Graduate School of Medical Science, Brain Korea 21 Project, Yonsei University College of Medicine, Seoul, 03722 Republic of Korea

**Keywords:** Complement component 5a, Glioblastoma, Tumor mesenchymal stem-like cell, Tumor microenvironment, Tumorsphere, W54011, Cancer microenvironment, CNS cancer, Cancer stem cells

## Abstract

Glioblastoma (GBM) poses a serious challenge due to its aggressive nature and poor prognosis. Tumor mesenchymal stem-like cells (tMSLCs) secrete complement component 5a (C5a), altering the tumor microenvironment (TME) and promoting tumor progression. This study investigated W54011, a C5a antagonist, to counteract C5a-induced malignancy in GBM tumorspheres. We assessed GBM tissues for C5a receptor 1 (C5aR1) expression using gene profiling and survival analysis. GBM tumorspheres were cultured in C5a-enriched conditioned medium (CM) from tMSLCs to induce tumor stimulation. We evaluated proliferation, invasion, and stemness of GBM tumorspheres using WST/ATP, matrigel invasion assay, and limiting dilution assays. Results were validated via western blotting and RNA sequencing. Additionally, findings were corroborated in an in vivo xenograft mouse model. High C5aR1 expression correlated with increased TME, inflammation-related gene expression, and poorer patient outcomes. CM treatment increased GBM tumorsphere proliferation, invasion, and stemness, which were reversed by W54011. CM also induced the epithelial-mesenchymal transition, whereas W54011 restored spherical morphology and induced apoptosis. In xenograft models, CM-treated GBM tumorspheres led to larger tumors and decreased survival, whereas W54011 decreased tumor size and improved survival. This study suggests a potential role for C5a in GBM progression and supports further investigation of W54011 as a therapeutic candidate.

**Supplementary Information:**

The online version contains supplementary material available at 10.1038/s41598-025-30853-1.

## Introduction

Glioblastoma (GBM) poses a formidable challenge in oncology and is the most prevalent malignant brain tumor among adults, characterized by a grim prognosis despite current treatments available^1^. Standard therapies, including radiotherapy and temozolomide (TMZ) chemotherapy, offer only modest survival benefits^2–4^. Even with advances in cancer therapeutics, such as immunotherapy and targeted approaches, outcomes for GBM remain unfavorable^5^. The tumor microenvironment (TME), which is composed of the extracellular matrix (ECM), interstitial fluid, and various stromal cells, plays a critical role in driving tumor progression and resistance to treatment^6^. Notably, the GBM TME promotes tumor growth, invasion, and the epithelial-mesenchymal transition (EMT), further complicating therapeutic interventions^7,8^. Consequently, innovative strategies that specifically target the GBM TME are urgently needed to improve patient outcomes.

Tumor mesenchymal stem-like cells (tMSLCs) and ventricular-derived mesenchymal stem-like cells (vMSLCs) play critical roles in driving the invasiveness and aggressiveness of GBM tumorspheres^9–11^. Our previous studies have shown that isolating tMSLCs correlates with poor patient prognosis^12,13^, as observed in patient cohorts from The Cancer Genome Atlas (TCGA)^14^. Upon exposure to cancer cell-conditioned medium (CM), tMSLCs differentiate into cancer-associated fibroblasts through a TGFβ1-mediated mechanism, promoting cancer cell stemness, the EMT, and invasion^15^. Additionally, tMSLCs remodel the ECM through the CD40/NF-κB/LOX pathway^16^ and contribute to a hyaluronic acid (HA)-rich TME through autocrine complement 5a (C5a) signaling, which increases the invasiveness of GBM tumorspheres^17^. Glioma-associated human mesenchymal stem cells (MSCs) release exosomes containing miR-1587, which promote tumor progression by increasing the proliferation and clonogenicity of glioma stem-like cells (GSCs), thereby increasing tumor mass and reducing host survival^18^. In the context of vMSLCs, they accelerate GBM tumorsphere invasiveness by secreting cytokines that enhance tumor invasion^11,19,20^. These findings collectively highlight the significant role of mesenchymal stem-like cells in driving the aggressiveness and invasiveness of GBM tumorspheres.

C5a plays a pivotal role in tMSLCs by enhancing HA content in the TME, thereby increasing cancer cell invasiveness. The tMSLCs secrete C5a, which activates ERK/MAPK to induce HA synthase-2, leading to elevated HA levels and promoting cancer cell invasiveness^10^. Additionally, C5a upregulates RHAMM (receptor for HA-mediated motility), amplifying intracellular signaling that drives GBM cell invasiveness^21^. This intricate interaction between C5a, tMSLCs, and an HA-rich microenvironment underscores the critical role of C5a in modulating the TME and advancing cancer progression. Moreover, C5a promotes the EMT^10^ and PD-1/PD-L1 expression^22^, and it is involved in regulating MSCs within the immune response and TME, contributing to a pro-invasive TME^23^. It also facilitates MSC proliferation, recruitment, and migration towards tumor sites, enhancing tumor aggressiveness through interactions with the C5a receptor (C5aR)^24,25^.

Based on these cases and previous studies, we hypothesized that C5a secretion by tMSLCs may contribute to GBM malignancy. In this study, we examined the correlation between C5aR expression in GBM tumor tissues and patient prognosis, and explored associated pathways through transcriptome profiling. We further investigated the functional effect of C5a on tumorsphere proliferation, stemness, and invasiveness by modulating C5a levels or inhibiting its activity with W54011, and assessed the therapeutic potential of C5a inhibition in vivo. These results suggest a potential role for C5a in GBM progression and support further investigation of C5a-targeted strategies as a therapeutic approach.

## Results

### RNA expression in patients with glioblastoma: analysis from the Severance cohort

**Fig. 1:**
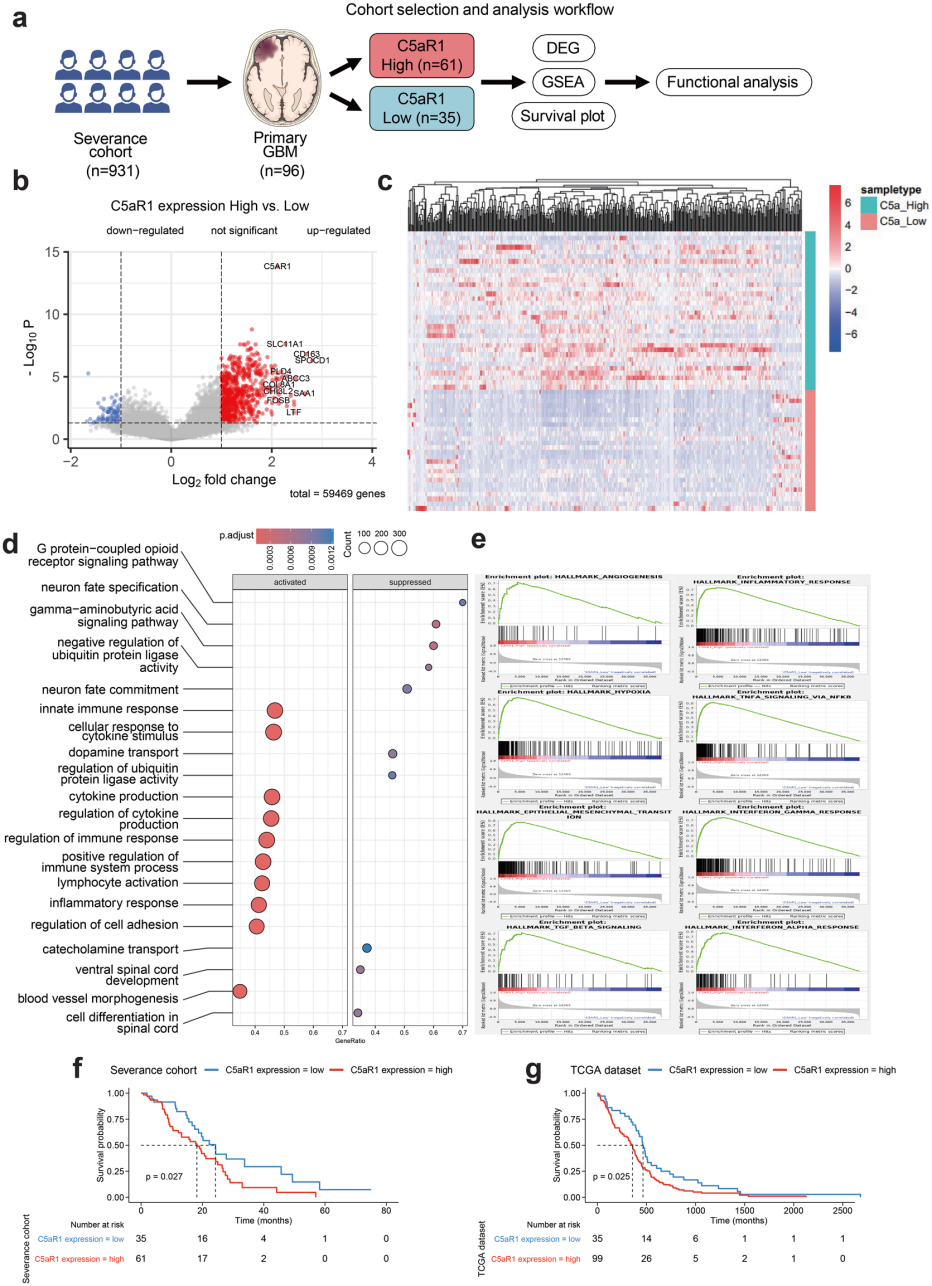
Prognostic potential of C5a as a clinical marker. (**a**) Schematic illustration depicting the informatics analysis conducted on primary glioblastoma (GBM) samples obtained from the Severance cohort. (**b**) Volcano plot showing differentially expressed genes (DEGs) in the C5a High group compared to those in the C5a Low group. Upregulated, downregulated, and insignificantly altered DEGs are indicated. Genes highlighted in white boxes are significantly associated with the tumor microenvironment (TME) and inflammation. (**c**) Heatmap illustrating the expression profiles of statistically significant DEGs (P-adj < 0.05) between the C5a High and C5a Low groups. (**d**) Gene Ontology enrichment analysis for biological processes related to DEGs in the C5a High group versus the C5a Low group. (**e**) Gene set enrichment analysis (GSEA) plot showing the enrichment of hallmark TME and inflammation-related gene sets in the C5a High group relative to the C5a Low group. (**f**) Kaplan–Meier survival curve comparing overall survival between patients with GBM in the C5a High group (n = 61) and those in the C5a Low group (n = 35). Patients in the C5a High group exhibited significantly worse survival outcomes compared to those in the C5a Low group (P = 0.027).

In the Severance cohort, we analyzed 96 patients with GBM, categorizing them into two groups based on C5aR1 expression levels: C5aR1 High (n = 61) and C5aR1 Low (n = 35) (Fig. 1a). Specific clinical information of the 96 samples used in this study are analyzed in Supplementary Table S1. No significant associations were observed between C5aR1 expression and MGMT promoter methylation, sex, age, or 1p/19q codeletion. However, a significant association was identified with TERT mutation status (P = 0.015). These data provide a clinical context for subsequent experiments and support the clinical relevance of C5aR1 in GBM. Our analysis identified a total of 1,246 differentially expressed genes (DEGs). Notably, genes such as *C5aR1*, *IL10*, *TGFBR2*, *STAT6*, *CCL2*, *CXCL8*, *CSF1*, *IL6*, *CCL3*, and *MMP19* were significantly upregulated in the C5aR1 High group (Fig. 1b), revealing distinct gene expression profiles between the two groups (Fig. 1c). Gene Ontology (GO) analysis based on molecular function indicated upregulation in pathways related to the innate immune response, cellular response to cytokine stimulus, cytokine production, and the regulation of cytokine production in the C5aR1 High group (Fig. 1d). Gene Set Enrichment Analysis of 50 hallmark gene sets revealed the elevated expression of genes associated with the EMT, angiogenesis, apoptosis, hypoxia, and inflammatory response in the C5aR1 High group (Fig. 1e). Clinically, patients in the C5aR1 High group exhibited a significantly poorer overall survival (P = 0.027) (Fig. 1f). In the TCGA GBM cohort, external validation reproduced the key findings observed in the Severance cohort. Patients with high C5aR1 expression exhibited significantly worse overall survival compared to those with low expression (log-rank P = 0.025), supporting the prognostic relevance of C5aR1 in GBM. These results validate the robustness and generalizability of our findings across independent datasets (Fig. 1g). To evaluate the relationship between C5aR1 expression and established molecular subtypes, we applied both Verhaak and TME-based classifications to our in-house cohort^26,27^. High C5aR1 expression was significantly enriched in the Mesenchymal subtype and TME-High group, whereas low expression was associated with the Proneural and TME-Low subtypes (Supplementary Table S2). These findings are consistent with previous reports by Verhaak et al. and White et al., which describe Mesenchymal and TME-High tumors as more immunologically active and pro-inflammatory, aligning with the immune-rich microenvironment linked to elevated C5aR1 expression^10,26–28^.

### W54011 exerts a potent inhibitory effect on C5a-driven tumorsphere growth by impeding proliferative processes within the tumor microenvironment

To stimulate C5a upregulation and replicate the TME, we cultured tMSLCs following established protocols and collected the supernatant to obtain C5a-enriched CM. Prior research validated that CM contains numerous cytokine factors, with C5a being predominant, which corroborates the findings of our current study^10^. Consistently, our cytokine array analysis demonstrated that when tMSLC0903-01 was co-cultured with TS15-88, C5a levels predominantly increased, and ImageJ-based quantification confirmed its enrichment relative to other cytokines (Supplementary Fig. S1a–b). We then explored the potential of W54011, a C5aR antagonist, to counteract the disruption of the TME induced by C5a upregulation, which exacerbates tumor progression in GBM tumorspheres. W54011 was synthesized and prepared using a previously published method^28^, and its specific binding and interactions are analyzed in Supplementary Fig. S2 and Supplementary Methods.

As an initial screening step, we performed microarray analysis of GBM tissues and patient-derived tumorspheres. C5aR1 expression was significantly elevated in GBM tissue (n = 58) compared to that in normal tissue (n = 8), whereas C5aR2 levels remained unchanged (Supplementary Fig. S3a). Specifically, C5aR1 expression was markedly elevated in the tumor tissue derived from patient TS15-88, whereas C5aR2 levels showed no significant variation (Supplementary Fig. S3b). Among the tumorspheres, TS15-88 exhibited higher C5aR1 levels than TS14-15 and U87 (Supplementary Fig. S3c). Expression profiling of C5aR1 and C5aR2 in normal and GBM tissues showed that C5aR1 was generally elevated in GBM than in its normal counterparts (Supplementary Fig. S4). Based on these findings, we selected TS15-88 and TS14-15 tumorspheres, both with relatively high C5aR1 expression, along with U87 cells, for downstream analyses. Clinical data of the selected GBM tumorspheres are summarized in Supplementary Table S3.

**Fig. 2:**
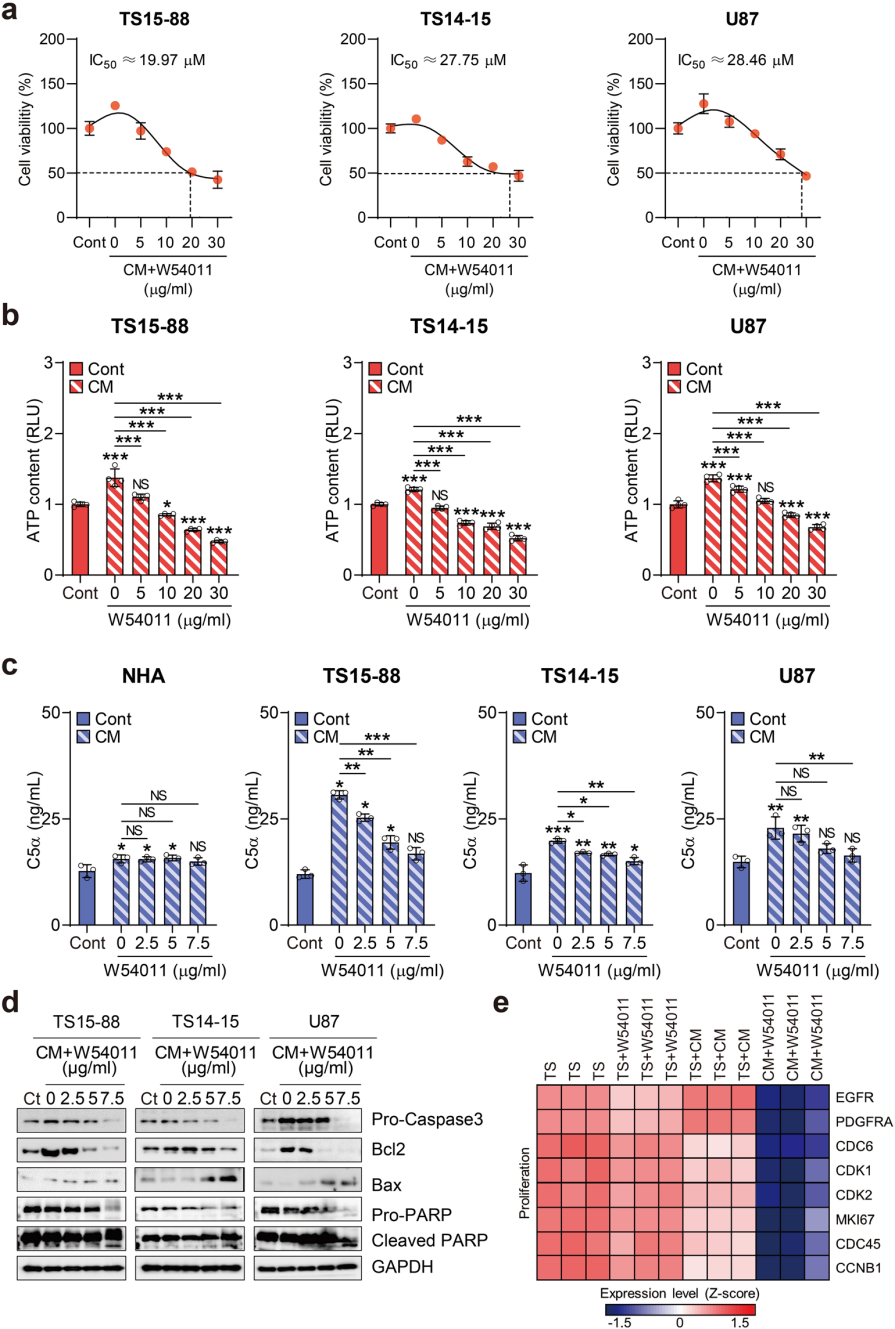
Evaluation of cell viability, ATP levels, and molecular changes in glioblastoma tumorspheres treated with C5a-enriched conditioned media and W54011. (**a**,**b**) Assessment of cell viability and ATP levels in three glioblastoma (GBM) tumorspheres (TS15-88, TS14-15, and U87) treated with C5a-enriched conditioned media (CM) alone or in combination with W54011 at varying concentrations (0–30 μM). Graphs show the dose–response curve used to calculate IC_50_ values (solid bars, control [Cont]; bars with slashed lines, C5a-enriched CM-treated). The “0 μM” W54011 condition refers to treatment with CM in the absence of W54011 (i.e., CM + 0 W54011), which served as a positive control to evaluate the full effect of C5a without receptor inhibition. (**c**) Quantification of C5a levels in CM derived from GBM tumorspheres using ELISA. The cells were treated with C5a-enriched CM alone or in combination with W54011 at concentrations ranging from 0–7.5 μM (solid bar, Cont; bar with slashed lines, C5a-enriched CM-treated). (**d**) Western blotting analysis of the three GBM tumorspheres treated with C5a-enriched CM alone or in combination with W54011 at indicated concentrations (0, 2, 5, and 7.5 μM). Blots were probed with antibodies against pro-caspase-3, Bcl-2, Bax, pro-PARP, cleaved PARP, and GAPDH. (**e**) Heatmap illustrating changes in the expression of genes within the GBM amplification marker gene set between GBM tumorspheres treated with C5a-enriched CM alone or with 7.5 μM W54011. Statistical analysis was performed using one-way analysis of variance, followed by Tukey’s post hoc test. Data are presented as means ± standard deviation, with statistical significance indicated by asterisks (*P < 0.05, **P < 0.01, ***P < 0.001) to denote differences between groups or relative to control conditions; NS, not significant.

Treatment with C5a-enriched CM resulted in increased cell viability in TS15-88, TS14-15, and U87 cells, as measured via WST-1 and ATP assays. However, this elevated cellular viability was gradually diminished after the administration of W54011 at concentrations ranging from 0 to 30 µM (Fig. 2a,b). To determine whether W54011 specifically targets high C5a levels, we quantified the C5a concentration in CM using ELISA. This analysis revealed a significant increase in C5a levels compared to those in the control medium (Fig. 2c). Maximum C5a release from CM was observed at 15.57 ng/mL for normal human astrocytes (NHAs), 30.64 ng/mL for TS15-88, 19.83 ng/mL for TS14-15, and 22.86 ng/mL for U87. While CM exposure led to heightened C5a release from NHAs, W54011 treatment did not affect C5a levels. In contrast, W54011 treatment significantly reduced C5a levels in TS15-88, TS14-15, and U87. Additionally, TS15-88 and TS14-15 exhibited higher C5a release compared to that by NHAs. This quantitative assessment suggests the potential of W54011 to reduce C5a levels in GBM tumorspheres. Supplementary assays in TS15-88 without CM further demonstrated that W54011 alone had minimal impact on proliferation, invasion, and neurosphere formation up to 5 µg/mL, with only a slight reduction observed at 7.5 µg/mL, indicating limited intrinsic toxicity (Supplementary Fig. S5a–c). Together, these results indicate that the inhibitory effects of W54011 are primarily attributable to the blockade of C5a signaling, rather than to nonspecific cytotoxicity, and underscore its potential efficacy in targeting C5a-driven GBM stem-like phenotypes.

We observed that cell viability decreased at a concentration of 2.5 μM W54011, with a more pronounced reduction observed at 7.5 μM. Consequently, we treated cells with 7.5 μM W54011 and conducted western blotting analysis. This analysis revealed that CM treatment increased the sensitivity of cells to survival signals, such as pro-caspase-3 and Bcl-2, which were significantly reduced following W54011 administration (Fig. 2d). Conversely, W54011 treatment increased the levels of the pro-apoptotic molecule Bax and the cleaved form of PARP. RNA sequencing further showed that several proliferation markers were significantly downregulated in GBM tumorspheres treated with W54011 after CM exposure (Fig. 2e). Notably, while CM alone (TS + CM condition) did not strongly enhance tumorsphere proliferation, this is consistent with previous studies suggesting that C5a–C5aR1 signaling primarily contributes to GBM invasiveness and survival, rather than directly promoting cell proliferation. Prior reports have shown that mesenchymal stem-like cells modulate the TME through the C5a/p38/ZEB1 axis to enhance invasion, without significantly affecting proliferation^10,29^. Thus, the relatively modest effect of CM on proliferation likely reflects the biologically specialized role of C5a signaling in GBM pathophysiology, which is more prominently linked to invasiveness and survival signaling than it is to proliferation.

To ensure that these effects were specifically attributable to C5a, we first confirmed that the CM produced by tMSLCs is a major source of C5a. Silencing C5 in tMSLCs (thereby preventing C5a generation) abolished the ability of CM to enhance the proliferation, invasion, and stem cell frequency of TS15-88, as determined through the extreme limiting dilution assay (ELDA), compared with CM from scrambled siRNA controls (Supplementary Fig. S6a–c). Moreover, stem cell frequency varied across TS15-88, with detailed confidence intervals (CIs) provided in Supplementary Table S5. These results directly demonstrate that the tumor-promoting effects of tMSLC-CM are critically dependent on C5a.

### W54011 effectively suppresses C5a-induced glioblastoma tumorsphere stemness and the epithelial-mesenchymal transition through disruption of the tumor microenvironment

In GBM tumorspheres, stemness refers to the heightened stem cell-like properties of these cells, as opposed to those of bulk tumor cells. Factors in the TME, such as growth factors and cytokines, help sustain stemness in GSCs, whereas hypoxia further enhances their self-renewal and pluripotency^30,31^. Based on this, we hypothesized that elevated C5a levels would increase stemness in GBM tumorspheres. To test this hypothesis, we used ELDA to quantify the frequency of stemness in untreated GBM tumorspheres and those treated with either CM or W54011.

**Fig. 3:**
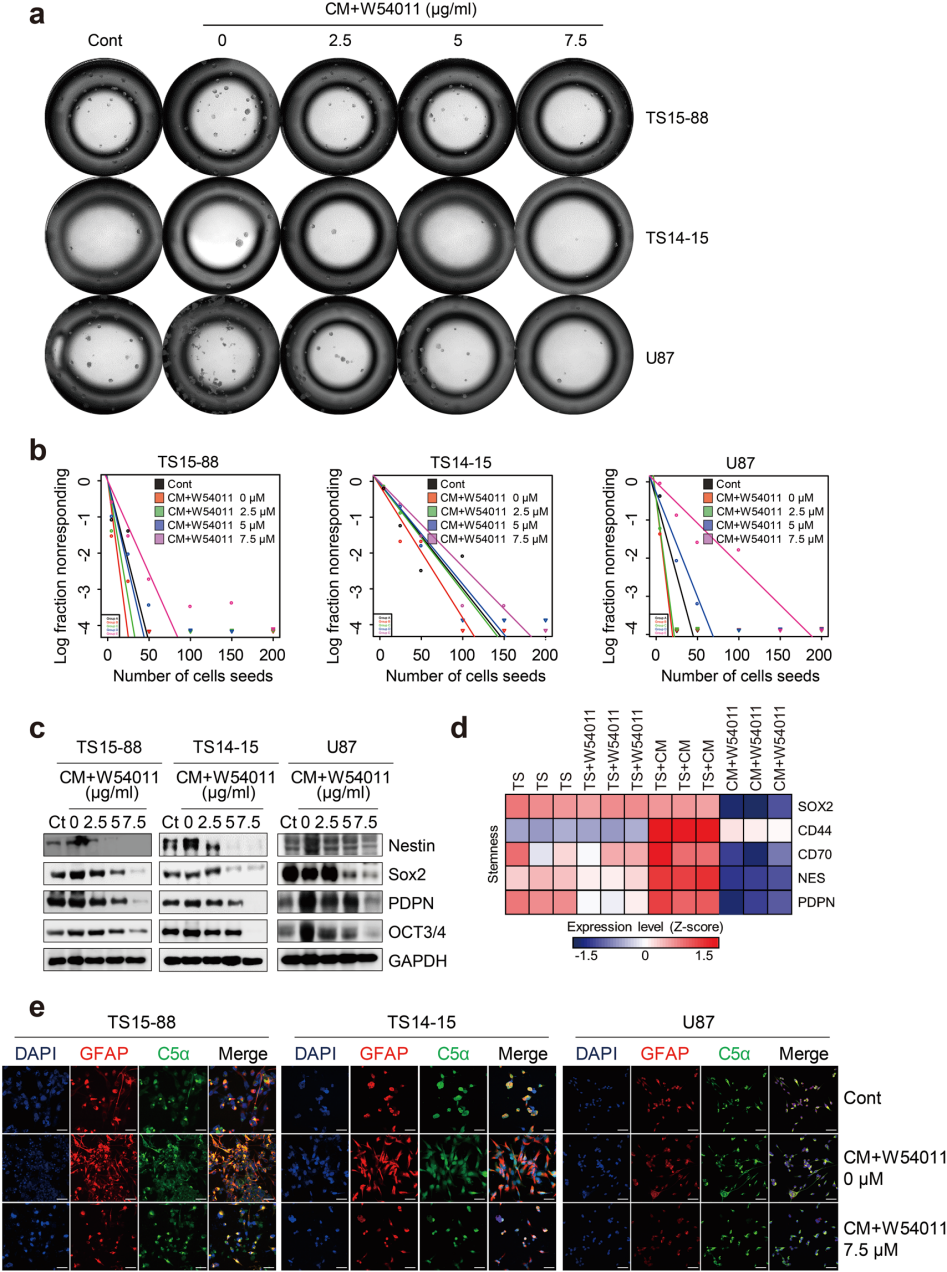
Reduction in stemness and morphological changes in glioblastoma tumorspheres following W54011 treatment in the presence of C5a-enriched conditioned media. (**a**) High-throughput brightfield micrographs of the three glioblastoma (GBM) tumorspheres (TS15-88, TS14-15, and U87) treated with C5a-enriched conditioned media (CM) alone or in combination with W54011 at various concentrations (0–7.5 μM), starting from an initial seeding density of 200 cells. The 0 μM W54011 group represents CM-treated cells without antagonist exposure. (**b**) Frequency of stem-like cells within populations of the three GBM tumorspheres treated with C5a-enriched CM alone or in combination with W54011 at various concentrations (0–7.5 μM), as measured using the extreme limiting dilution assay at 14 days post-seeding. (**c**) Western blotting analysis of the three GBM tumorspheres treated with C5a-enriched CM alone or in combination with W54011 at concentrations of 0, 2, 5, and 7.5 μM. Blots were probed with antibodies against Nestin, Sox2, PDPN, OCT3/4, and GAPDH. (**d**) Heatmap showing changes in gene expression within the GBM stemness marker gene set in GBM tumorspheres treated with C5a-enriched CM alone or in combination with W54011 at 7.5 μM. (**e**) Representative confocal micrographs demonstrating colocalization of GFAP (red) and C5a (green) in GBM tumorspheres treated with C5a-enriched CM alone or in combination with 7.5 μM W54011. Scale bars = 50 μm. Cont, control.

Three GBM tumorspheres (TS15-88, TS14-15, and U87) were freshly dissociated and treated with either CM or W54011, and their self-renewal capacities quantitatively assessed over a period of 14 days. High-throughput imaging of the GBM tumorspheres revealed increased sphere formation with CM treatment and decreased sphere formation with W54011 treatment. Sphere formation was notably reduced with W54011 treatment at concentrations ranging from 2.5 to 7.5 µM (Fig. 3a). The corresponding quantification of ELDA assay data is provided in Supplementary Fig. S7a. Additionally, we performed a quantitative analysis of the CIs for 1/stem cell frequency following CM and W54011 treatments using the ELDA analysis program (http://bioinf.wehi.edu.au/software/elda/), and the results graphically presented (Fig. 3b). ELDA analysis revealed that all three GBM tumorsphere lines showed the highest self-renewal capacity in the CM treatment group. CIs for the reciprocal stem cell frequencies were 5.76 for TS15-88 (95% CI 4.50–7.20), 21.9 for TS14-15 (95% CI 18.0–26.0), and 4.09 for U87 (95% CI 3.50–4.80). TS15-88 exhibited the highest stem cell frequency, indicating a greater presence of stem cells compared to that of TS14-15 and U87. Conversely, TS14-15 had the lowest frequency, suggesting the presence of fewer stem cells. Higher concentrations of W54011 were associated with reduced self-renewal capacity, and TS14-15 also showed the lowest sphere-forming ability. Detailed CIs for stem cell frequencies in the three GBM tumorsphere lines are provided in Supplementary Table S4. Western blotting analysis was used to further assess the expression of stemness markers, and results showed a significant increase in marker expression in all three GBM tumorsphere lines following CM treatment. In contrast, marker expression significantly decreased with increasing concentrations of W54011 (Fig. 3c). RNA sequencing analysis corroborated the upregulation of stemness markers in the CM-treated group, with significant reductions observed in the CM + W54011-treated groups (Fig. 3d).

Previous studies have established a link between the EMT and stemness of GBM tumorspheres, where the EMT plays a crucial role in promoting stem cell-like properties^32,33^, leading to increased plasticity and metastatic potential^34^. To determine whether the enhanced stemness observed in GBM tumorspheres following CM treatment is driven by changes in the TME and EMT induction, we assessed morphological alterations in GBM tumorspheres upon exposure to C5a. Confocal microscopy was performed with co-staining for GFAP, a recognized marker of GBM stemness, and C5a to evaluate the extent of these changes. Our analysis revealed that all three GBM tumorspheres exhibited increased levels of both GFAP and C5a in the CM-treated groups, accompanied by a shift in sphere morphology toward an EMT-like shape. In contrast, treatment with W54011 mitigated this effect, leading to restoration of the original spherical morphology of the cells (Fig. 3e). Quantification of GFAP^+^ and C5a^+^ cells is provided in Supplementary Fig. S7b. These findings suggest that a C5a-enriched TME may enhance GBM stemness and promote EMT progression.

### C5a-induced stemness and the epithelial-mesenchymal transition in glioblastoma tumorspheres contribute to increased invasion, which can effectively be suppressed by W54011 treatment

**Fig. 4:**
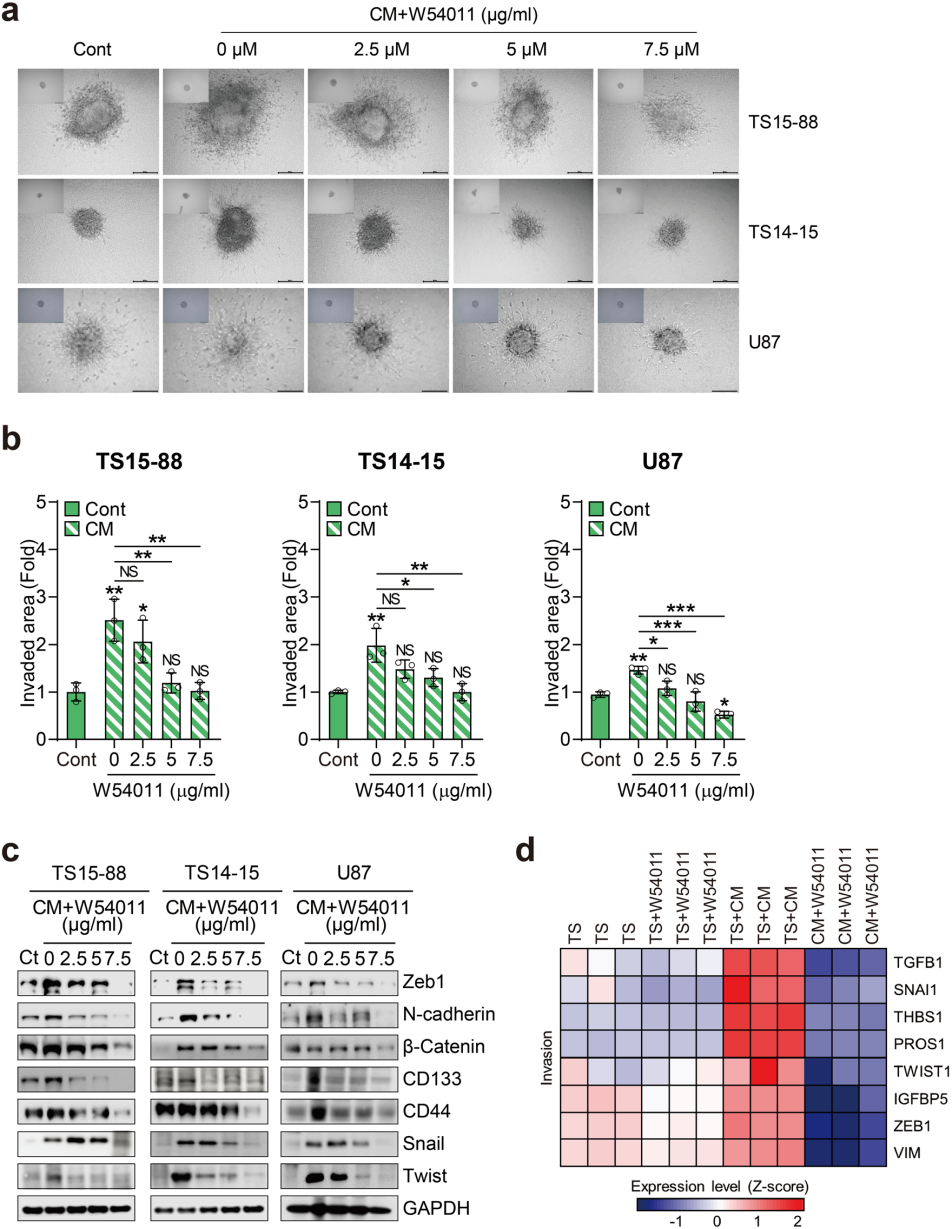
Inhibition of invasion by W54011 in glioblastoma tumorspheres exposed to C5a-enriched conditioned media. (**a**) Time-lapse images of spheroid invasion in three glioblastoma (GBM) tumorspheres (TS15-88, TS14-15, and U87) within a matrix gel. Representative images show spheroids at baseline (0 h, left corner) and after 72 h of treatment with C5a-enriched conditioned media (CM) alone or in combination with W54011 at various concentrations (0–7.5 μM). “0 μM W54011” indicates treatment with C5a-enriched CM alone. Scale bars = 200 µm. (**b**) Quantification of the invasion area of individual spheroids (solid bar, control [Cont]; bar with slashed lines, C5a-enriched CM-treated). (**c**) Western blotting analysis of the three GBM tumorspheres treated with C5a-enriched CM alone or in combination with W54011 at concentrations of 0, 2, 5, and 7.5 μM. Cell lysates were probed with antibodies against Zeb1, N-cadherin, β-catenin, CD133, CD44, Snail, Twist, and GAPDH. (**d**) Heatmap depicting gene expression changes in the GBM invasion marker gene set in GBM tumorspheres treated with C5a-enriched CM alone or with 7.5 μM W54011. Statistical analysis was performed using one-way analysis of variance, followed by Tukey’s post hoc test. Results are presented as means ± standard deviation, with significance indicated by asterisks (*P < 0.05, **P < 0.01, ***P < 0.001), highlighting differences between groups or compared to control conditions.

In Fig. 4, we verified that C5a induces stemness and initiates the EMT, leading to alterations in cell morphology. Our hypothesis posited that C5a-induced EMT would result in a loss of cell–cell adhesion and polarity in GBM tumorspheres, conferring mesenchymal traits and enhancing invasion, as extensively documented in previous studies. To investigate this, we cultured three GBM tumorsphere lines (TS15-88, TS14-15, and U87) for seven days to form spheres, whereafter they were treated with either CM or W54011. These spheres were subsequently implanted into a three-dimensional (3D) matrix, and brightfield imaging performed 72 h after implantation (Fig. 4a). To confirm that the comparative spheroids were tracked, representative 0 h images have been added in the left corner of Fig. 4a. The imaging results revealed that invasion significantly increased in the CM-treated group for all three GBM tumorspheres lines, whereas invasion gradually decreased with varying concentrations of W54011 treatment (2.5–7.5 µM). We quantitatively measured the invasive areas, confirming a marked increase in invasion with CM treatment compared to that in the non-CM-treated group, with progressively lower invasion levels observed at increasing concentrations of W54011 treatment (Fig. 4b). At higher concentrations (notably 7.5 µM), some spheroids—particularly TS15-88 and TS14-15—also exhibited signs of core shrinkage and reduced structural integrity, suggesting possible stress-induced morphological changes. Western blotting analysis further confirmed the elevated expression levels of various invasion markers in the CM-treated group, with levels decreasing upon W54011 treatment (2.5–7.5 µM) (Fig. 4c). Additionally, RNA sequencing results showed increased expression of invasion markers in the CM treatment group, whereas their expression was significantly reduced in the CM and W54011 treatment groups (Fig. 4d). Collectively, these findings indicate that C5a may enhance GBM tumorsphere invasion, which can be effectively counteracted by W54011 treatment. Additionally, to confirm that the CM we had manufactured produces substantial amounts of C5a and targets W54011, we purchased recombinant C5a and processed it with three GBM tumorspheres (TS15-88, TS14-15, and U87). Following treatment with recombinant C5a, we observed an immediate increase in cell proliferation, stemness, and invasion. Importantly, these effects were reversed upon subsequent treatment with W54011 (Supplementary Fig. S8a–f).

### C5a stimulation exacerbates tumor malignancy, whereas W54011 attenuates its tumorigenic impact in brain tumors

**Fig. 5:**
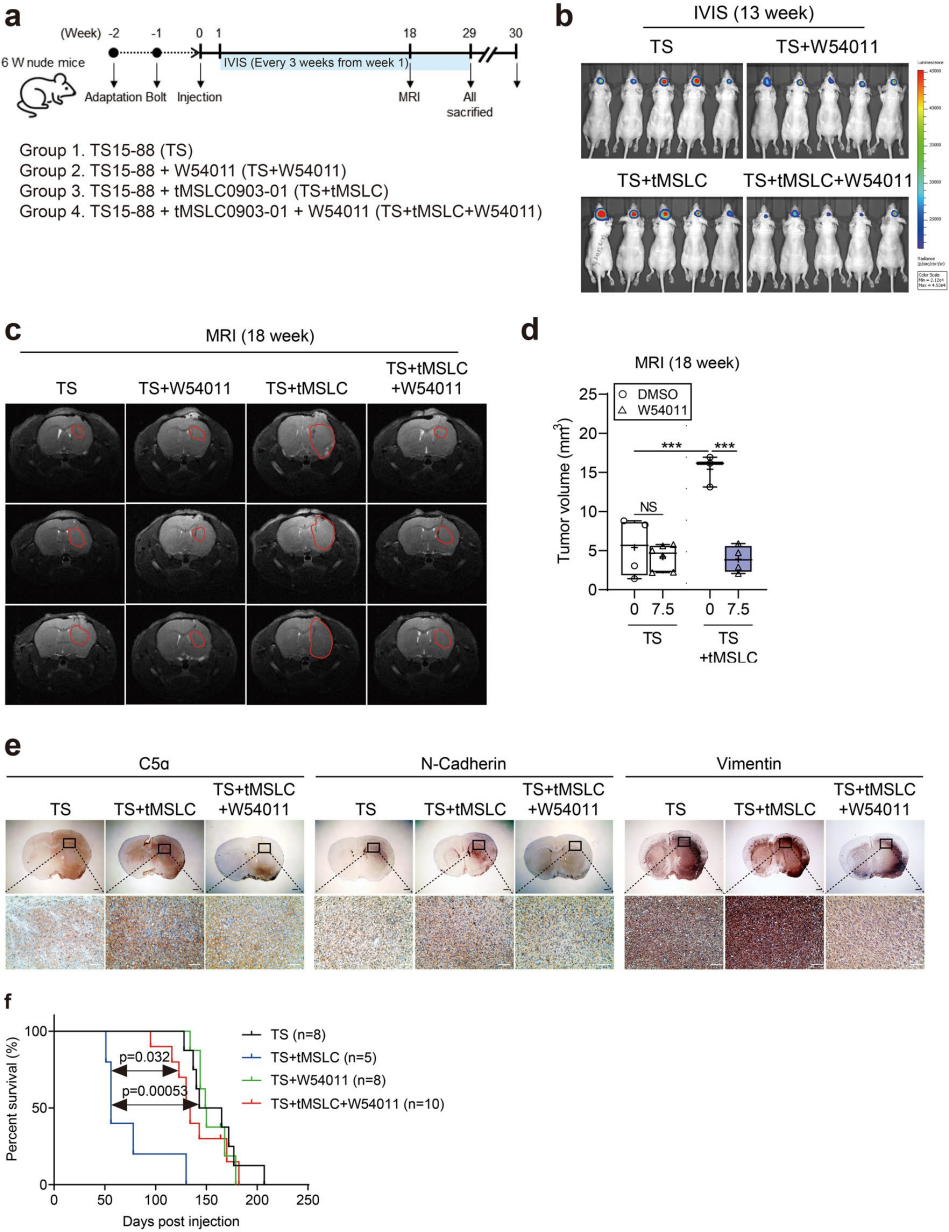
Effect of W54011 on tumor growth in an in vivo model with C5a-enriched conditioned media. (**a**) Schematic illustrating the xenograft model design and treatment protocol. “Adaptation” denotes a one-week acclimation period following animal arrival. “Bolt” indicates surgical implantation of a cranial guide-screw at week –1, enabling orthotopic cell injection at week 0. Eight-week-old BALB/c nude mice were divided into four groups: TS (n = 8), TS + W54011 (n = 5), TS + CM (n = 8), and TS + CM + W54011 (n = 10). Orthotopic xenografts were established with TS15-88-luciferase cells (5 × 10^5^) alone or with TS15-88 (2.5 × 10^5^) and tMSLC0903-01 (2.5 × 10^5^). Mice were treated with or without W54011 before surgical orthotopic implantation to assess the effects of C5a-enriched conditioned media (CM) and W54011 in vivo. (**b**) Tumor growth was monitored using an IVIS Lumina II in vivo imaging system starting on day 8 and every week thereafter. Bioluminescence images show tumor signals obtained at 13 weeks. (**c**) Representative magnetic resonance imaging (MRI) scans at 18 weeks post-inoculation. MRI was performed once at the experimental endpoint to confirm tumor presence and assess volume. Red lines indicate tumor volumes. (**d**) Bar graph quantifying MRI tumor volumes (open circle, DMSO-treated; open triangle, W54011-treated; white box, only TS15-88 injected; blue box, TS15-88 and tMSLC09030-1 co-injected). (**e**) Staining with 3,3′-diaminobenzidine was used to assess the expression of C5a, N-cadherin, and vimentin in tumor tissues from the TS, TS + CM, and TS + CM + W54011 groups. The scale bars represent 200 µm (top) and 50 µm (bottom), respectively. (**f**) Kaplan–Meier survival analysis of the mouse models. The P-value measured when comparing TS + CM (n = 5) to TS + W54011 (n = 8) was 0.00053, and that when comparing TS + CM (n = 5) to TS + CM + W54011 (n = 10) was 0.032. Statistical analysis was conducted using one-way analysis of variance, followed by Tukey’s post hoc test. Results are presented as means ± standard deviation, with significance indicated by asterisks (*P < 0.05, **P < 0.01, ***P < 0.001), highlighting differences between groups or compared to control conditions.

We aimed to assess whether C5a could enhance tumor malignancy in animal brains and if W54011 could counteract its tumorigenic effects. To achieve this, we established a mouse orthotopic xenograft model with four experimental groups (n = 31). Luminescence was induced by transducing TS15-88 cells with luciferase virus particles, as these cells exhibited relatively high endogenous C5a levels and demonstrated increased sphere proliferation, stemness, and invasive ability following CM treatment. We administered orthotopic injections into the brains of mice and divided the animals into four groups: TS alone (n = 8), TS + W54011 (n = 5), TS co-injected with tMSLCs (TS + tMSLC; n = 8), and TS + tMSLC treated with W54011 (TS + tMSLC + W54011; n = 10) (Fig. 5a).

To mimic a C5a-enriched microenvironment prior to implantation, TS15-88 in the TS + tMSLC and TS + tMSLC + W54011 groups were pre-exposed to tMSLC-derived CM for 24 h. W54011 was subsequently applied at its IC_20_ concentration (7.5 μM) for 72 h—a dose established through prior titration to achieve partial C5aR1 blockade while maintaining cell viability. Trypan blue exclusion was performed to confirm and standardize the number of viable cells prior to injection. This pre-treatment approach was intended to model transient, early-phase C5aR1 inhibition and evaluate its impact on the tumor-initiating potential of GBM tumorspheres, independent of systemic pharmacokinetics or host immune responses. Similar preconditioning strategies have been used in previous GBM studies to investigate the early-stage effects of molecular perturbations on tumor development^35–37^. Approximately one week after cell injection, tumor formation was observed and imaged weekly using IVIS fluorescence, with magnetic resonance imaging (MRI) scans conducted at 18 weeks. Tumor size observed using IVIS fluorescence indicated a significant increase in the TS + tMSLC treatment group compared to that in the TS-only injection group. In contrast, tumor size in the TS + tMSLC + W54011 treatment group was nearly restored to its original size (Fig. 5b). However, this reduction in tumor burden did not correspond to a statistically significant improvement in survival relative to that in the TS-only group. This apparent discrepancy likely reflects the transient nature of tumor suppression by W54011, where early inhibition of C5aR1 may reduce initial tumor growth but fail to fully counteract sustained tumor-promoting cues from co-implanted tMSLCs. These observations were consistent with the MRI results obtained at 18 weeks (Fig. 5c). The analysis is graphically presented in Fig. 5d. In addition, a longitudinal plot of average radiance per group with SEM-derived error bars is provided in Supplementary Fig. S9a to illustrate tumor progression dynamics over the study period.

To investigate whether C5a levels were elevated in the TS + tMSLC group and if this elevation could induce EMT in animal models, we performed 3,3′-diaminobenzidine (DAB) staining to evaluate N-cadherin and vimentin expression and quantified C5a levels. We also assessed the effects of W54011 on these markers. Our findings revealed significant upregulation of N-cadherin, vimentin, and C5a expression in the TS + tMSLC group, indicating activation of the EMT (Fig. 5e). Notably, treatment with W54011 led to a marked reduction in the expression levels of these markers, suggesting its potential as a therapeutic agent to impede EMT progression. W54011 treatment also restored or downregulated the expression of these markers.

**Fig. 6:**
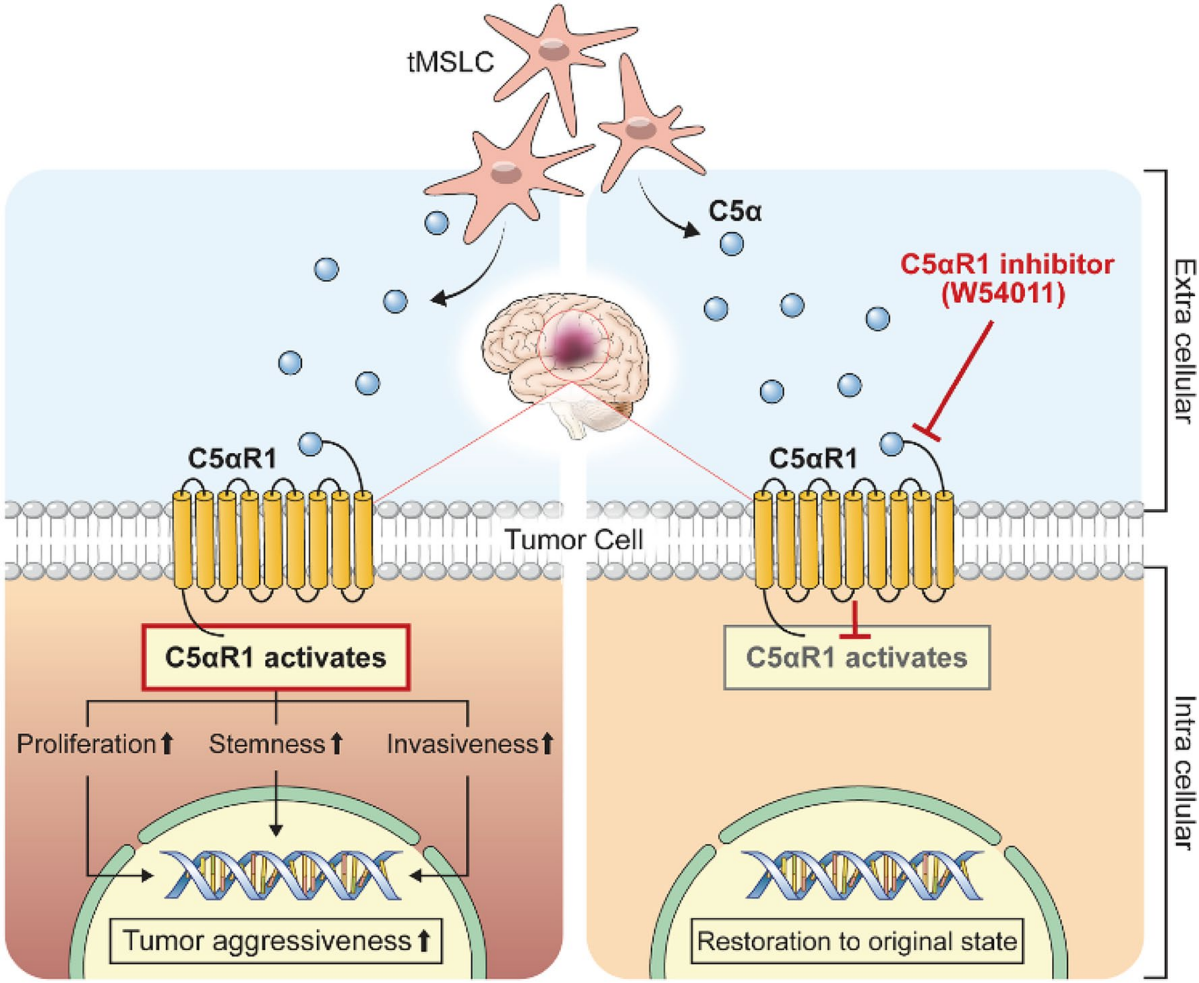
Schematic illustration of W54011-mediated restoration of the C5a-altered glioblastoma tumor microenvironment. Tumor mesenchymal stem-like cells (tMSLCs) secrete C5a, which activates C5aR1 signaling in glioblastoma cells, driving enhanced proliferation, stemness, and invasiveness. This C5a-enriched microenvironment promotes tumor progression and malignant phenotypes. Pharmacologic inhibition of C5aR1 with W54011 suppresses C5a-induced downstream signaling and restores tumor cells toward a less aggressive state.

Additionally, our survival analysis indicated compelling evidence supporting the role of C5a in promoting tumor progression and the therapeutic potential of W54011. Mice injected with tumorspheres alone exhibited significantly better survival than those co-injected with tMSLCs (P = 0.00053). Notably, W54011 treatment in the TS + tMSLC group significantly prolonged survival compared to that in the untreated group (P = 0.032). In contrast, W54011 treatment alone in the tumorsphere group (TS + W54011) did not alter survival in the absence of tMSLCs (TS vs. TS + W54011), confirming its selective activity in C5a-rich microenvironments (Fig. 5f). The survival curves for these two groups were nearly identical, indicating that W54011 does not exert deleterious or pro-tumorigenic effects in the absence of elevated C5a signaling. These results support the hypothesis that C5a may contribute to tumor malignancy in the mouse brain, and that W54011 may mitigate these effects. Together, these data demonstrate that tMSLC-derived C5a activates C5aR1 signaling in glioblastoma to enhance proliferation, stemness, and invasion, whereas W54011 treatment disrupts this signaling and restores tumor cells to a less aggressive state (Fig. 6).

## Discussion

This study implicates C5aR1 as a potential driver of GBM malignancy and highlights the therapeutic potential of the C5aR antagonist, W54011. Elevated C5aR1 expression correlated with poor survival in GBM patient cohorts (Fig. 1). Using patient-derived GBM tumorspheres preconditioned with tMSLC-derived CM, we showed that C5a enhances tumor proliferation, stemness, and invasion (Figs. 2, 3, 4). These effects were markedly attenuated by W54011, both in vitro and in orthotopic mouse models, where the treatment partially restored tumor morphology and improved survival (Fig. 5).

GSCs play a critical role in GBM progression and recurrence, particularly through their interaction with tMSLCs within the TME. Our findings support the concept that tMSLCs potentiate GBM malignancy via autocrine and paracrine mechanisms^17,38^, including the secretion of C5a and other cytokines, thereby presenting a critical therapeutic axis to counteract the malignancy exacerbated by tMSLCs^30,31,39^.

C5a, a cleavage product of complement component C5, has been implicated in angiogenesis and immunosuppression^40^, as well as therapeutic resistance in several cancers. Prior studies have shown that inhibiting C5a signaling has therapeutic potential. For example, apigenin, which blocks C5aR, reduces nasopharyngeal carcinoma cell proliferation^41^, and high C5aR expression is associated with poor prognosis in hepatocellular carcinoma^42^. In GBM, C5a enhances TMZ resistance by promoting the M2 polarization of microglia, which is associated with increased secretion of C5 and C5a. This resistance can be mitigated by C5aR antagonists, such as W54011^43^. Thus, combining W54011 with TMZ could provide synergistic therapeutic benefits. Future research should evaluate the clinical applicability of W54011, particularly in patients with elevated tMSLC levels, to optimize GBM treatment strategies. The selective effect of W54011 on CM-treated tumorspheres suggests that it acts specifically in the presence of heightened C5a signaling, offering a precision medicine approach for patients with tMSLC-rich tumors.

Our findings suggest that targeting C5a secretion from tMSLCs can revert malignant tumor cells to a less aggressive state^10^. Although recombinant C5a modestly enhanced cell viability, tumorsphere formation, and matrigel invasion in GBM tumorspheres (TS15-88, TS14-15, and U87), these effects were consistently less pronounced than those elicited by tMSLC-derived CM. This discrepancy likely reflects the complex composition of the CM. Previous studies, including cytokine array analyses by Lim et al.^10^, have shown that while C5a is the most abundantly secreted factor, CM also contains cytokines, such as IL-6, IL-8, MCP-1, and MIF, that may act synergistically to amplify C5aR1 signaling. These findings support the idea that while C5a is a potential driver, its activity is potentiated by the cytokine-rich environment in the TME.

The GBM TME consists of proliferative astrocytoma cells, endothelial cells, pericytes, and various immune cell populations^44,45^. Effective GBM therapy must target both tumor cells and the surrounding TME due to the inherent heterogeneity of tumors and their immune-evasive nature^46^. Bevacizumab, an anti-VEGF-A monoclonal antibody, has been shown to extend progression-free survival and improve the quality of life of patients with GBM^47^. Similarly, galectin-1—abundant in the GBM TME—drives tumor invasiveness and chemoresistance, and its inhibition enhances TMZ efficacy^48^. Osteopontin, another key TME component, is linked to poor prognosis and promotes tumor growth via the Akt/mTOR/p70S6K signaling pathway^49^. Our findings suggest that targeting C5a secretion from tMSLCs could preserve GBM stem-like traits, offering a potential therapeutic strategy for this aggressive cancer.

However, in our matrigel invasion assays, we observed signs of core shrinkage and structural collapse in spheroids treated with higher concentrations of W54011 (e.g., 7.5 µM), particularly in TS15-88 and TS14-15. These morphological features may indicate early compromise of viability or stress-induced collapse of spheroid structure. Such phenomena are well-documented in 3D tumor spheroid models, where oxygen and nutrient gradients can lead to hypoxia, apoptosis, or necrosis in the spheroid core^50–52^. Although we did not directly assess apoptosis in this study, this remains a potential limitation and warrants further investigation.

Several limitations of the present study should be acknowledged. First, our use of immunodeficient mice precludes the evaluation of the full spectrum of C5aR1-mediated immune responses, particularly those involving microglia and complement-driven immunomodulation. Second, W54011 was applied in vitro as a pre-treatment rather than administered systemically in vivo. While this allowed us to isolate tumor-intrinsic effects of C5aR1 inhibition, it may not fully capture therapeutic dynamics achievable through clinical delivery. Third, the tMSLC-derived CM likely contains additional cytokines, complicating attribution of the observed effects solely to C5a. Lastly, although U87 cells were included under tumorsphere-forming conditions to highlight functional differences between established glioma models and patient-derived tumorspheres, the U87MG line—particularly the ATCC variant—is known to diverge substantially from the primary tumor from which it was originally derived, both in genotype and phenotype. This discrepancy is attributable to long-term in vitro passaging and adaptation to culture conditions. While U87MG remains widely used due to its ease of handling and reproducibility, we interpreted the U87-based results cautiously and placed primary emphasis on the patient-derived GBM models, which better reflect the molecular heterogeneity and stem-like properties of GBM.

In summary, our study highlights the potential role of C5a in GBM malignancy and demonstrates the therapeutic potential of C5aR inhibition by W54011. High C5a levels correlate with poor survival in GBM cohorts (Fig. 1). Our data show that C5a promotes tumor cell proliferation (Fig. 2), stemness (Fig. 3), and invasion (Fig. 4), and it also contributes to reduced survival in mouse models (Fig. 5). W54011 attenuates malignant phenotypes, suppresses tumor growth, and partially restores tumor morphology. Notably, it significantly improves survival in the presence of tMSLCs but not in tumorspheres alone (Fig. 5f). These findings align with those of prior reports implicating C5a in cancer progression and therapeutic resistance, supporting further investigation into the strategy of targeting C5a to counter immune evasion in GBM. Combining W54011 with standard therapies, such as TMZ, may offer synergistic benefits, and its selective use in tMSLC-rich tumors could enable personalized treatment approaches to improve GBM outcomes.

## Methods

### Synthesis of W54011

The C5aR antagonist, W54011, was synthesized in a free form, according to a previously reported synthetic scheme^28^. All reagents and solvents were purchased from chemical suppliers.

### Spectral data of W54011

W54011. ^1^H NMR (400 MHz, CDCl3) d ppm 7.15 (d, *J* = 8.55 Hz, 2 H), 7.18 (d, *J* = 8.24 Hz, 2 H), 6.91–7.03 (m, 3 H), 6.62–6.73 (m, 3 H), 6.53 (d, *J* = 2.44 Hz, 1 H), 5.08 (d, *J* = 14.04 Hz, 1 H), 4.58 (d, *J* = 14.04 Hz, 1 H), 3.65–3.75 (m, 4 H), 2.93–3.03 (m, 6 H), 2.71–2.93 (m, 2 H), 2.59 (dt, *J* = 16.33, 4.50 Hz, 1 H), 1.95–2.09 (m, 2 H), 1.84–1.94 (m, 1 H), 1.39–1.55 (m, 1 H), 1.19–1.29 (m, 6 H). MS (ESI): [M + H]^+^  = 457.2.

### External validation using TCGA data

To validate the findings derived from the Severance cohort, we performed external validation using publicly available data from TCGA via the UCSC Xena platform (https://xena.ucsc.edu). Specifically, we utilized the TCGA GBM dataset, focusing on mRNA expression data (log2(FPKM + 1)) and corresponding clinical annotations. Expression levels of C5aR1 and other relevant immune and stromal genes were extracted and analyzed in the context of GBM molecular subtypes and survival outcomes.

### Patient cohort and derivation of glioblastoma tumorspheres

Freshly resected GBM tissues were obtained from patients diagnosed at the Severance Hospital (Yonsei University College of Medicine, Seoul, Republic of Korea), comprising a newly established cohort for this study (referred to as the Severance cohort). Written informed consent was obtained from all patients prior to sample collection, and all procedures were approved by the Institutional Review Board (IRB No. 4-2021-1319). A total of 96 primary GBM specimens were collected, and the associated clinical information is provided in Supplementary Table S1. Tumor samples were processed to establish patient-derived tumorspheres and tMSLCs. The isolation and culture protocols were adapted from previously validated methodologies developed by our group^10,11,17^. These prior studies demonstrated the robustness and reproducibility of our approach for generating GBM-derived tumorsphere and tMSLC cultures under defined conditions.

Among the established patient-derived cell lines, TS15-88, TS14-15, and tMSLC0903-01 were selected for functional in vivo studies based on prior characterization and sample availability. TS15-88, derived from a 2015 GBM specimen, has been shown to exhibit high tumorigenic potential in orthotopic xenograft models, forming invasive tumors with consistent penetrance and short latency^53–55^. In contrast, TS14-15, established in 2014, demonstrated markedly delayed tumor formation under comparable conditions, with minimal tumor progression over time^53^. Originally referred to as KGS-MSC0504 and derived from a 2009 GBM specimen, tMSLC0903-01 was previously shown to possess tumorigenic potential upon orthotopic implantation^55^. The clinical and molecular characteristics of the original tumor tissues from which these cell lines were derived are summarized in Supplementary Table S3.

As noted in Supplementary Table S3, entries for EGFR, TP53, PTEN, and TERT promoter mutation status are indicated as “NA” due to the absence of routine clinical-grade next-generation sequencing for specimens collected prior to 2016 at our institution. This includes the source tumors for TS15-88 (2015), TS14-15 (2014), and tMSLC0903-01 (2009).

### Isolation of glioblastoma tumorspheres and tumor mesenchymal stem-like cells from tumor samples

Primary tumorspheres and tMSLCs were isolated from freshly resected GBM specimens using a previously established protocol, as described in prior studies employing comparable GBM tissues^9–11,13,56,57^. Briefly, the freshly resected GBM tissues were mechanically dissociated using sterile surgical blades and filtered through 100-μm nylon mesh cell strainers in Dulbecco’s modified Eagle medium: nutrient mixture F-12 (DMEM/F-12; Mediatech, Manassas, VA, USA) supplemented with 1% antibiotic–antimycotic solution (Invitrogen, Waltham, MA, USA). The resulting single-cell suspension was divided equally into two aliquots. One half was cultured in DMEM/F-12 supplemented with B27, 20 ng/mL basic fibroblast growth factor (bFGF), 20 ng/mL epidermal growth factor (EGF), 50 U/mL penicillin, and 50 mg/mL streptomycin (all sourced from Invitrogen) to generate tumorspheres. The other half was cultured in minimal essential medium-α (Mediatech) containing 10% fetal bovine serum (Lonza, Basel, Switzerland), 2 mM l-glutamine (Mediatech), and 1% antibiotic–antimycotic solution (Invitrogen) for the derivation of tMSLCs.

For comparative analysis, the human GBM cell line, U87MG (Korean Cell Line Bank, Seoul, Republic of Korea), was cultured under the same tumorsphere-forming conditions as those used for primary patient-derived GBM tumorspheres. Specifically, U87 cells were maintained in DMEM/F-12 medium supplemented with B27, 20 ng/mL bFGF, 20 ng/mL EGF, 50 U/mL penicillin, and 50 μg/mL streptomycin, consistent with previously published protocols^35,58^. Under these defined conditions, U87MG cells reproducibly formed stable, non-adherent spheres and were used as a comparative tumorsphere model in downstream functional assays. This methodology is consistent with previous reports^59^ and prior studies from our group demonstrating the successful use of U87 cells in tumorsphere-based assays^35,60,61^. NHAs were included as a non-neoplastic reference. NHA cells were purchased from Lonza (Catalog number CC-2565; Walkersville, MD, USA) and cultured in Astrocyte Basal Medium supplemented with the Astrocyte Growth Kit (Lonza), following the manufacturer’s instructions and protocols adapted from previous studies^9,62^. Cells were maintained under standard adherent conditions and used for comparative molecular analyses.

### Gene expression datasets and analysis

Ninety-six samples were obtained from primary patients with GBM who had undergone operations at the Severance Hospital. To obtain gene expression profiles through microarrays, we extracted total RNA from each tissue sample using Qiagen RNeasy Plus Mini kits. The collected RNA was then loaded onto the Illumina HumanHT-12 v4 Expression BeadChip (Illumina, San Diego, CA, USA). The data underwent variance stabilizing transformation and quantile normalization using the R/Bioconductor lumi package^63^. DEGs were calculated using the limma package^63,64^, and the C5aR1 expression level of patients was divided into two groups using the maxstat package^65^. This algorithm, based on Contal & O’Quigley’s log-rank method^66^, examines all possible cut-points and chooses the one that maximizes the difference in overall survival while correcting for multiple testing. Patients with C5aR1 expression above this cut-point were designated as the “high” group, and those with expression at or below the cut-point as the “low” group. In our in-house cohort, molecular subtypes were assigned based on transcriptomic profiles using published classifiers. Verhaak subtypes (Classical, Mesenchymal, and Proneural) were determined according to established gene expression signatures, and TME subtypes (TME-High, TME-Med, and TME-Low) annotated based on the classification framework proposed by White et al. (Cell, 2020)^26,27^.

A volcano plot was generated using the EnhancedVolcano package. Genes were functionally annotated via overrepresentation analysis using GO gene sets and then visualized as a dot plot using the clusterProfiler package. Enriched GO terms were categorized according to their kappa scores (> 0.4). An enrichment plot was generated using GenePattern v.2.0^67^. The datasets generated and/or analyzed during the current study are available from the corresponding author upon reasonable request.

### Preparation of C5a-enriched conditioned media

The tMSLCs were cultured to achieve a confluency of 80%. After 24 h, the tMSLCs were washed twice with phosphate-buffered saline (PBS; Mediatech) to remove non-adherent cells and the tMSLC media. The culture media was then replaced with tumorsphere media, and the cells allowed to grow for three days. Thereafter, the tumorsphere media were harvested and centrifuged at 1000×*g* for 5 min. The supernatant was collected and used as C5a-enriched CM, which served as the culture media throughout the experimental process.

In subsequent assays, the condition designated as “CM + 0 W54011” refers to cells exposed to tMSLC-derived CM without the addition of W54011 (0 μg/mL). This group served as a positive control to evaluate the full biological impact of C5a signaling in the absence of receptor blockade. All conditions involving CM, including those with or without W54011, were cultured for a total of 96 h—comprising 24 h of CM preconditioning, followed by 72 h of treatment.

### C5a measurements

Human C5a levels were quantified in CM derived from tMSLCs. Tumorspheres were dissociated into single cells, and 1 × 10^4^ cells/well seeded into a 96-well plate. Following a 24-h incubation period, W54011 was administered at various concentrations in the CM for 72 h. The concentration of C5a was determined using the Human Complement Component C5a DuoSet ELISA kit (R&D Systems, Minneapolis, MN, USA), following the manufacturer’s protocol.

### Cell viability and ATP level assays

Cell viability and ATP level assays were conducted to evaluate the impact that CM and W54011 have on cell proliferation. Tumorspheres were dissociated into single cells, and 1 × 10^4^ cells/well plated into a 96-well plate. After 24 h of incubation, W54011 was treated at various concentrations in CM for 72 h. Cell viability was assessed using the WST-8/CCK-8 assay by measuring absorbance at 450 nm after adding 10 μL WST-8/CCK8 (Dojindo Laboratories, Kumamoto, Japan). ATP levels were quantified using the CellTiter-Glo Luminescent Cell Viability Assay Kit (Promega, Madison, WI, USA) by adding 70 μL of the reagent to each well and measuring luminescence. All measurements were performed in triplicate, and statistical analysis conducted using Prism v.8.0 software (GraphPad Software, La Jolla, CA, USA).

### Western blotting analysis

Tumorspheres were collected and lysed in lysis buffer (50 mM Tris–Cl [pH 8.0], 150 mM Nonidet P-40 (NP-40), 150 mM NaCl, 0.1% sodium dodecyl sulfate [SDS], 0.5% deoxycholic acid) supplemented with phosphatase and protease inhibitor cocktails (GenDEPOT, Altair, TX, USA). The lysates were collected after centrifugation at 14,000 rpm at 4 °C for 15 min. Protein quantification was performed using the Bradford reagent (Bio-Rad, Hercules, CA, USA), and the proteins were separated by SDS–polyacrylamide gel electrophoresis. The separated proteins were transferred to nitrocellulose membranes (GE Healthcare Life Sciences, Chicago, IL, USA) and obstructed with 3% bovine serum albumin (BSA) for 1 h. Subsequently, the membranes were incubated overnight at 4 °C with specific primary antibodies at a dilution of 1:1000. The membranes were visualized using Western Lightning Plus-enhanced chemiluminescence reagent (PerkinElmer, Waltham, MA, USA), and the images captured using an ImageQuant LAS 4000 mini camera system (GE Healthcare Life Sciences). Membranes were cut prior to antibody hybridization to enable probing of different regions with specific antibodies; uncropped blot images for the available replicates, showing the corresponding membrane areas, are presented in Supplementary Figs. S10–S12.

### Extreme limiting dilution analysis assay

Dissociated single tumorspheres were seeded into a 96-well plate at densities ranging from 10–200 cells/well (n = 30). The tumorspheres were then exposed to either tumorsphere complete media, CM, or W54011 in CM. After 14 days of incubation, large images with a diameter greater than 50 μm were captured and quantified using the Operetta CLS High-Content Analysis System (PerkinElmer). Subsequently, ELDA analysis (http://bioinf.wehi.edu.au/software/elda/) based on tumorigenic cell frequency was performed to determine statistical significance.

### Neurosphere formation assay

GBM tumorspheres were resuspended in 500 μL Accutase (Thermo Fisher Scientific, Waltham, MA, USA) for single-cell dissociation and then plated at a density of 10 cells/well in a 96-well plate. After a three-week incubation period, the wells were imaged using a brightfield microscope (KI-400; Korea Lab Tech, Seongnam, Republic of Korea), and the images were analyzed with ToupView software (ToupTek Photonics, Hangzhou, China). The formation of spheres and their radii were measured and compared.

### Immunocytochemistry

Tumorspheres were attached to coverslips (VectorLabs, Newark, CA, USA) using poly-l-lysine (Sigma-Aldrich, St. Lois, MO, USA) and incubated for 24 h. Subsequently, the tumorspheres were treated with tumorsphere complete media, CM, or CM containing W54011. After 72 h, the cells were fixed in 3.8% formaldehyde/1× PBS and permeabilized using 0.1% NP-40/1× PBS for 15 min. Blocking was performed with 1% BSA/1× PBS for 1 h. Immunostaining was carried out using primary antibodies against GFAP (Cell Signaling Technology, Danvers, MA, USA) and C5a (Abcam, Cambridge, UK), each diluted 1:1000 in blocking buffer. Following primary incubation, cells were stained with Alexa Fluor 488- and Alexa Flour 568-conjugated secondary antibodies (Invitrogen), each diluted 1:1000. Nuclei were counterstained with DAPI at a dilution of 1:10,000. Laser scanning micrographs were acquired using an inverted confocal microscope (LSM700; Zeiss, Oberkochen, Germany). The acquired images were then processed using ZEN2009 software and ImageJ for further analysis.

### Matrigel invasion assay

Tumorspheres were cultured and manually selected under an inverted microscope (Intron Biotechnology, Seongnam, Republic of Korea) to ensure a comparable initial size across all conditions. Selected spheroids were embedded in a 3D matrix composed of 2.4 mg/mL high-concentration rat tail collagen type I (BD Biosciences, Franklin Lakes, NJ, USA), 2.1 mg/mL Matrigel (Corning Life Sciences, Corning, NY, USA), 10% NaHCO_3_, and 2 × tumorsphere complete medium. Embedded tumorspheres were seeded in either tumorsphere complete medium or tMSLC-derived CM and subsequently treated with varying concentrations of W54011. Cultures were incubated for 72 h at 37 °C.

Brightfield images were acquired at two time points: immediately after embedding (0 h, baseline) and after 72 h. Invasion was quantified using ToupView image analysis software (× 64 v3.7.1460; AmScope, Irvine, CA, USA) by measuring the entire area occupied by the tumorsphere and surrounding invading cells. To account for variability in initial spheroid size, the area measured at 72 h was normalized to the baseline area at 0 h, and invasion was expressed as a fold-change relative to the original tumorsphere size. This approach enabled consistent quantification of invasive outgrowth across all conditions.

### Bioluminescent tagging of TS15-88

TS15-88 cells were transduced with a CMV promoter-driven firefly luciferase-expressing lentiviral vector (CMV-Firefly Luciferase Lentivirus, puromycin selection, 1 × 10^8^ TU/mL; Cat. No. PLV-1003; Cellomics Technology, Halethorpe, MD, USA), as previously described^35^. Cells were plated in complete growth medium and incubated with 12.5 μL viral supernatant in the presence of 8 μg/mL polybrene (Sigma, Dorset, UK) to enhance transduction efficiency. Following an 18-h incubation, the medium was replaced with fresh growth medium. Transduced cells were selected with 1 mg/mL puromycin (Life Technologies Korea, Seoul, Republic of Korea) and expanded under standard culture conditions. Successful lentiviral integration and luciferase expression were confirmed through bioluminescence imaging using the IVIS system prior to in vivo application.

### Mouse orthotopic xenograft model

Male athymic nude mice (Central Lab. Animal Inc., Seoul, Republic of Korea) aged 4–8 weeks were used in this experiment. Upon arrival, mice of similar weight and condition were randomly allocated to experimental groups by cage assignment to minimize allocation bias. The animals were acclimatized for at least one week under sterile conditions in a controlled environment maintained at a temperature of 24 ± 0.5 °C and relative humidity of 55–65%, with a 12-h light–dark cycle. All experimental procedures were approved by the Institutional Animal Care and Use Committee of the Yonsei University School of Medicine (Seoul, Republic of Korea). Only male mice were used to minimize potential biological variability in orthotopic tumor growth and survival kinetics that may arise from sex hormone fluctuations. This decision was made to ensure experimental consistency when evaluating tumor-initiating capacity and treatment response using patient-derived xenograft models, in accordance with commonly adopted practices in preclinical GBM studies.

For orthotopic implantation, luciferase-tagged TS15-88 (5 × 10^5^) or tMSLC0903-01 (5 × 10^5^), or a 1:1 mixture of TS15-88 (2.5 × 10^5^) and tMSLC0903-01 (2.5 × 10^5^), were injected into the right frontal lobe of the nude mice using an implantable guide-screw system^68^. Injections were made at 2.5 mm lateral and 1.0 mm anterior to the bregma, at a depth of 3.5 mm from the skull surface to target the striatal region. To investigate the cell-intrinsic effects of C5aR1 inhibition under early microenvironmental priming, tumorspheres were pre-exposed to tMSLC-derived CM for 24 h. W54011 was then administered at its IC_20_ concentration (7.5 μM) for an additional 72 h, based on prior titration to enable partial C5aR1 blockade while preserving viability. This pre-treatment was performed prior to orthotopic injection to mimic transient C5a-enriched priming and assess its influence on tumor-initiating capacity. Viability was assessed through trypan blue exclusion, and equal numbers of viable cells were injected into all animals.

While tMSLC-derived CM was used for in vitro functional assays, CM was not administered in vivo. Instead, live tumorspheres and tMSLCs were co-injected directly to model direct tumor–stromal interactions in the GBM microenvironment. This strategy was chosen to more closely mimic the clinical setting, in which tMSLCs coexist with GSCs within the tumor niche. A similar co-injection model has been previously established to recapitulate clinically relevant GBM–stroma crosstalk ^11^. For relevant treatment groups, tumorspheres or tMSLCs were pre-treated with W54011, and the live cells were selected with trypan blue.

Mice were monitored every other day for general health and body weight. Animals that lost more than 15% of their maximum body weight were euthanized in accordance with human endpoint criteria. At the experimental endpoint or upon euthanasia, the mouse brains were collected, fixed in 10% neutral-buffered formalin, embedded in paraffin, and cut into 4 µm-thick slices. Tissue sections were deparaffinized in xylene and stained with hematoxylin. Imaging was captured using an IX71 microscope (Olympus, Tokyo, Japan).

Sample sizes were initially chosen as n = 5 per group based on prior pilot studies and commonly adopted standards in GBM xenograft experiments. Group sizes were subsequently increased (up to n = 10) in selected experimental arms to support subgroup comparisons and ensure sufficient statistical power.

### Mouse imaging

Bioluminescent signals were acquired and analyzed using the IVIS Spectrum in vivo imaging system, employing Living Image v.4.2 software (Caliper Life Sciences, Hopkinton, MA, USA). Mice were anesthetized with 2.5% isoflurane and received an intraperitoneal injection of d-luciferin (30 mg/mL dissolved in PBS, 100 μL; Promega) 15 min before signal capture, which lasted for 5 s. IVIS imaging was performed every three weeks, beginning one week after orthotopic cell injection, to monitor intracranial tumor progression throughout the study period. Regions of interest (ROIs) were drawn over the tumor site, and signals expressed as mean radiance (photons/s/cm^2^/sr). Researchers were not blinded to group allocation due to experimental handling and cell preparation requirements; however, outcome measures, such as IVIS flux and survival, were based on objective, quantifiable endpoints.

MRI was performed using a 9.4-T small-animal scanner (BioSpec 94/20 USR; Bruker BioSpin GmbH, Ettingen, Germany). T2-weighted Turbo RARE sequences were acquired with the following parameters: TR = 2000 ms, TE = 30 ms, flip angle = 90°, averages = 2, matrix size = 192 × 192, in-plane resolution = 0.104 × 0.104 mm/pixel, field of view = 20 × 20 mm, slice thickness = 0.5 mm, 20 slices, and scan time = 2 min 8 s. A transmit coil (Tx 86 mm) and mouse brain surface receive coil (Rx) were used. Mice were anesthetized with isoflurane (induction 3%, maintenance 2%) during imaging. No contrast agent was administered. Tumor burden was quantified through manual segmentation of hyperintense lesions on T2-weighted images using ImageJ, and tumor volume was calculated by summing lesion areas across slices.

### Immunohistochemistry

Paraffin-embedded tissue blocks were sectioned at 5-μm thicknesses using a microtome and mounted on adhesive glass slides. Immunohistochemical staining was performed using a peroxidase/DAB detection system. Primary antibodies against C5a (Abcam), N-cadherin (Cell Signaling Technology), and vimentin (Abcam) were applied at a dilution of 1:500. Stained sections were imaged using an IX71 microscope.

### Statistical analysis

Statistical analysis was performed using Prism (v.8.0) and R software (v.4.2.0) with the following packages: survival (3.5–5), maxstat (0.7–25), limma (3.52.4), lumi (2.46.0), dplyr (1.1.2), ggplot2 (3.4.2), and clusterProfiler (4.6.2). For multiple comparisons, one-way analysis of variance, followed by Tukey’s post hoc test, were applied. Mice survival was analyzed using the Kaplan–Meier method and log-rank test. Statistical significance was determined with P-value thresholds of *P < 0.05, **P < 0.01, and ***P < 0.001.

## Supplementary Information

Supplementary Information.

## Data Availability

The datasets generated and/or analyzed during the current study are not publicly available due to the risk of compromising individual privacy but are available from the corresponding author on reasonable request and with an appropriate, agreed-upon collaboration.
