# Supplementary material for: Restoring the glioblastoma tumor microenvironment by targeting C5a with the antagonist W54011
Source: Sci Rep. 2025 Dec 6;15:44428. doi: 10.1038/s41598-025-30853-1 (PMC12738741; doi:10.1038/s41598-025-30853-1)
Supplement: Supplementary file 1 — Supplementary Information. [file 41598_2025_30853_MOESM1_ESM.docx]

**Restoring the glioblastoma tumor microenvironment by targeting C5a with the antagonist W54011**

Yoojung Oh^1,2,3†^, Jihwan Yoo^4†^, Dongkyu Lee^1,2,3^, Bongki Ko^5^, Jun Pyo Hong^1,2,3^, Ju Hyung Moon^1,3^, Eui Hyun Kim^1,3^, Jong Hee Chang^1,3^, Yong-Chul Kim^5*^, Seok-Gu Kang^1,2,3,6*^

^1^Department of Neurosurgery, Brain Tumor Center, Severance Hospital, Yonsei University College of Medicine, Seoul 03722, Republic of Korea

^2^Brain Tumor Translational Research Laboratory, Department of Biomedical Sciences, Yonsei University College of Medicine, Seoul 03722, Republic of Korea

^3^Brain Research Institute, Yonsei University College of Medicine, Seoul 03722, Republic of Korea

^4^Department of Neurosurgery, Gangnam Severance Hospital, Yonsei University College of Medicine, Seoul, 06273, Republic of Korea

^5^School of Life Sciences, Gwangju Institute of Science & Technology, Gwangju 61005, Republic of Korea

^6^Department of Neurosurgery, Graduate School of Medical Science, Brain Korea 21 Project, Yonsei University College of Medicine, Seoul 03722, Republic of Korea

^†^These authors contributed equally to this work.

^*^Corresponding authors:

Seok-Gu Kang, M.D., Ph.D., Department of Neurosurgery, Brain Tumor Center, Severance Hospital, Yonsei University College of Medicine, 50-1 Yonsei-ro, Seodaemun-gu, Seoul 03722, Republic of Korea; Tel.: +82-2-2228-2150; E-mail: [seokgu9@gmail.com](mailto:seokgu9@gmail.com)

Yong-Chul Kim, Ph.D., School of Life Sciences, Gwangju Institute of Science & Technology, 123 Cheomdangwagi-ro, Buk-gu, Gwangju 61005, Republic of Korea; Tel.: 062-715-2502; E-mail: [yongchul@gist.ac.kr](mailto:yongchul@gist.ac.kr)

**Supplementary tables**

**Supplementary Table S1.** Clinical information of the 96 glioblastoma samples used in this study

| **Characteristics** | **C5α High (n = 61)** | **C5α Low (n = 35)** | **P-value** |
| --- | --- | --- | --- |
| Age (years) | 60.0 ± 10.8 | 59.5 ± 12.3 | 0.823 |
| Sex |  |  | 0.274 |
| F, n (%) | 23 (37.7%) | 18 (51.4%) |  |
| M, n (%) | 38 (62.3%) | 17 (48.6%) |  |
| Pathological diagnosis |  |  | 1.000 |
| Glioblastoma, n (%) | 61 (100.0%) | 35 (100.0%) |  |
| Type |  |  | 1.000 |
| Primary, n (%) | 61 (100.0%) | 35 (100.0%) |  |
| Recurrent, n (%) | 0 (0.0%) | 0 (0.0%) |  |
| IDH mutation |  |  | 1.000 |
| Wild type, n (%) | 61 (100.0%) | 35 (100.0%) |  |
| Mutant, n (%) | 0 (0.0%) | 0 (0.0%) |  |
| MGMT promotor methylation |  |  | 0.169 |
| Methylated, n (%) | 18 (29.5%) | 16 (45.7%) |  |
| Unmethylated, n (%) | 43 (70.5%) | 19 (54.3%) |  |
| 1p19q codeletion |  |  | 0.777 |
| No deletion, n (%) | 61 (100.0%) | 34 (97.1%) |  |
| Codeletion, n (%) | 0 (0.0%) | 0 (0.0%) |  |
| TERT mutation |  |  | 0.015^*^ |
| C228T, n (%) | 30 (49.2%) | 9 (25.7%) |  |
| C250T, n (%) | 9 (14.8%) | 4 (11.4%) |  |
| Wild type, n (%) | 5 (8.2%) | 11 (31.4%) |  |
| NA, n (%) | 17 (27.9%) | 11 (31.4%) |  |
| EGFR mutation |  |  | 0.339 |
| Yes, n (%) | 24 (39.3%) | 15 (42.9%) |  |
| No, n (%) | 30 (49.2%) | 19 (54.3%) |  |
| NA, n (%) | 7 (11.5%) | 1 (2.9%) |  |
| Ki-67 index, (%) | 24.2 ± 15.6 | 30.8 ± 18.4 | 0.067 |

EGFR: epidermal growth factor receptor; F: female; IDH: isocitrate dehydrogenase; M: male; MGMT: *O*-6-methylguanine-DNA methyltransferase; TERT: telomerase reverse transcriptase.

The value with statistical significance is marked by an asterisk (^*^).**Supplementary Table S2.** Distribution of Verhaak molecular subtypes and White TME classifications according to C5aR1 expression status (high vs. low)

| **Classification** | **C5a High** | **C5a Low** | **P-value** |
| --- | --- | --- | --- |
|  | **(n = 61)** | **(n = 35)** |  |
| Verhaak classification |  |  | <0.001 |
| Classical | 37 (60.7%) | 19 (54.3%) |  |
| Mesenchymal | 17 (27.9%) | 1 (2.9%) |  |
| Proneural | 7 (11.5%) | 15 (42.9%) |  |
| White classification |  | | <0.001 |
| TME High | 26 (42.6%) | 0 (0.0%) |  |
| TME Low | 8 (13.1%) | 22 (62.9%) |  |
| TME Med | 27 (44.3%) | 13 (37.1%) |  |

TME: tumor microenvironment.

Statistical significance was defined as p <0.05.

**Supplementary Table S3.** Clinical and mutational profiles of two tumor tissues selected for detailed comprehensive experiments

| **Case number** | **15-88**  **(8127365)** | **14-15**  **(2145869)** | **0903-01**  **(5838381)** |
| --- | --- | --- | --- |
| Diagnosis | Glioblastoma | Glioblastoma | Glioblastoma |
| IDH type | Wild type | Wild type | Wild type |
| MGMT promoter methylation | Unmethylated | Methylated | NA |
| Age | 69 | 68 | 39 |
| Sex | Male | Male | Male |
| 1p/19q status | Intact | 1p intact/19q LOH |  |
| Ki-67 (%) | 30% | 15~20% |  |
| P53 | 80% | 3~4% |  |
| Mutation status | NA | NA | NA |
| TERT promoter mutation | NA | NA | NA |
| EGFR | NA | NA | NA |
| TP53 | NA | NA | NA |
| PTEN | NA | NA | NA |
| Nestin | NA | NA | NA |
| Musashi-1 | NA | NA | NA |
| CD133 | NA | NA | NA |
| SOX2 | NA | NA | NA |
| N-cadherin | NA | NA | NA |
| CD44 | NA | NA | NA |

EGFR: epidermal growth factor receptor; IDH: isocitrate dehydrogenase; MGMT: *O*-6-methylguanine-DNA methyltransferase; TERT: telomerase reverse transcriptase.

**Note:** NA = not available. Molecular profiling (e.g., targeted sequencing of EGFR, TP53, PTEN, TERT promoter, and selected stemness markers) was not routinely performed at our institution for specimens collected prior to 2016. Therefore, data for these entries are unavailable for samples 15-88 (2015), 14-15 (2014), and 0903-01 (2009).

**Supplementary Table S4.** Detailed confidence intervals for the reciprocal of stem cell frequency in three glioblastoma tumorspheres (TS15-88, TS14-15, and U87)

**TS15-88**

| **Group** | **Lower** | **Estimate** | **Upper** |
| --- | --- | --- | --- |
| Control | 15.23 | 11.19 | 8.26 |
| 0 μM | 8.29 | 5.76 | 4.06 |
| 2.5 μM | 10.67 | 7.57 | 5.42 |
| 5 μM | 14.07 | 10.20 | 7.43 |
| 7.5 μM | 25.64 | 19.57 | 14.96 |

**TS14-15**

| **Group** | **Lower** | **Estimate** | **Upper** |
| --- | --- | --- | --- |
| Control | 37.0 | 27.9 | 21.0 |
| 0 μM | 28.0 | 21.9 | 16.8 |
| 2.5 μM | 36.3 | 27.2 | 20.4 |
| 5 μM | 38.7 | 29.2 | 22.0 |
| 7.5 μM | 44.3 | 35.0 | 27.7 |

**U87**

| **Group** | **Lower** | **Estimate** | **Upper** |
| --- | --- | --- | --- |
| Control | 13.43 | 9.22 | 6.38 |
| 0 μM | 6.30 | 4.09 | 2.74 |
| 2.5 μM | 6.93 | 4.50 | 3.00 |
| 5 μM | 19.76 | 14.12 | 10.13 |
| 7.5 μM | 50.33 | 38.58 | 29.60 |

**Supplementary Table S5.** Detailed confidence intervals for the reciprocal of stem cell frequency in TS15-88 under control, CM-SC, and CM-siC5 conditions

**TS15-88**

| **Group** | **Lower** | **Estimate** | **Upper** |
| --- | --- | --- | --- |
| Control | 24.0 | 14.25 | 8.56 |
| CM-Sc | 11.1 | 5.74 | 3.11 |
| CM-siC5 | 60.4 | 40.15 | 26.74 |

**Supplementary Methods**

***Isolation of glioblastoma tumorspheres and tumor mesenchymal stem-like cells from tumor samples***

Primary tumorspheres and tumor mesenchymal stem-like cells (tMSLCs) were isolated from freshly resected glioblastoma (GBM) specimens using a previously established protocol, as described in prior studies employing comparable GBM tissues^1-6^. Briefly, freshly resected GBM tissues were mechanically digested using sterile surgical blades and filtered through 100-μm nylon mesh cell strainers in Dulbecco’s modified Eagle medium: nutrient mixture F-12 (DMEM/F-12; Mediatech, Manassas, VA, USA) supplemented with 1% antibiotic-antimycotic solution (Invitrogen, Waltham, MA, USA). The resulting single-cell suspension was divided equally into two aliquots. One half was cultured in DMEM/F-12 supplemented with B27, 20 ng/mL basic fibroblast growth factor, 20 ng/mL epidermal growth factor, 50 U/mL penicillin, and 50 mg/mL streptomycin (all sourced from Invitrogen) to generate tumorspheres. The other half was cultured in minimal essential medium-α (Mediatech) containing 10% fetal bovine serum (Lonza, Basel, Switzerland), 2 mM l-glutamine (Mediatech), and 1% antibiotic-antimycotic solution for the derivation of tMSLCs.

***Co-culture and conditioned medium collection***

TS15-88 (1.5 × 10⁵) were seeded in the lower chambers of 6-well Transwells (24 mm diameter, 0.4 µm pore; Corning, NY, USA) in serum-free DMEM/F12 supplemented with B27, EGF (20 ng/mL), bFGF (20 ng/mL), 50 U/mL penicillin, and 50 µg/mL streptomycin. tMSLC0903-01 (2 × 10⁵) were plated in the upper inserts in α-MEM containing 10% FBS, 2 mM L-glutamine (Mediatech), and 1% antibiotic–antimycotic solution. The lower wells and inserts contained 2.0 mL and 1.5 mL of medium, respectively. After 72 h at 37 °C, conditioned media from both chambers were pooled, clarified through a 0.45 µm filter, and stored at −80 °C until analysis. For single-culture controls, tMSLC0903-01 (2 × 10⁵) were seeded in the upper insert with 1.5 mL α-MEM complete medium, while the lower chamber contained 2.0 mL of the same medium without cells. Conditioned media from both compartments were pooled, filtered (0.45 µm), and stored at −80 °C.

***siRNA transfection***

Cells were transfected with **AccuTarget™ genome-wide predesigned siRNAs** targeting human **C5** or a non-targeting control siRNA (Bioneer, Daejeon, Korea) using **Lipofectamine 3000** (Invitrogen, Carlsbad, CA, USA) following the manufacturer’s protocol. Knockdown efficiency was evaluated by **cytokine array analysis** (Proteome Profiler Human Cytokine Array, R&D Systems, ARY005B), comparing conditioned media from C5 siRNA–transfected cells with those from control siRNA–transfected cells

***Cytokine array***

Cytokine profiling was performed using the Proteome Profiler Human Cytokine Array Kit (Panel A, ARY005B; R&D Systems, Minneapolis, MN, USA), according to the manufacturer’s instructions. Conditioned media (CM) were incubated overnight at 4 °C with pre-blocked nitrocellulose membranes, followed by incubation with biotinylated detection antibodies and streptavidin–HRP. Signals were visualized by using the ImageQuant LAS 4000 mini (GE Healthcare, Chicago, IL, USA) device. Spot intensities were quantified through densitometry using ImageJ software (v.1.53; National Institutes of Health, Bethesda, MD, USA), normalized to internal positive controls, and expressed as relative fold-changes compared with a control.

***Molecular docking***

Molecular docking analysis used the co-crystal structure of C5aR and NDT9513727 (PDB ID: 5O9H) obtained from the Protein Data Bank. The protein preparation process was carried out through the Protein Preparation Wizard included in Discovery Studio, and a radius of 11 Å around the NDT9513727 binding pocket set as a binding site. For rigid docking, W54011 applied with a CHARMM force field was docked into the above prepared hC5aR structure using the CDOCKER protocol in Discovery Studio.

***Microarray***

We followed the protocols described in Park et al.^3^. Total RNA was extracted from GBM and matched patient cortex tissues, as well as tumorspheres, using the RNeasy Plus Mini Kit (Qiagen, Hilden, Germany), according to the manufacturer’s instructions, and hybridized to the HumanHT-12 v4 Expression BeadChip (Illumina, San Diego, CA, USA). Raw data were processed with the R/Bioconductor lumi package, applying variance-stabilizing transformation and quantile normalization to obtain normalized expression values.

***Cell viability and ATP level assays***

Cell viability and ATP level assays were conducted to evaluate the impact that CM and W54011 have on cell proliferation. Tumorspheres were dissociated into single cells, and 1 × 10^4^ cells/well plated into a 96-well plate. After 24 h of incubation, W54011 was treated at various concentrations in CM for 72 h. Cell viability was assessed using the WST-8/CCK-8 assay by measuring absorbance at 450 nm after adding 10 μL WST-8/CCK8 (Dojindo Laboratories, Kumamoto, Japan). ATP levels were quantified using the CellTiter-Glo Luminescent Cell Viability Assay Kit (Promega, Madison, WI, USA) by adding 70 μL of the reagent to each well and measuring luminescence. All measurements were performed in triplicate, and statistical analysis conducted using Prism v.8.0 software (GraphPad Software, La Jolla, CA, USA).

***Matrigel invasion assay***

Tumorspheres were cultured and manually selected under an inverted microscope (Intron Biotechnology, Seongnam, Republic of Korea) to ensure a comparable initial size across all conditions. Selected spheroids were embedded in a three-dimensional matrix composed of 2.4 mg/mL high-concentration rat tail collagen type I (BD Biosciences, Franklin Lakes, NJ, USA), 2.1 mg/mL Matrigel (Corning Life Sciences, Corning, NY, USA), 10% NaHCO_3_, and 2× tumorsphere complete medium. Embedded tumorspheres were seeded in either tumorspheres complete medium or tMSLC-derived CM and subsequently treated with varying concentrations of W54011. Cultures were incubated for 72 h at 37 °C.

Brightfield images were acquired at two time points: immediately after embedding (0 h, baseline) and after 72 h. Invasion was quantified using ToupView image analysis software (x64 v3.7.1460; AmScope, Irvine, CA, USA) by measuring the entire area occupied by the tumorsphere and surrounding invading cells. To account for variability in initial spheroid size, the area measured at 72 h was normalized to the baseline area at 0 h, and invasion expressed as a fold-change relative to the original tumorsphere size. This approach enabled consistent quantification of invasive outgrowth across all conditions.

***Neurosphere formation assay***

GBM tumorspheres were resuspended in 500 μL Accutase (Thermo Fisher Scientific, Waltham, MA, USA) for single-cell dissociation and then plated at a density of 10 cells/well in a 96-well plate. After a three-week incubation period, the wells were imaged using a brightfield microscope (KI-400; Korea Lab Tech, Seongnam, Republic of Korea), and the images analyzed with ToupView software (ToupTek Photonics, Hangzhou, China). The formation of spheres and their radii were measured and compared.

***Extreme limiting dilution analysis assay***

Dissociated single tumorspheres were seeded into a 96-well plate at densities ranging from 10–200 cells/well (n = 30). The tumorspheres were then exposed to either tumorspheres complete media, CM, or W54011 in CM. After 14 days of incubation, large images with a diameter greater than 50 μm were captured and quantified using the Operetta CLS High-Content Analysis System (PerkinElmer, Waltham, MA, USA). Subsequently, ELDA analysis (http://bioinf.wehi.edu.au/software/elda/) based on tumorigenic cell frequency was performed to determine statistical significance.

***Mouse imaging***

Bioluminescent signals were acquired and analyzed using the IVIS Spectrum in vivo imaging system, employing Living Image v.4.2 software (Caliper Life Sciences, Hopkinton, MA, USA). Mice were anesthetized with 2.5% isoflurane and received an intraperitoneal injection of d-luciferin (30 mg/mL dissolved in PBS, 100 μL; Promega) 15 min before signal capture, which lasted for 5 s. IVIS imaging was performed every three weeks, beginning one week after orthotopic cell injection, to monitor intracranial tumor progression throughout the study period. Regions of interest (ROIs) were drawn over the tumor site, and signals expressed as mean radiance (photons/s/cm²/sr). Researchers were not blinded to group allocation due to experimental handling and cell preparation requirements; however, outcome measures, such as IVIS flux and survival, were based on objective, quantifiable endpoints.

***Statistical analysis***

Statistical analysis was performed using Prism (v.8.0) and R software (v.4.2.0) using the following packages: survival (3.5-5), maxstat (0.7-25), limma (3.52.4), lumi (2.46.0), dplyr (1.1.2), ggplot2 (3.4.2), and clusterProfiler (4.6.2). For multiple comparisons, one-way analysis of variance, followed by Tukey’s post hoc test, were applied. Mice survival was analyzed using the Kaplan–Meier method and log-rank test. Statistical significance was determined with P-value thresholds of *P <0.05, **P <0.01, and ***P <0.001, NS = not significant.

**Supplementary figures**

**
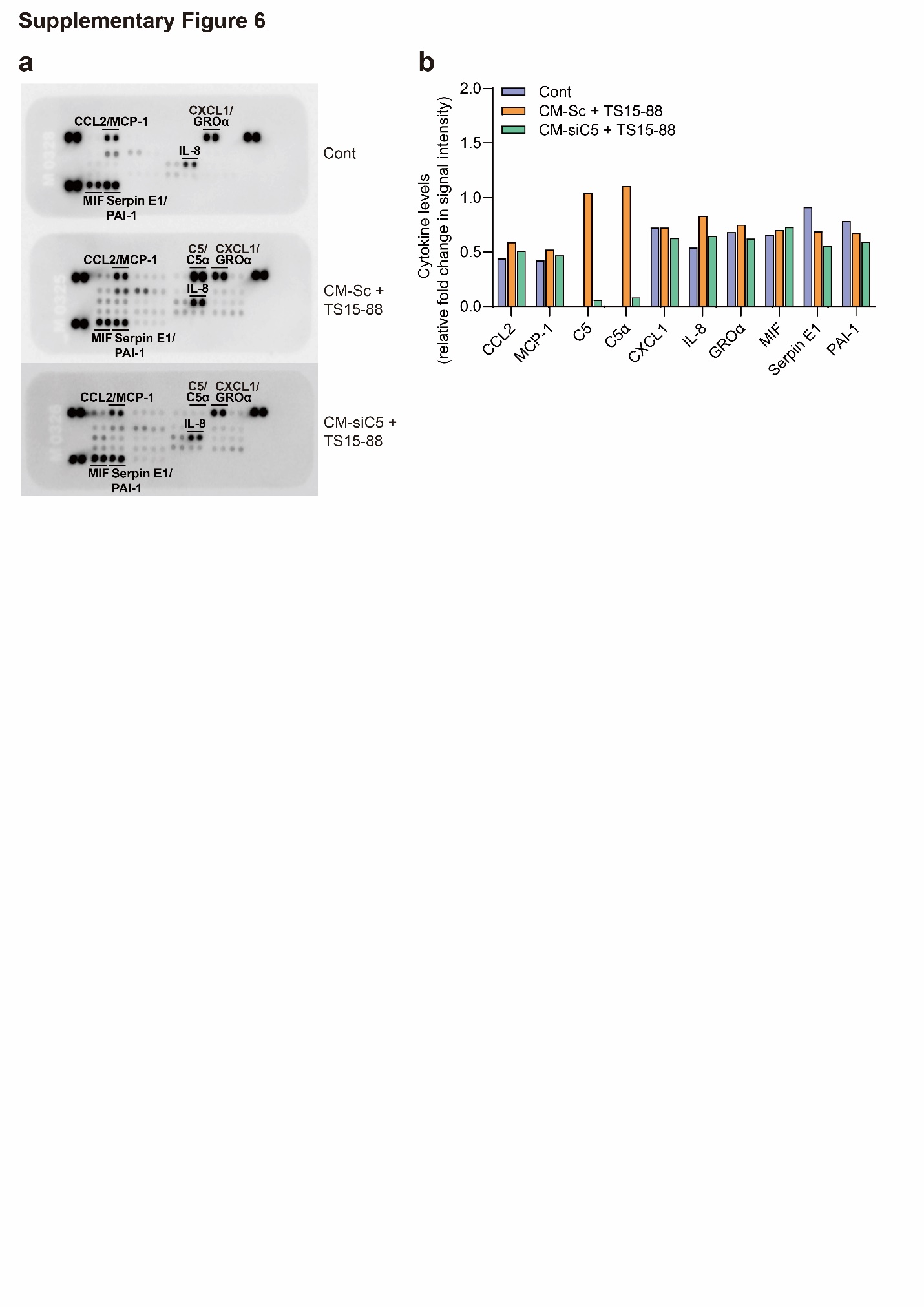
Supplementary Fig. S1. Cytokine profiling of conditioned media from tMSLC0903-01 co-cultured with TS15-88. a** Cytokine array of conditioned media (CM) from tMSLC0903-01 under three conditions: control CM (Cont), CM from scrambled siRNA–transfected tMSLC0903-01 co-cultured with TS15-88 (Sc CM + TS15-88), and CM from C5 siRNA–transfected tMSLC0903-01 co-cultured with TS15-88 (siC5 CM + TS15-88) (n = 1). **b** Quantification of cytokine signal intensities from (**a**) using ImageJ. Values are shown as relative fold-changes in signal intensity. The cytokines assessed include CCL2/MCP-1, C5, C5a, CXCL1/GROα, IL-8, MIF, Serpin E1, and PAI-1.

**
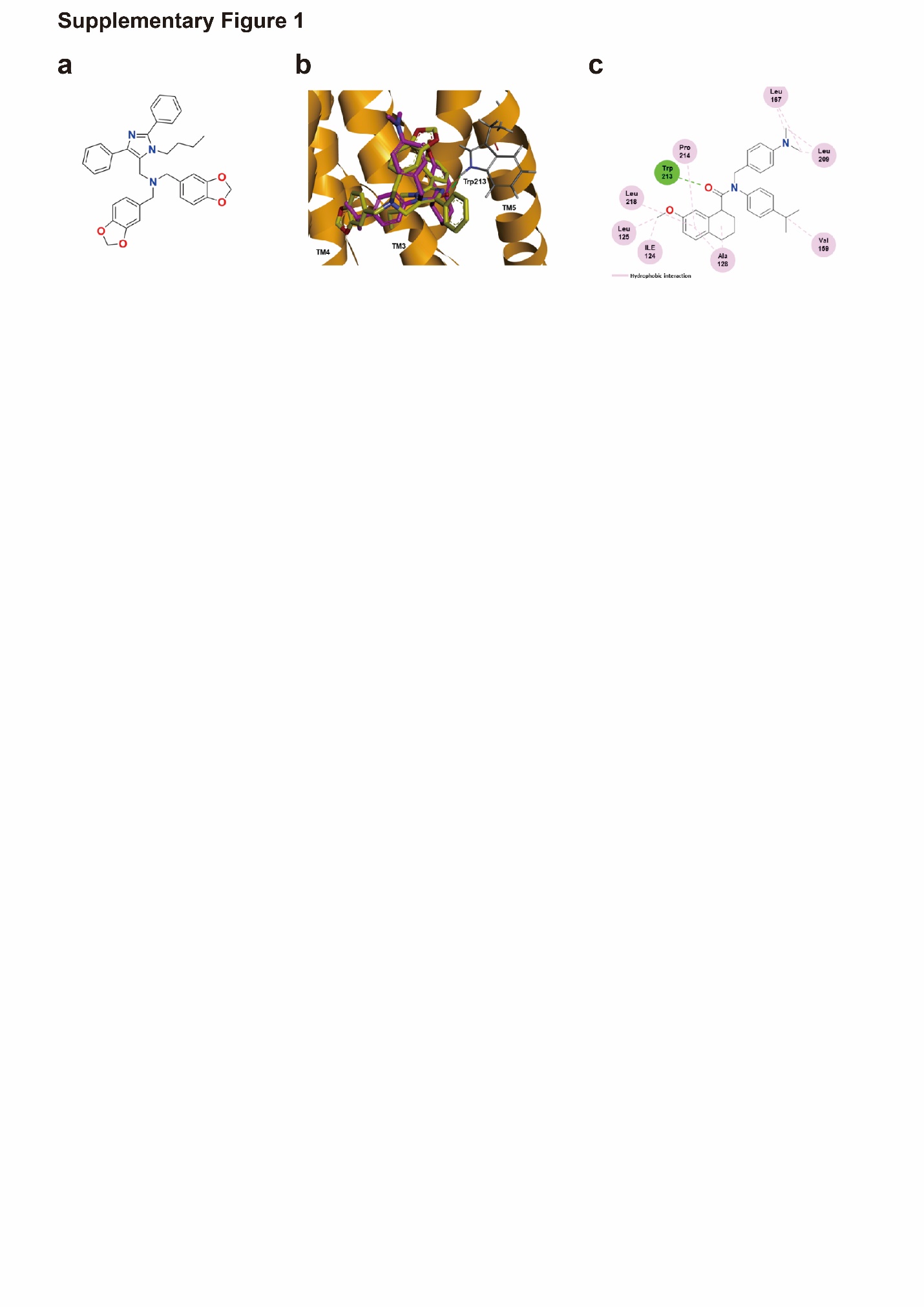
**

**Supplementary Fig. S2. Binding mode of W54011 and NDT9513727. a** Structure of NDT9513727. Predicted binding mode of W54011 with the C5a receptor (C5aR) based on molecular docking. W54011 was synthesized and prepared according to a previously published protocol^7^. The crystal structure of C5aR in complex with the antagonist, NDT9513727 (PDB ID: 5O9H), which binds to an extra-helical site, was used as a structural template due to its high similarity to W54011, including shared amine and alkoxyphenyl moieties. NDT9513727 was removed from the ligand-binding pocket, and W54011 docked into the same site. The resulting model revealed that W54011 fits well within the pocket and maintains a key hydrogen bond with Trp213 via the carbonyl group of its amide moiety—an interaction also observed in the NDT9513727–C5aR complex. See Methods for additional details on the molecular docking procedure. **b** Binding site of W54011 (pink) and NDT9513727 (yellow). **c** Two-dimensional interaction diagram of W54011.

**
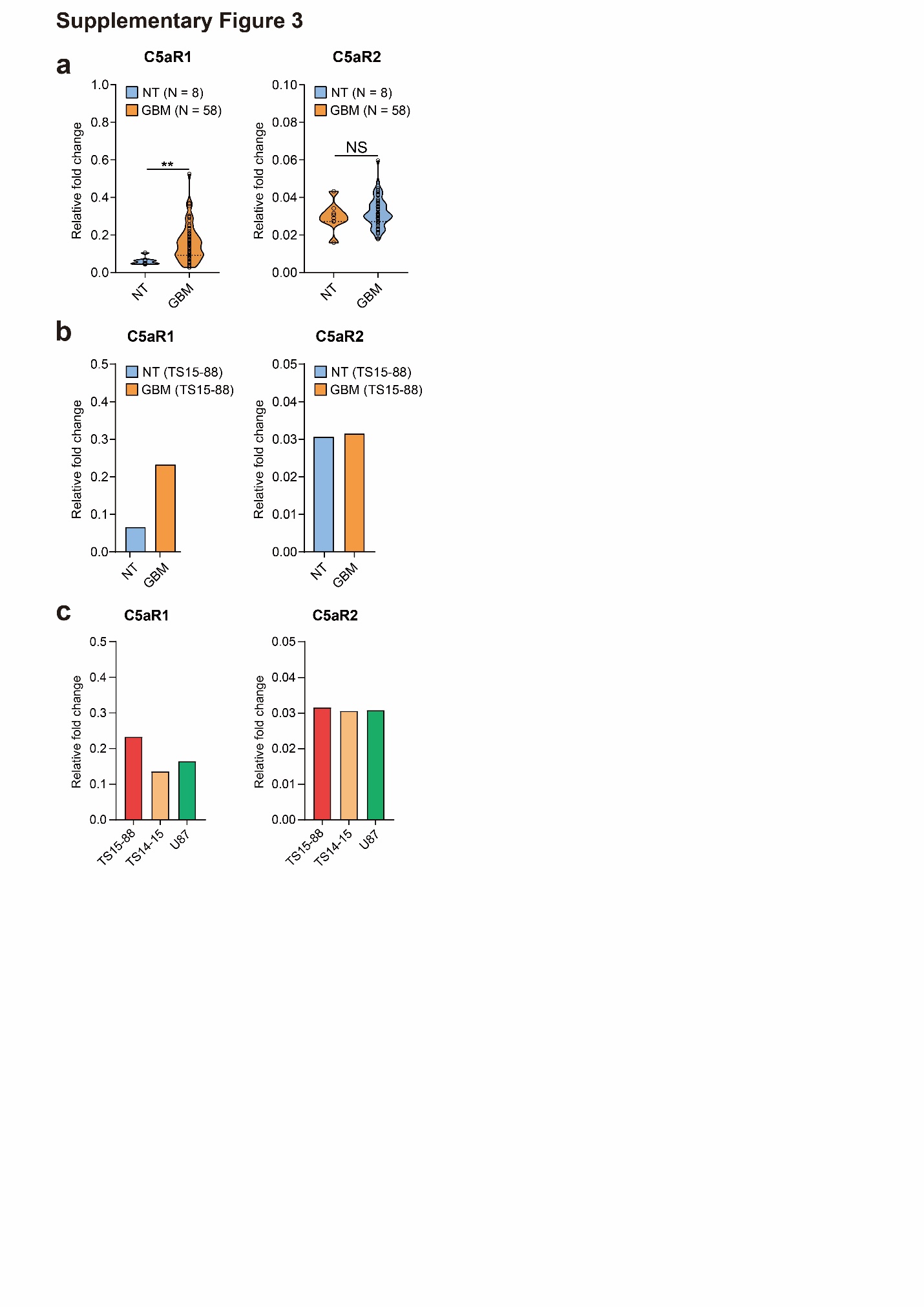
**

**Supplementary Fig. S3. Analysis of C5aR1 and C5aR2 expression profiles in glioblastoma tissue. a** Violin plots showing C5aR1 and C5aR2 expression levels in normal (NT) and glioblastoma (GBM) tissues. Results showed that C5aR1 expression was significantly higher in GBM tissues than in normal tissues (P = 0.0076), whereas C5aR2 levels showed no significant difference (P = 0.5052). **b** Bar graphs illustrating C5aR1 and C5aR2 expression levels in NT and GBM tissues derived from the same patient (TS15-88). The NT tissue was derived from histologically non-tumorous cortical tissue obtained during surgical resection. The label “NT (TS15-88)” is used to reflect its matched origin from the same patient as that of the GBM sample. **c** Bar graphs comparing C5aR1 and C5aR2 expression levels in TS15-88, TS14-15, and U87. Grid lines represent the median, whereas dotted lines indicate the first (bottom) and third (top) quartiles. Statistical significance was calculated using the *t*-test; ^*^P <0.05, ^**^P <0.01, ^***^P <0.001, ^****^P <0.0001, NS = not significant.


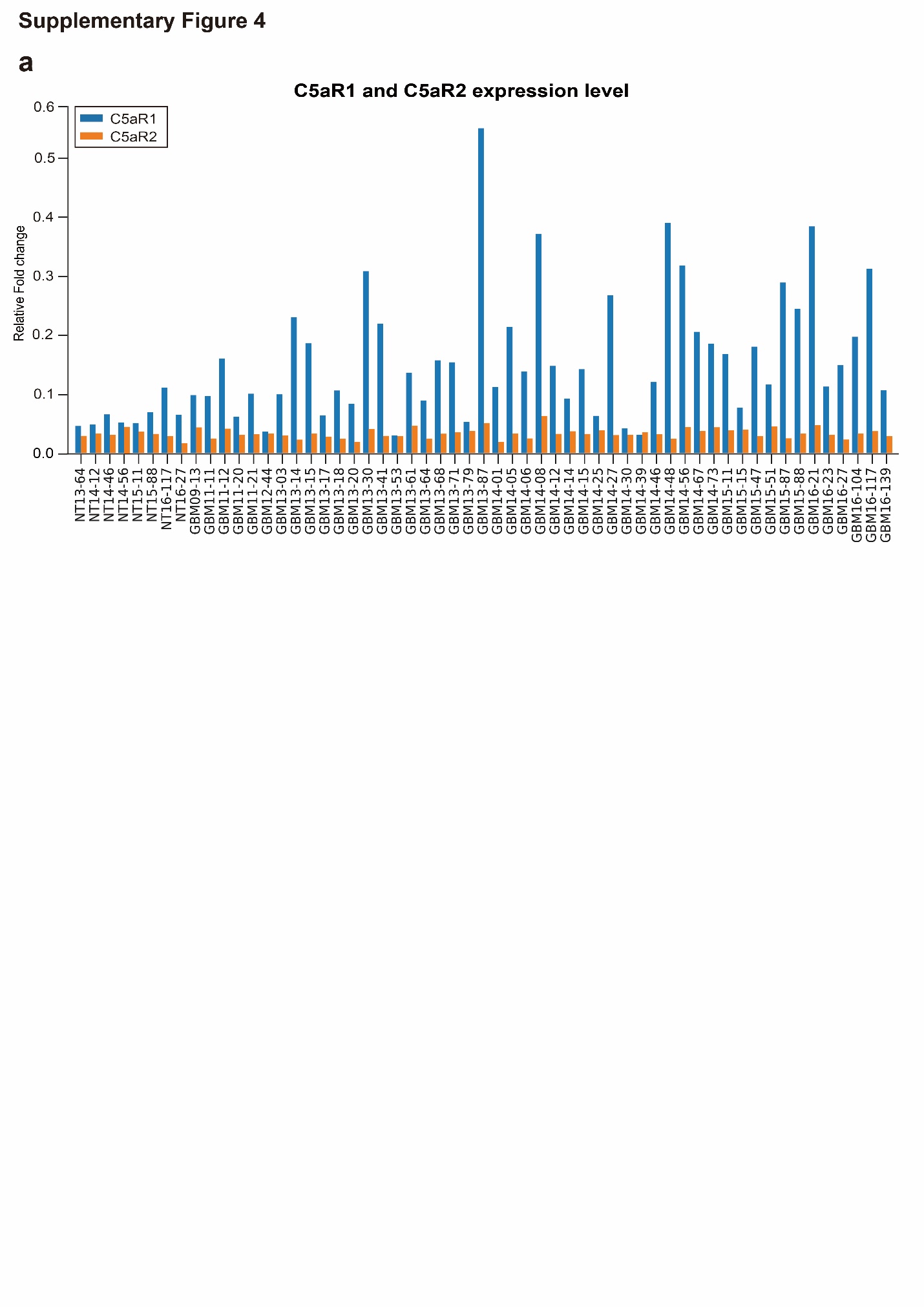


**Supplementary Fig. S4. Expression levels of C5aR1 and C5aR2 in glioblastoma and matched normal tissues.** **a** Relative mRNA expression of C5aR1 (blue) and C5aR2 (orange) via microarray analysis was measured across glioblastoma (GBM) and matched normal tissue (NT) samples. Expression values are shown as relative fold change.

**
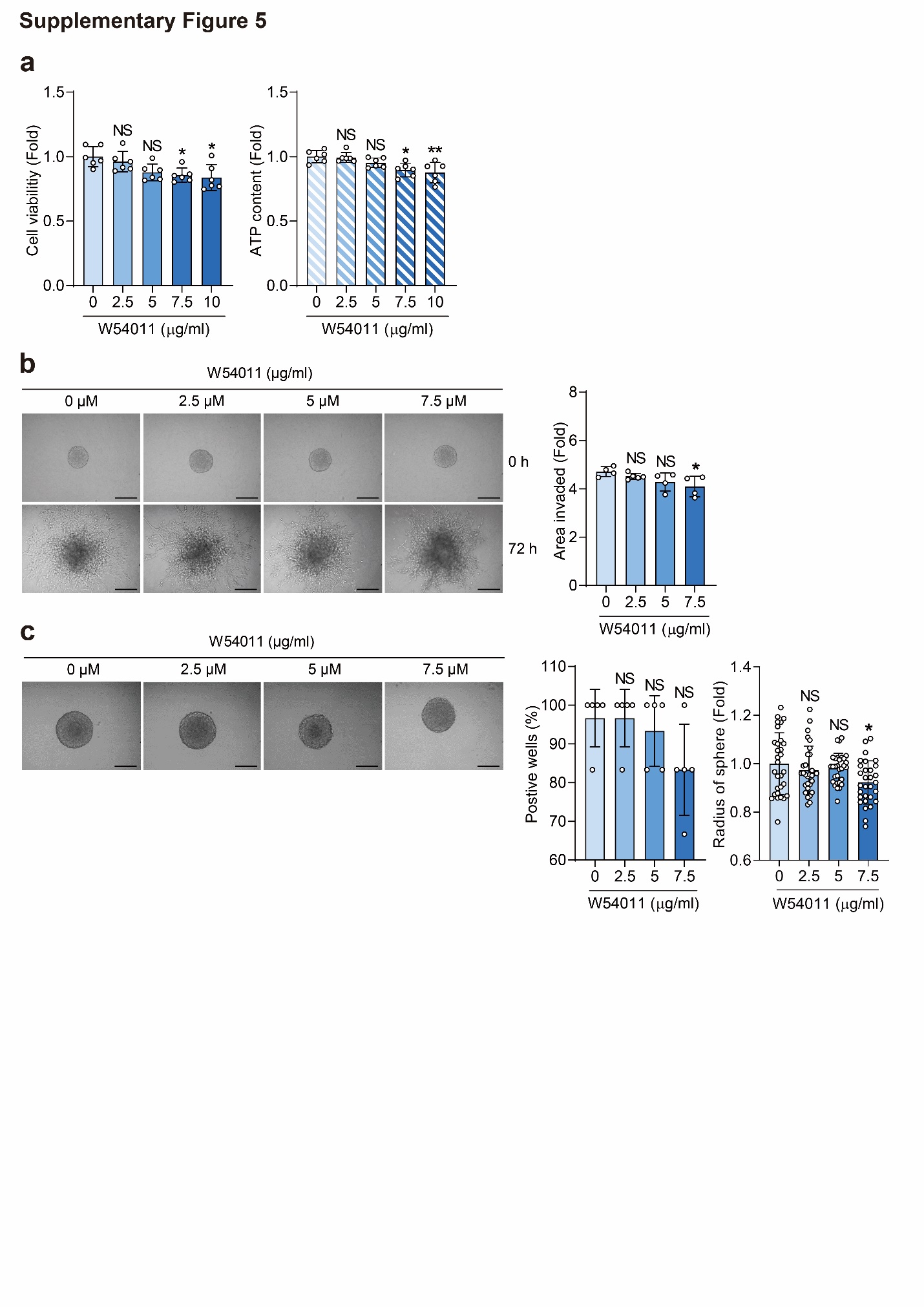
**

**Supplementary Fig. S5. Effects of W54011 without conditioned media on glioblastoma tumorsphere TS15-88 growth, viability, invasion, and stemness. a** Cell viability and ATP content of TS15-88 tumorspheres treated with W54011 (0–10 µg/mL). Viability remained largely unchanged at lower doses but showed a slight reduction at 7.5 µg/mL, indicating mild toxicity. **b** Representative images of invasion assays following W54011 treatment (0, 2.5, 5, and 7.5 µg/mL), with quantification of the invaded area. Scale bars, 200 µm. **c** Representative images of neurosphere formation assays with quantification of the positive wells and sphere radius. Scale bars, 200 µm. Statistical significance was evaluated using one-way analysis of variance, followed by Tukey’s post hoc test. Results are presented as means ± standard deviation, with significance levels denoted as *P <0.05, **P <0.01, and ***P <0.001; NS = not significant.


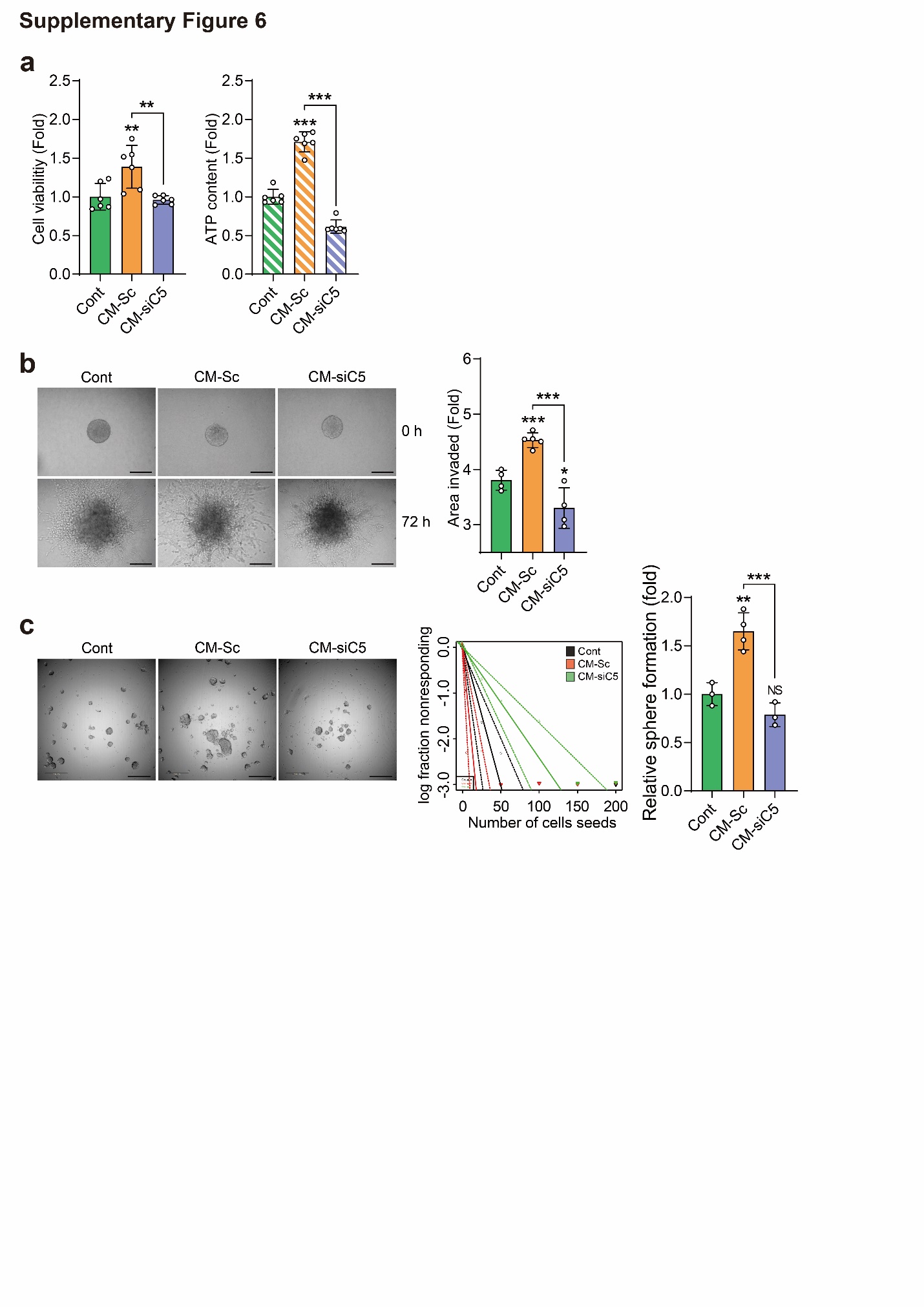


**Supplementary Fig. S6. C5 silencing in tumor mesenchymal stem-like cell-conditioned medium abrogates proliferation, invasion, and stemness of glioblastoma tumorspheres (TS15-88). a** Cell viability and ATP content of TS15-88 cultured in standard tumorsphere medium (Cont), conditioned medium from scrambled siRNA–transfected tumor mesenchymal stem-like cells (tMSLCs) (CM-Sc), or conditioned medium from C5 siRNA–transfected tMSLCs (CM-siC5). CM-Sc increased both parameters, whereas CM-siC5 abolished this effect. **b** Matrigel invasion assays at baseline (day 0) and after 48 h. CM-Sc promoted invasive outgrowth, whereas CM-siC5 suppressed invasion relative to Cont. Scale bars, 200 μm. **c** Limiting-dilution sphere-formation assays. CM-Sc enhanced sphere frequency and size, whereas CM-siC5 failed to augment self-renewal. Representative images (left), extreme limiting dilution analysis (middle), and quantification of relative sphere formation (right) are shown. Scale bars, 500 μm. Statistical significance was evaluated using one-way analysis of variance, followed by Tukey’s post hoc test. Results are presented as means ± standard deviation, with significance levels denoted as *P <0.05, **P <0.01, and ***P <0.001; NS = not significant.


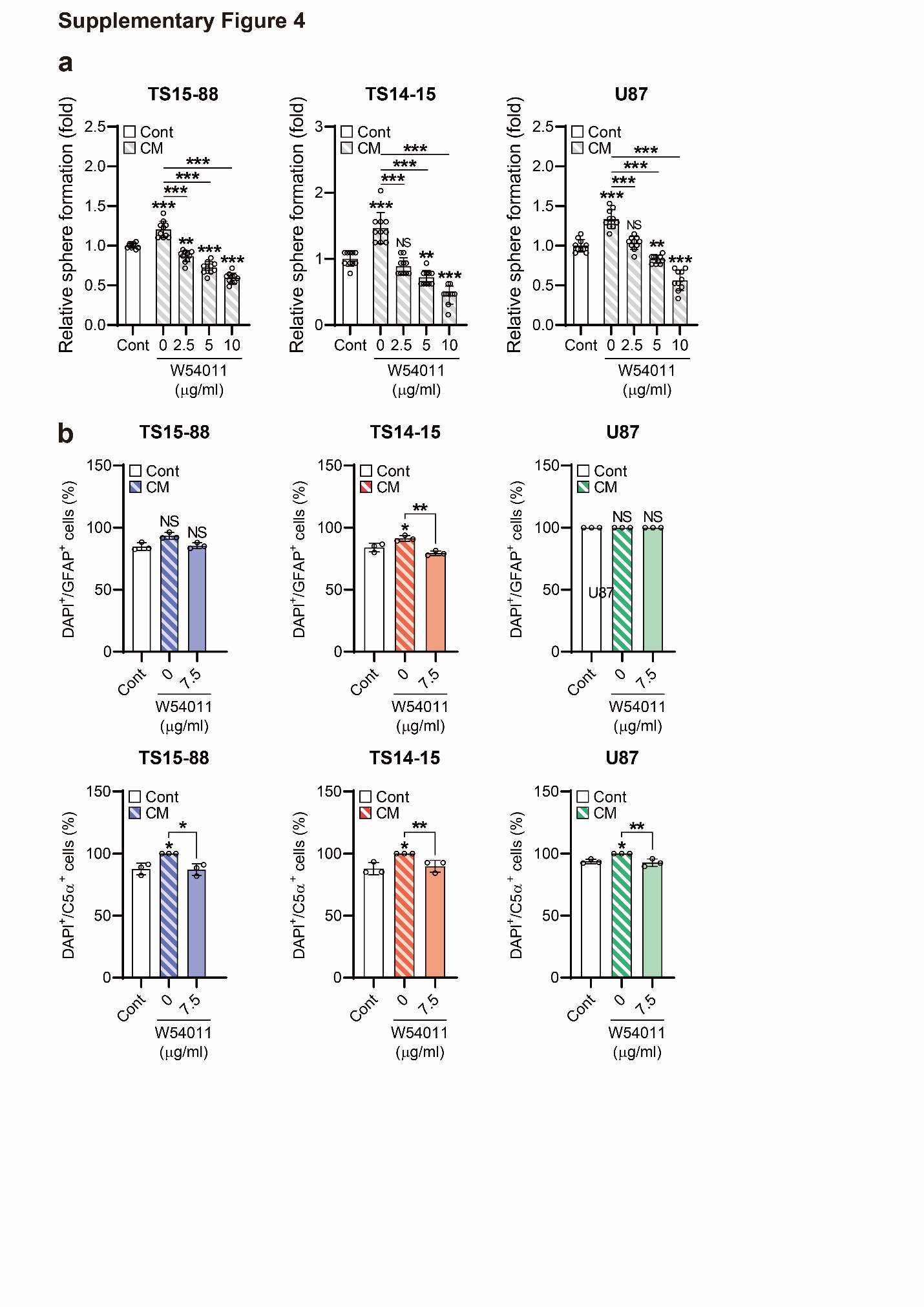


**Supplementary Fig. S7. Quantification of assays shown in Fig. 3a and Fig. 3e. a** Extreme limiting dilution assays in TS15-88, TS14-15, and U87 tumorspheres cultured under control conditions (Cont), with conditioned medium (CM), or with CM supplemented with W54011 at 2.5, 5, or 10 µg/mL. Sphere numbers per well were quantified after 14 days. Each dot represents one well (n = 10 per condition). **b** Quantification of GFAP^+^ and C5α^+^ cells (as a percentage of DAPI^+^ cells) corresponding to the immunocytochemistry images in **Fig. 3e** in TS15-88, TS14-15, and U87 tumorspheres treated with CM with or without W54011 (7.5 µg/mL). Statistical significance was evaluated using one-way analysis of variance, followed by Tukey’s post hoc test. Results are presented as means ± standard deviation, with significance levels denoted as *P <0.05, **P <0.01, and ***P <0.001; NS = not significant.

**
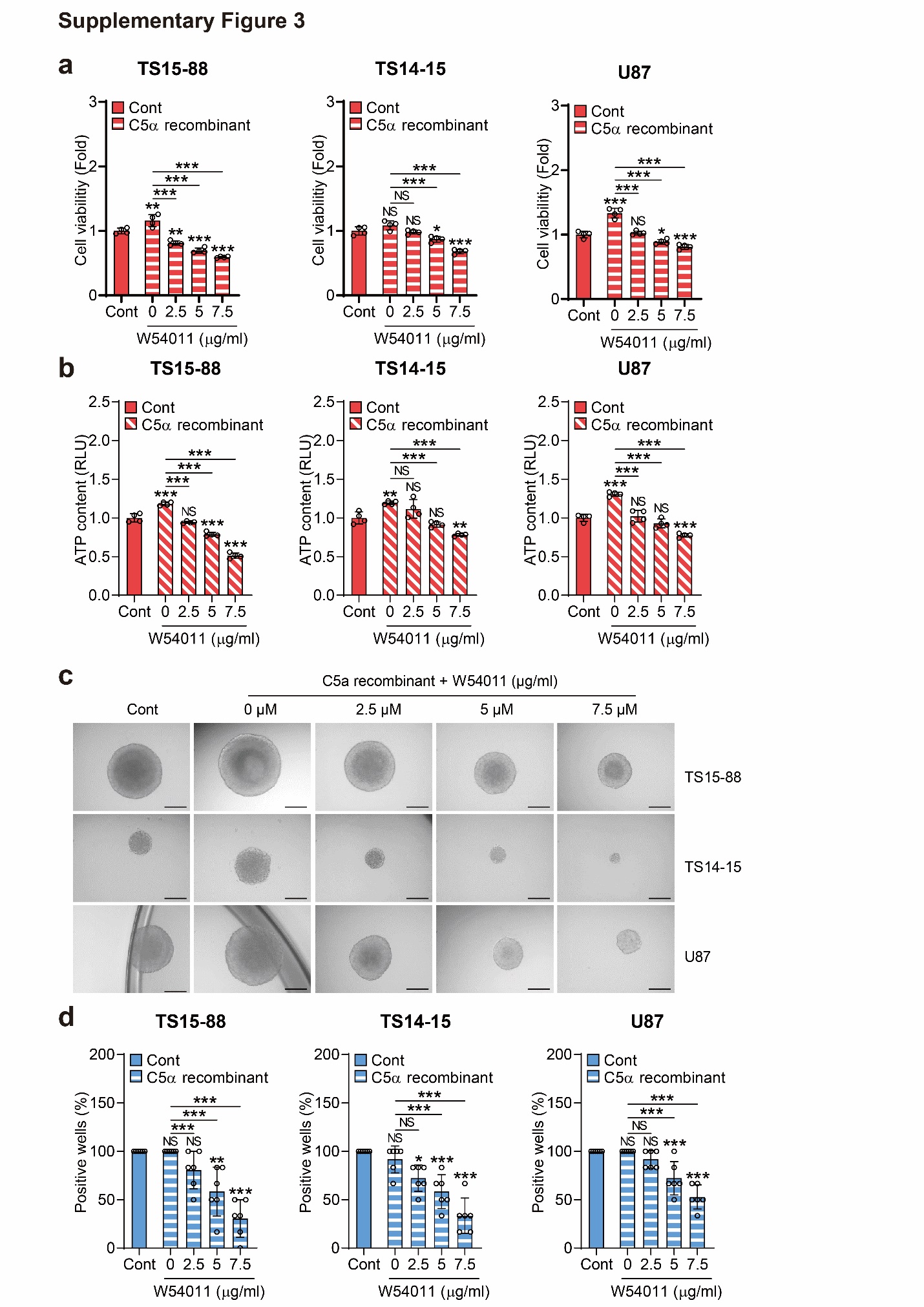
**

**
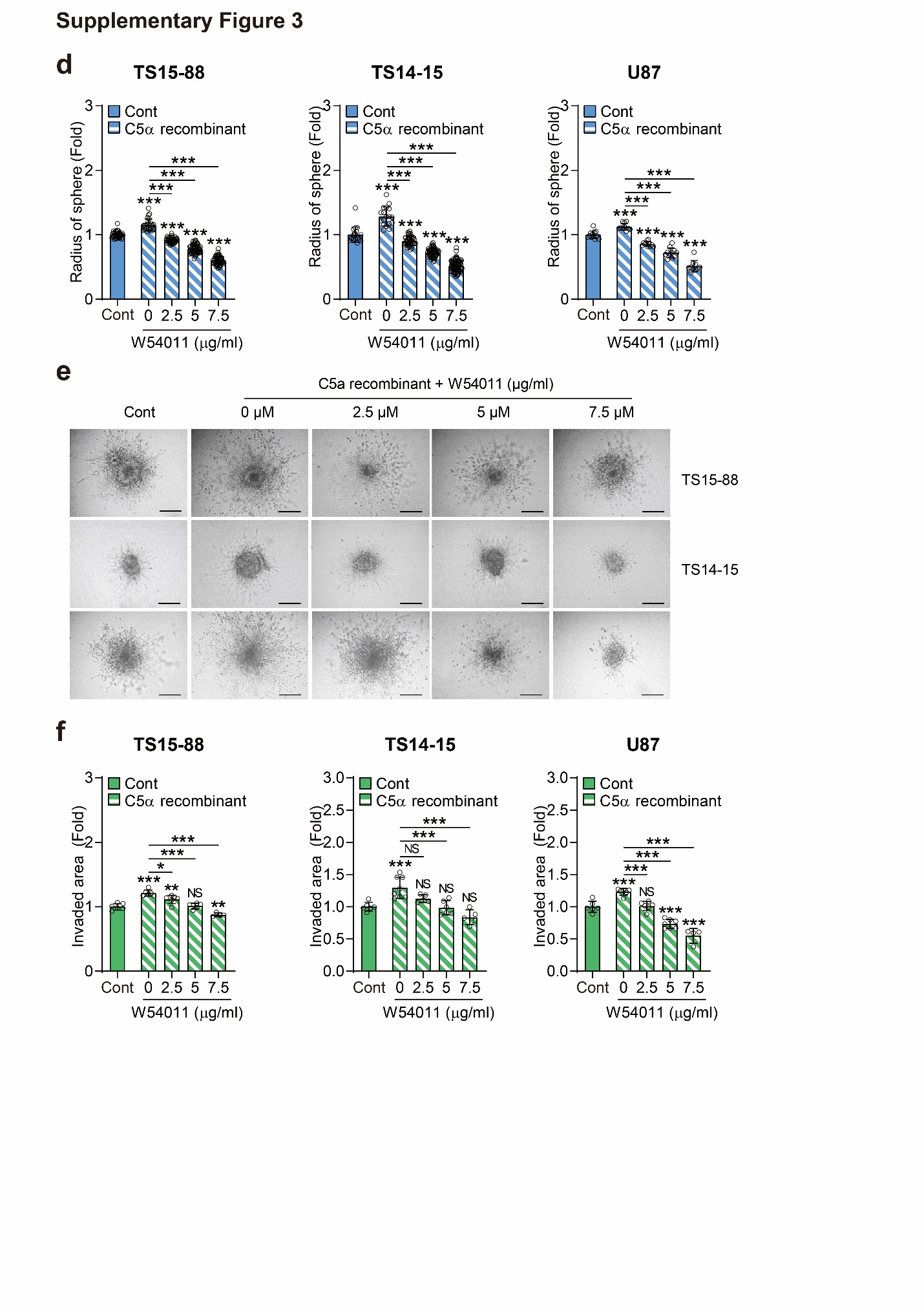
**

**Supplementary Fig. S8. Recombinant C5a and W54011 treatments inhibit glioblastoma tumorsphere proliferation, neurosphere formation, and invasion.** **a–b** Cell viability (**a**) and ATP content (**b**) in three glioblastoma (GBM) tumorspheres (TS15-88, TS14-15, and U87) treated with recombinant C5a (50 nM) alone and in combination with W54011 at varying concentrations (0–7.5 μM). **c** Brightfield micrographs of the GBM tumorspheres treated with recombinant C5a (50 nM) alone and in combination with W54011 (0–7.5 μM) at an initial seeding density of 1.0 × 10^4^ cells. Scale bars = 200 μm. **d** Neurosphere formation assay assessing stemness after treatment with recombinant C5a (50 nM) and W54011 (0–7.5 μM) for three weeks. Images were captured at 100× magnification. **e** Quantification of positive wells with neurosphere formation and sphere radii. **e** Invasiveness of the GBM tumorspheres lines assessed by embedding the cells in 3D matrix gels. Scale bar = 200 μm. **f** Bar graphs quantifying the invaded areas. Statistical significance was evaluated using one-way analysis of variance, followed by Tukey’s post hoc test. Results are presented as means ± standard deviation, with significance levels denoted as *P <0.05, **P <0.01, and ***P <0.001; NS = not significant.

**
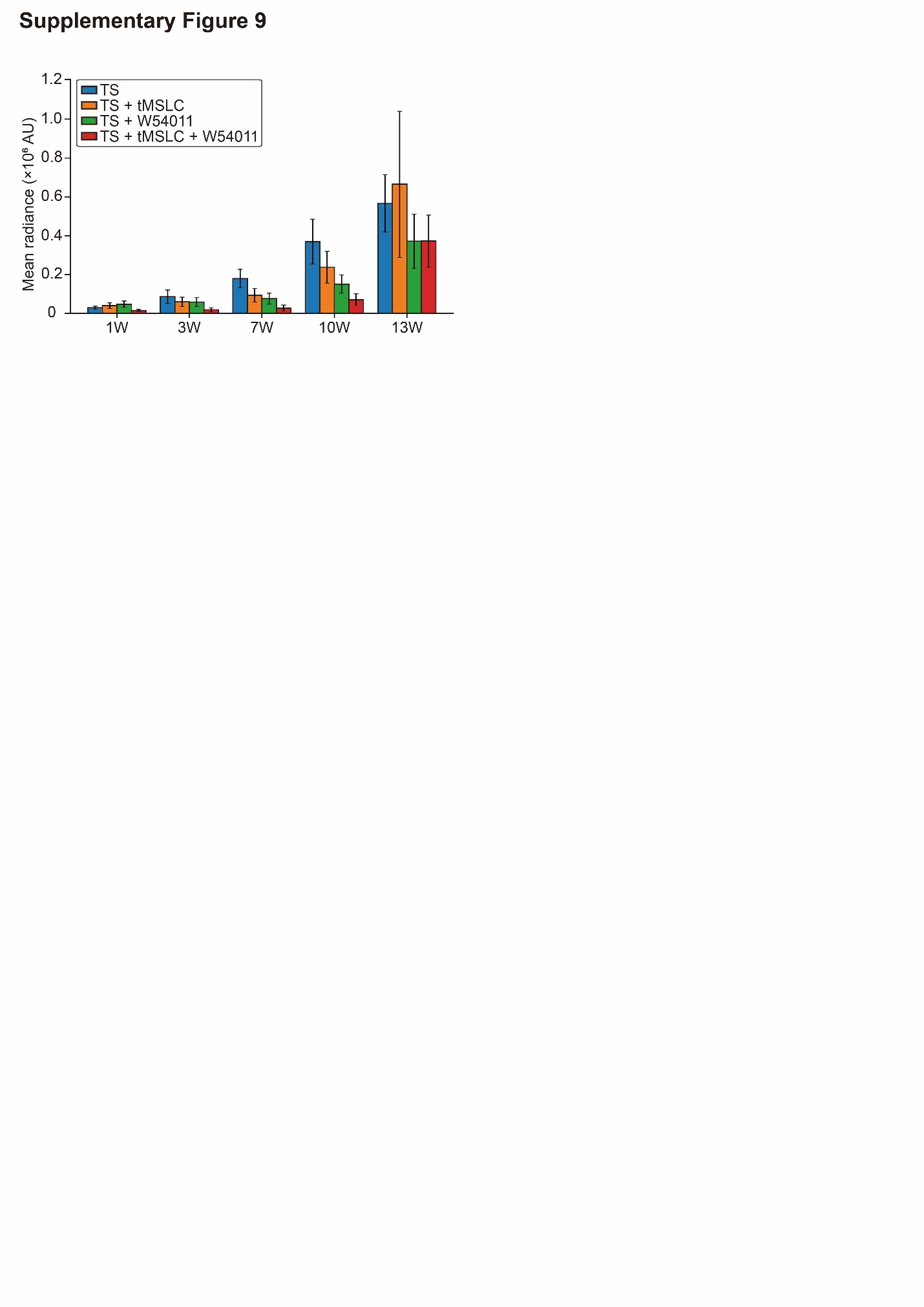
**

**Supplementary Fig. S9. Mean radiance (×10^6^ AU) measured weekly across experimental groups**. Values are shown as mean ± SEM. The plot illustrates temporal changes in tumor radiance and highlights differences related to tumor mesenchymal stem-like cell (tMSLC) and W54011 treatment; however, none of the differences reached statistical significance. TS = tumorsphere.

**
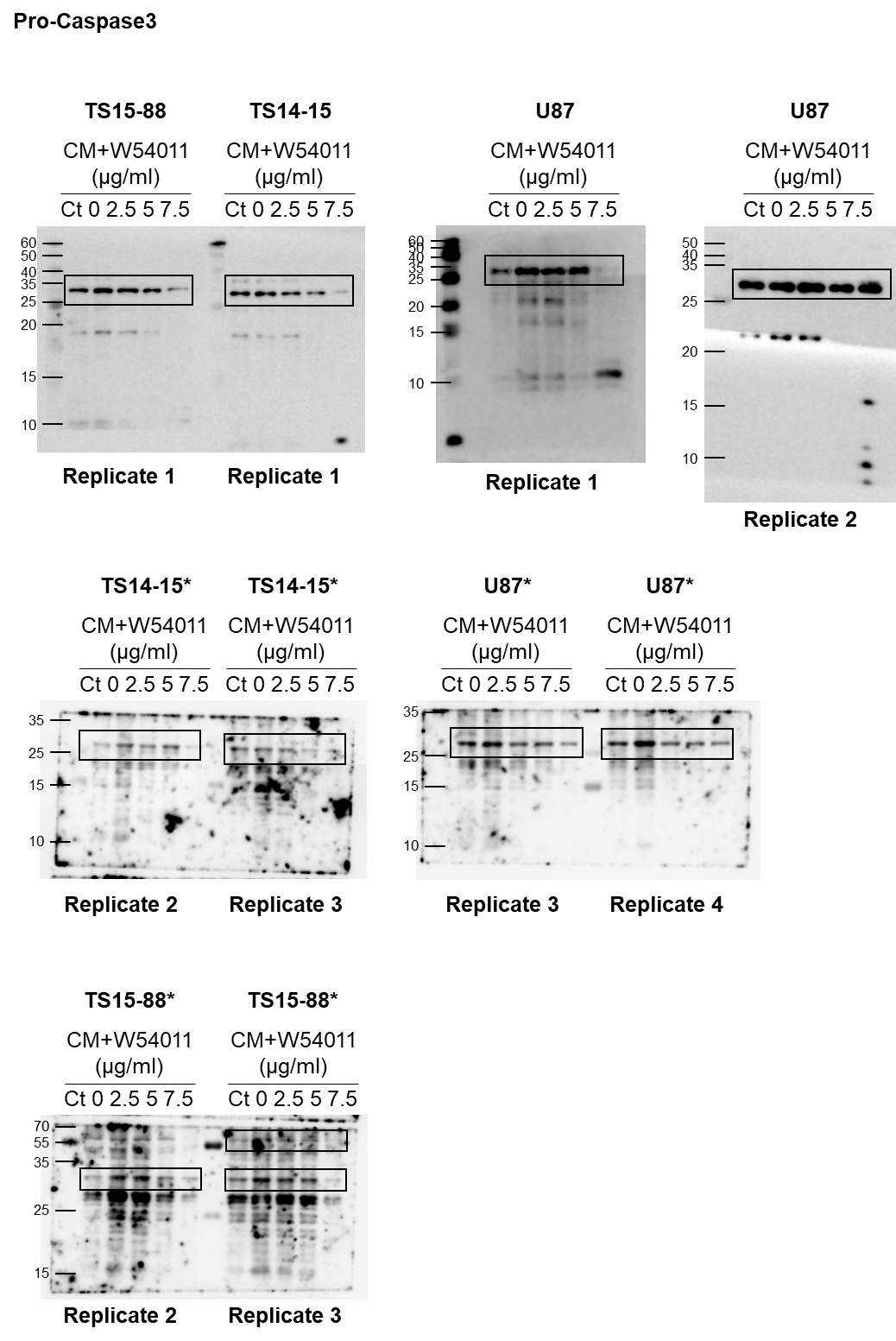
**

**
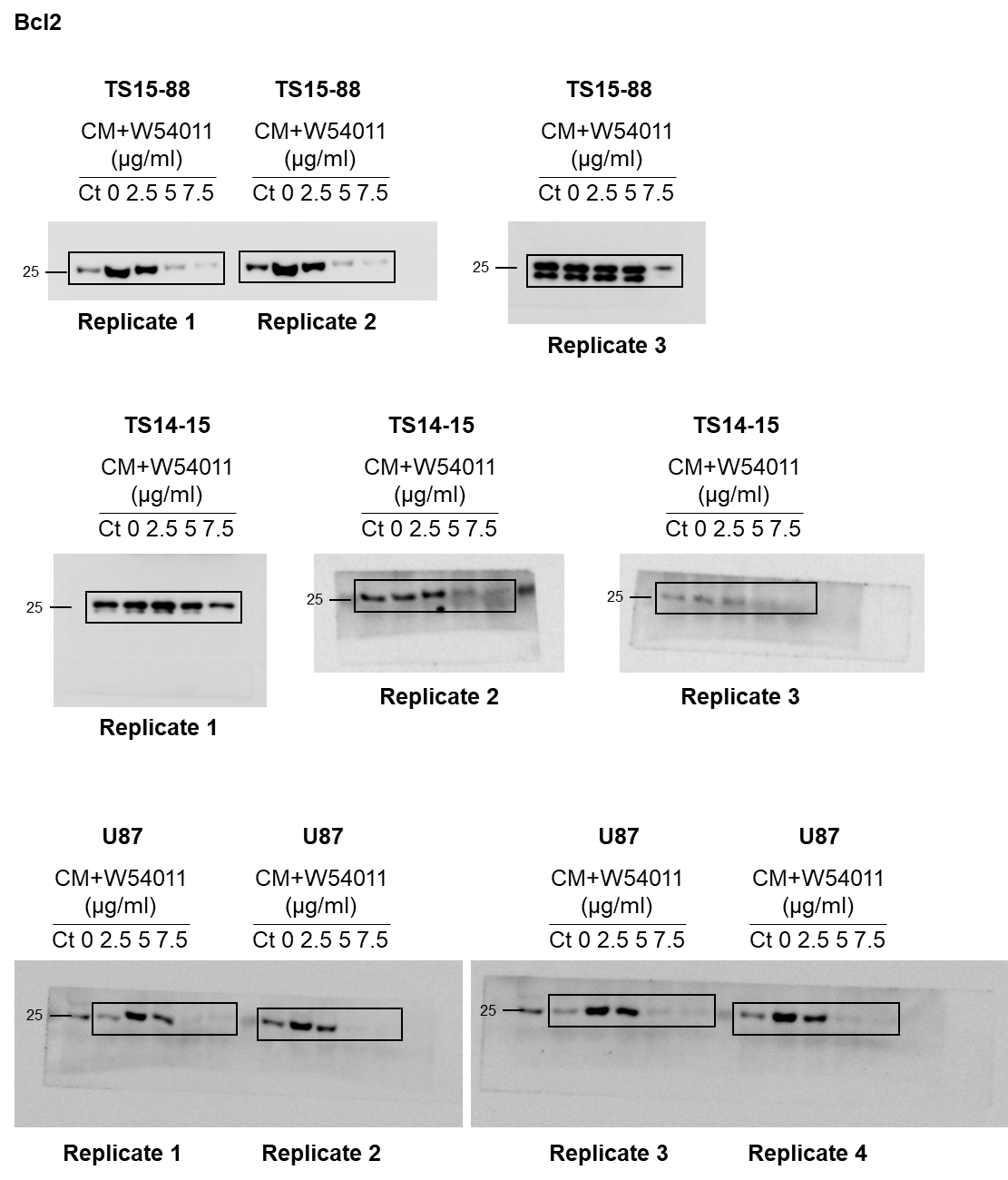
**

**
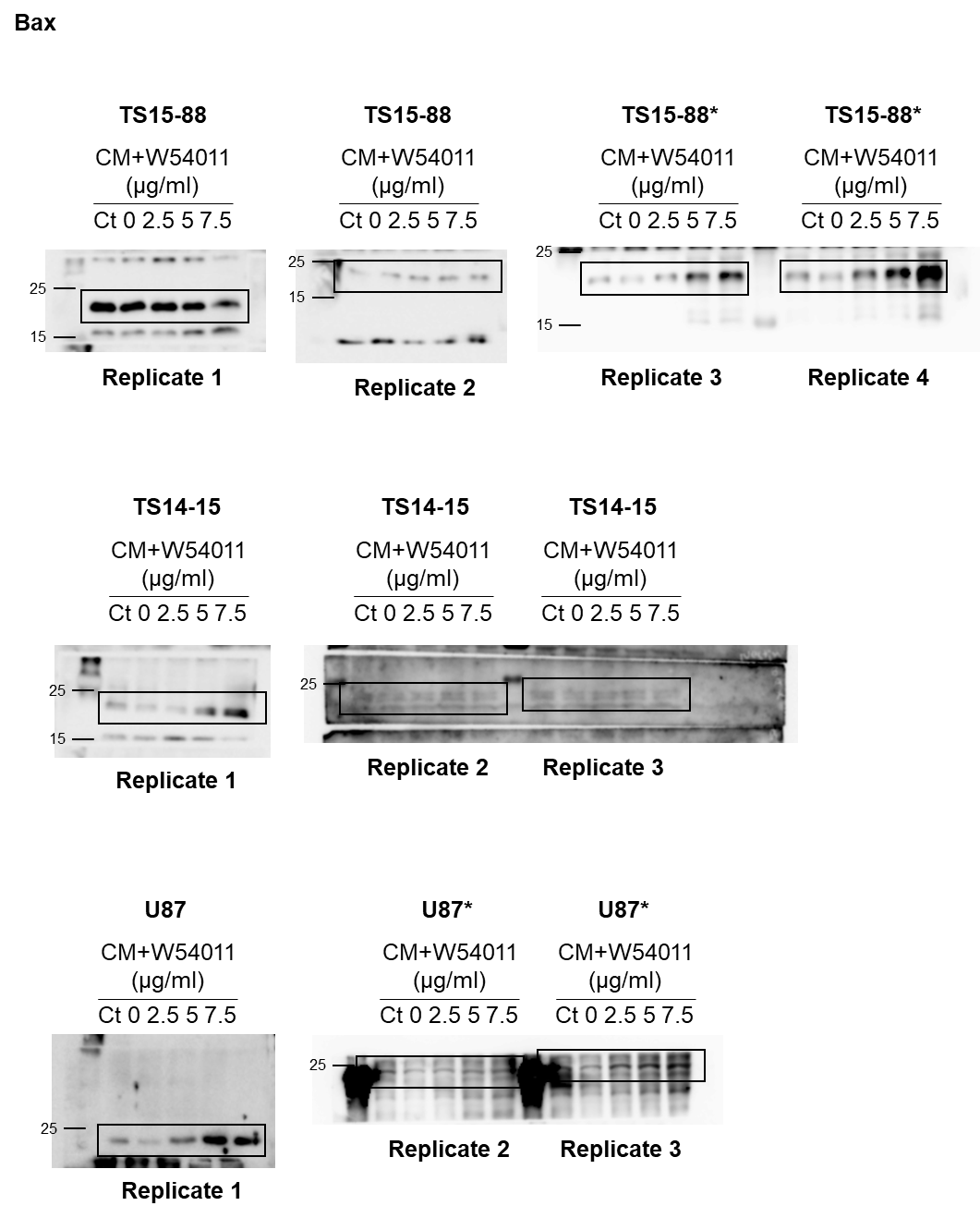
**

**
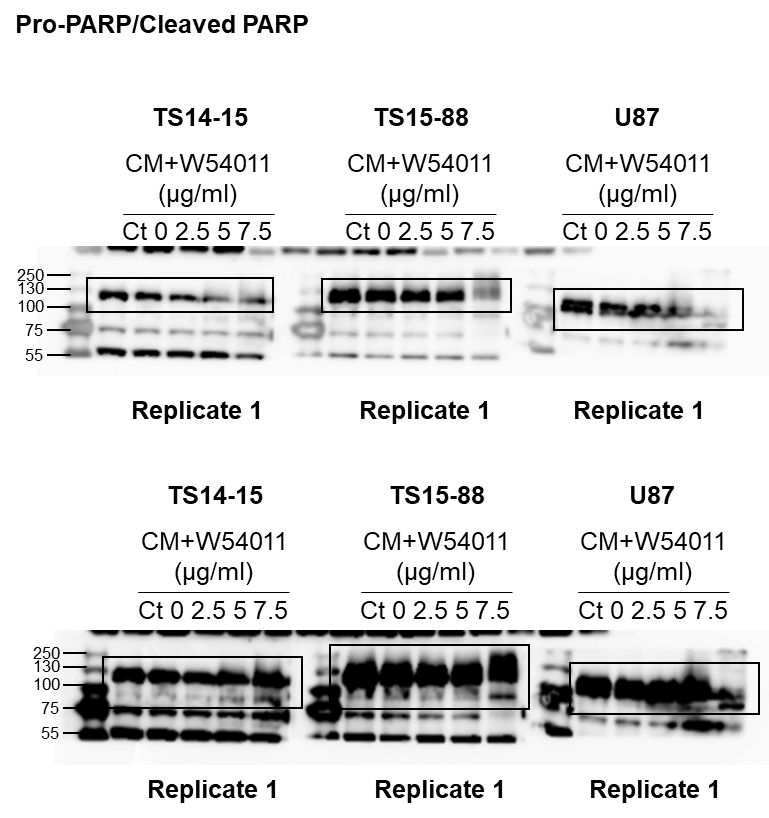
**

**
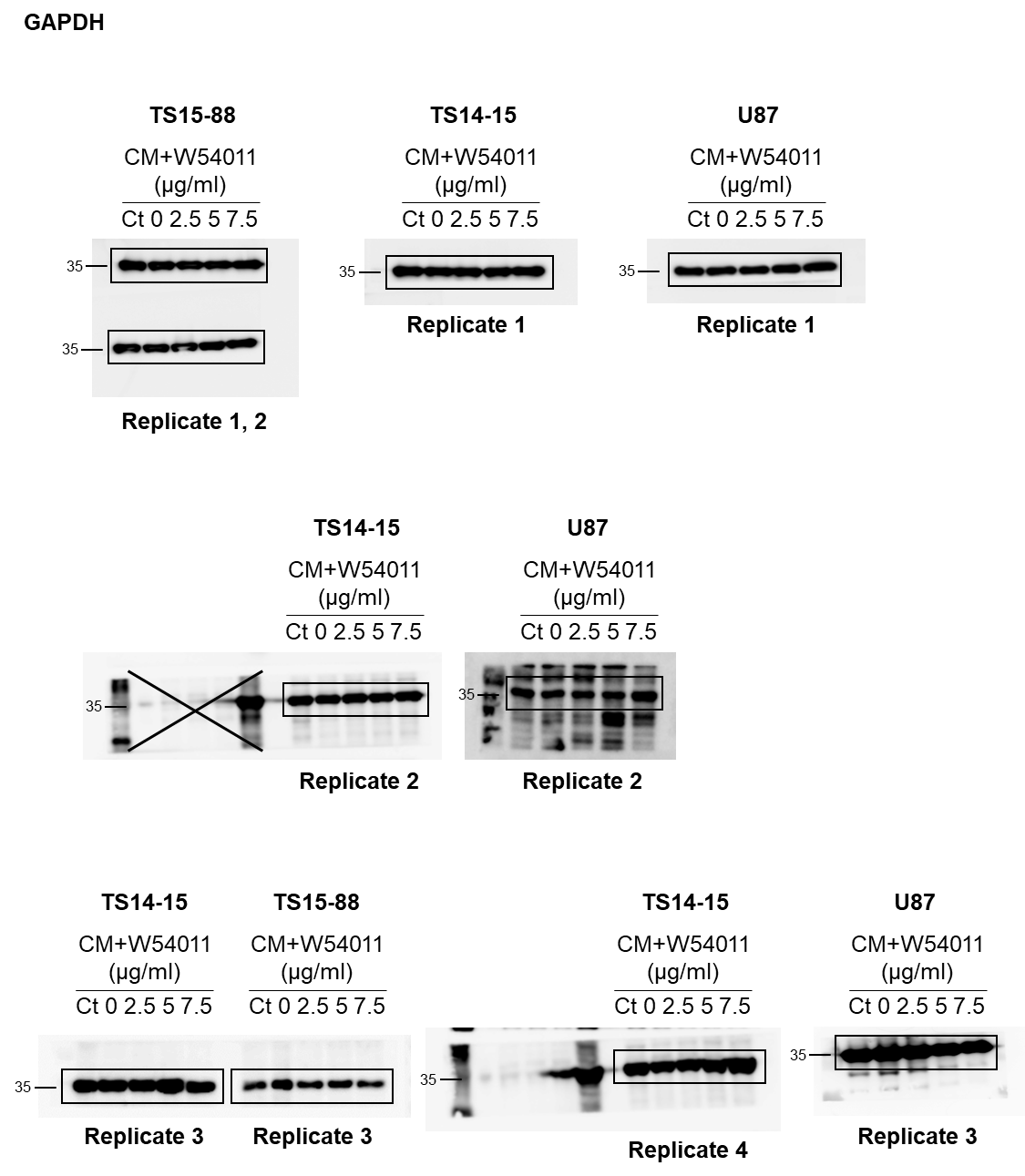
**

**
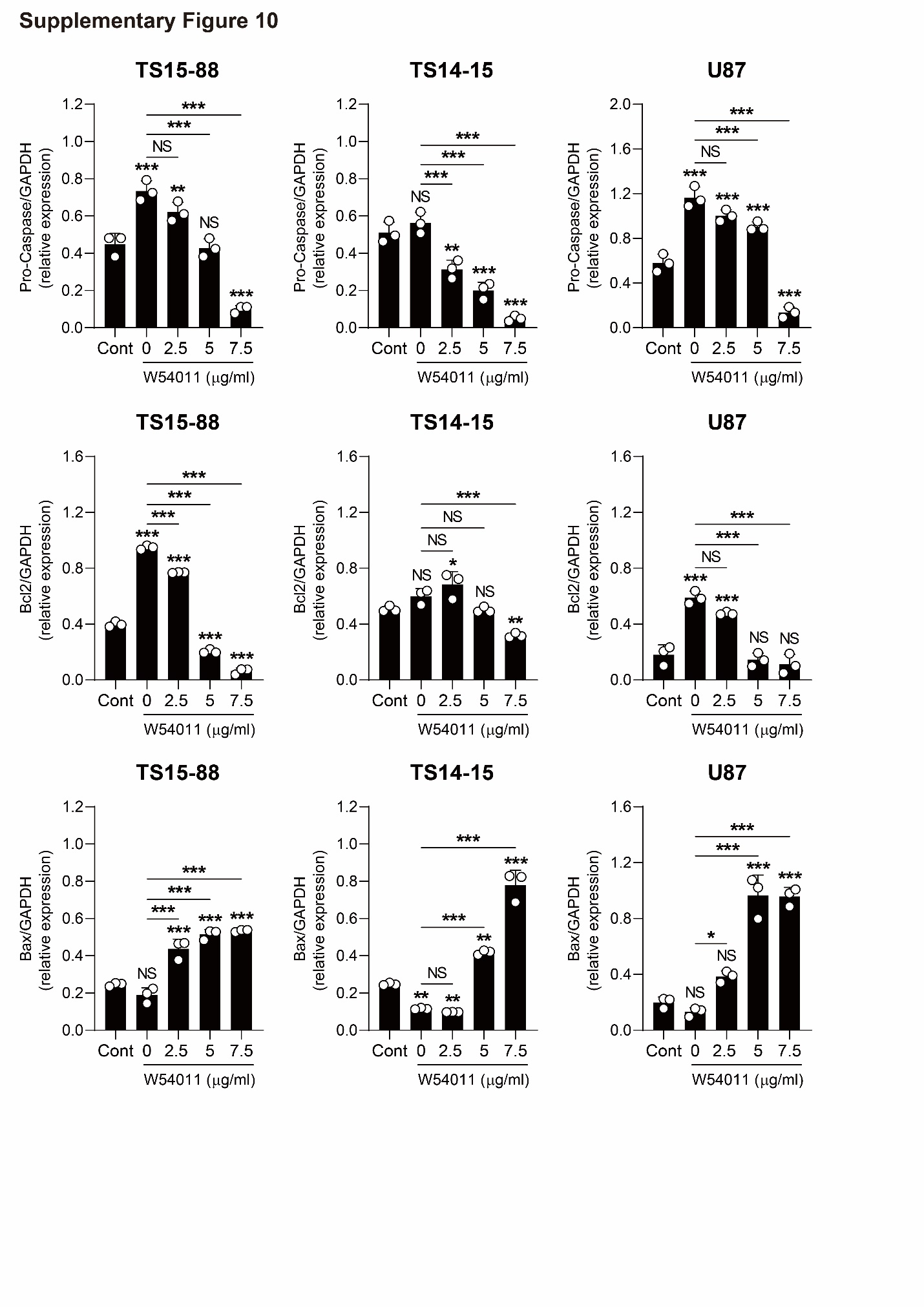
**

**
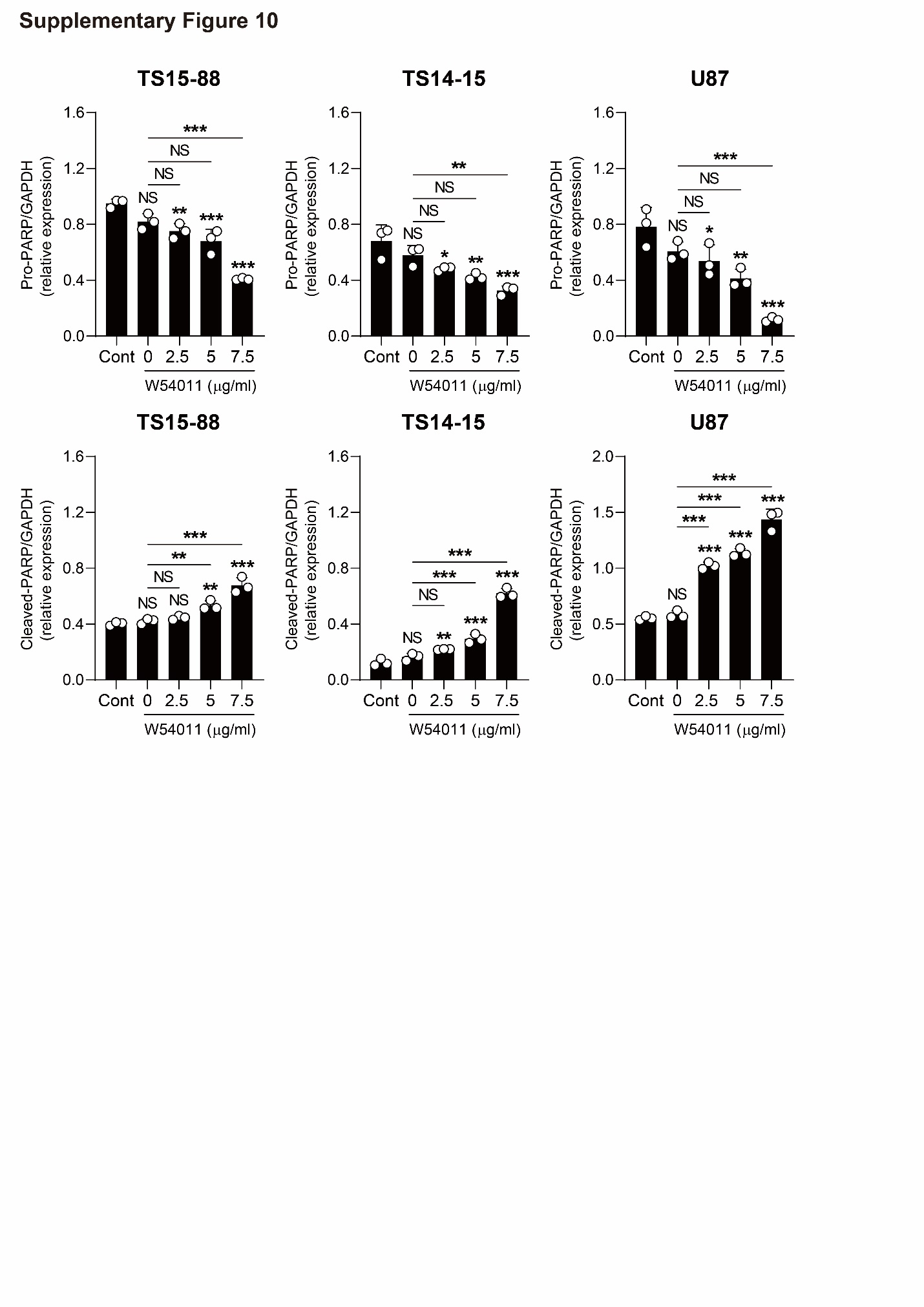
**

**Supplementary Fig. S10.** Raw western blot images corresponding to **Fig. 2d**. Patient-derived tumorspheres (TS15-88, TS14-15) and U87 cells were treated with tMSLC-derived conditioned medium (CM) in the presence of increasing concentrations of W54011 (0, 2.5, 5, and 7.5 µg/mL). Densitometric analyses of these blots are presented as bar graphs. Statistical significance was determined by one-way ANOVA followed by Tukey’s post hoc test. Data are shown as mean ± s.d., with significance denoted as P < 0.05, P < 0.01, and P < 0.001; NS, not significant.

Blots were sectioned prior to hybridisation to allow sequential probing for multiple targets; accordingly, full-length membranes are not available. Bands marked with an asterisk (*) represent replicate blots performed during the revision process using long-term stored samples.


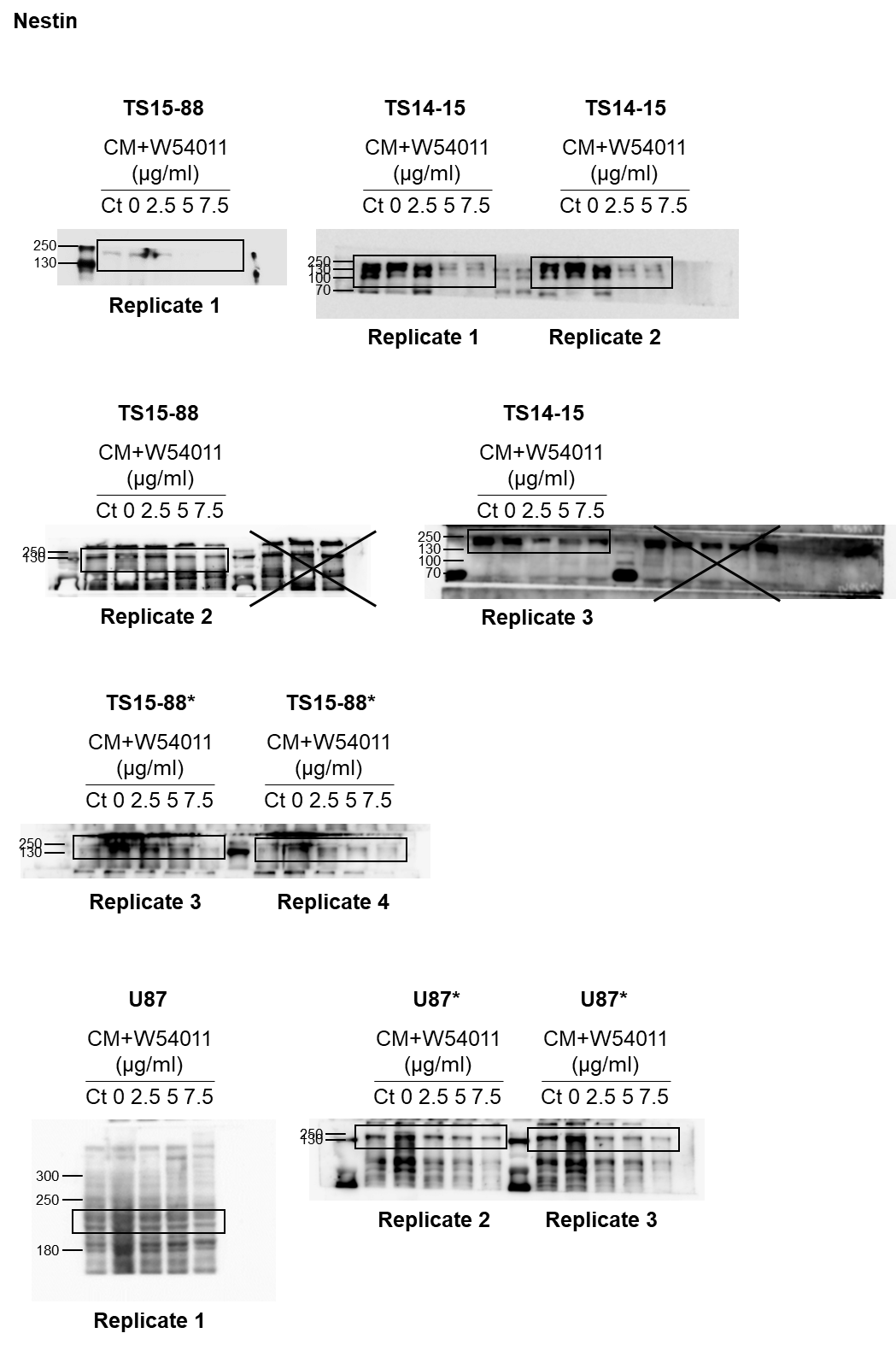


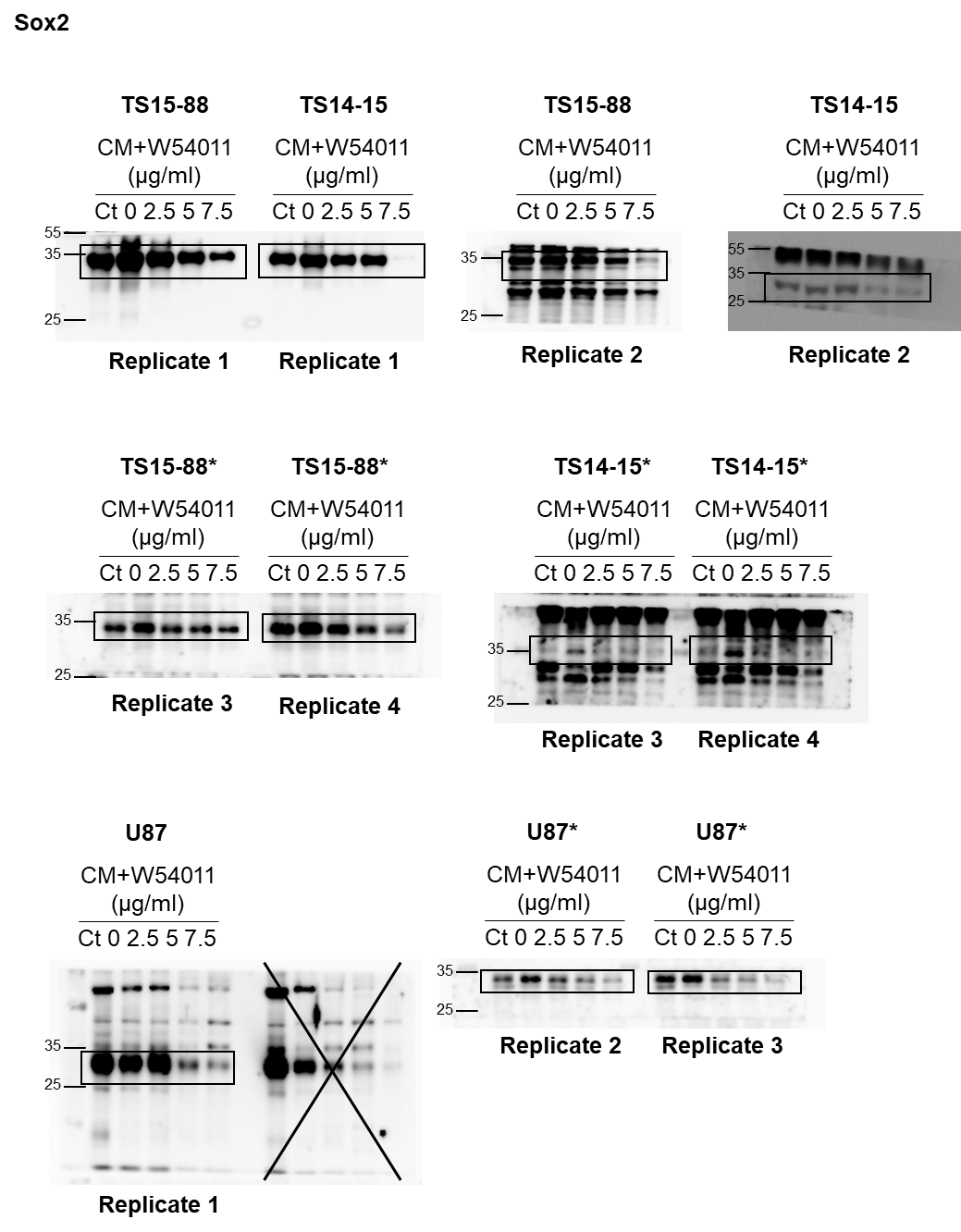


**
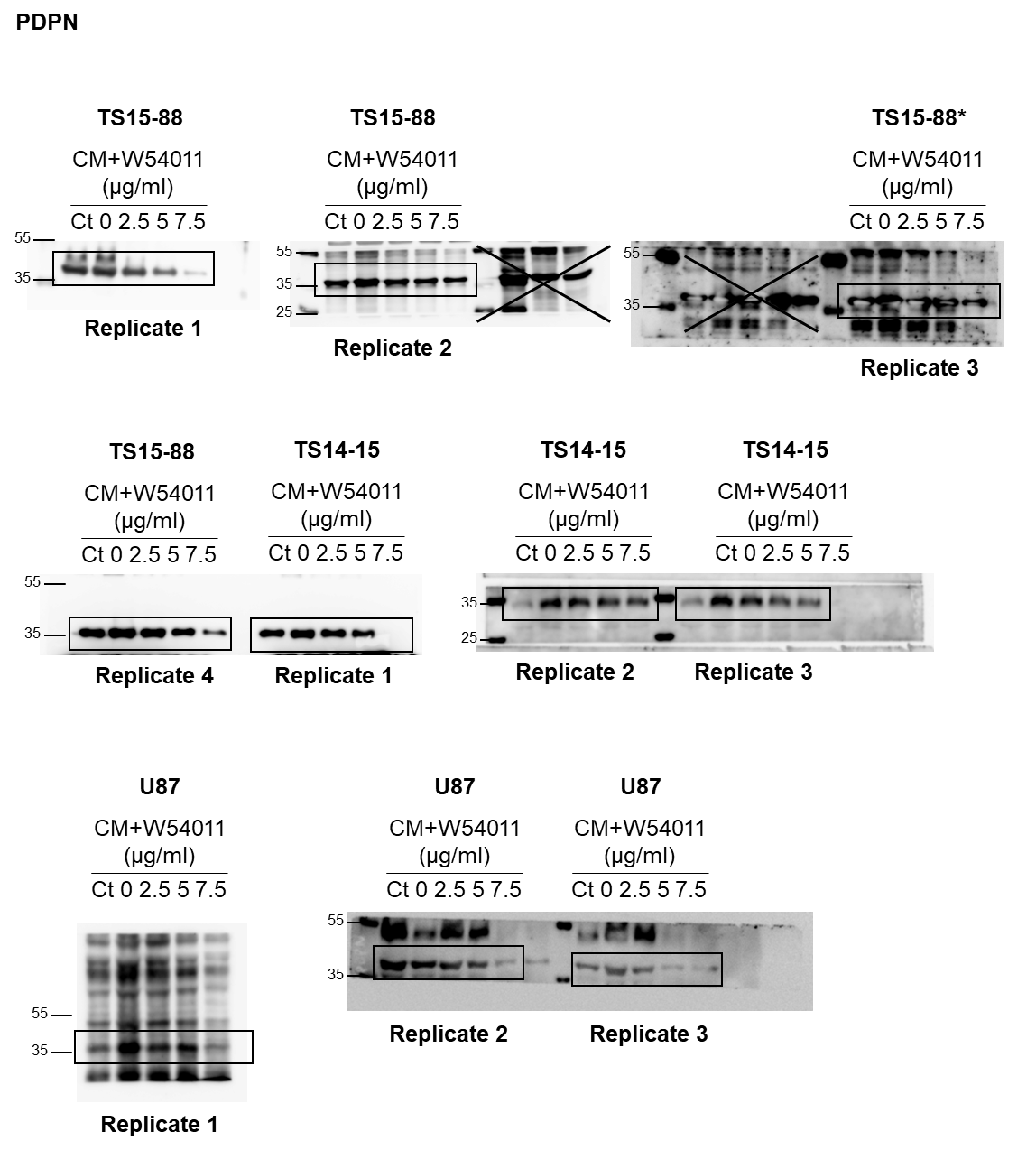
**

**
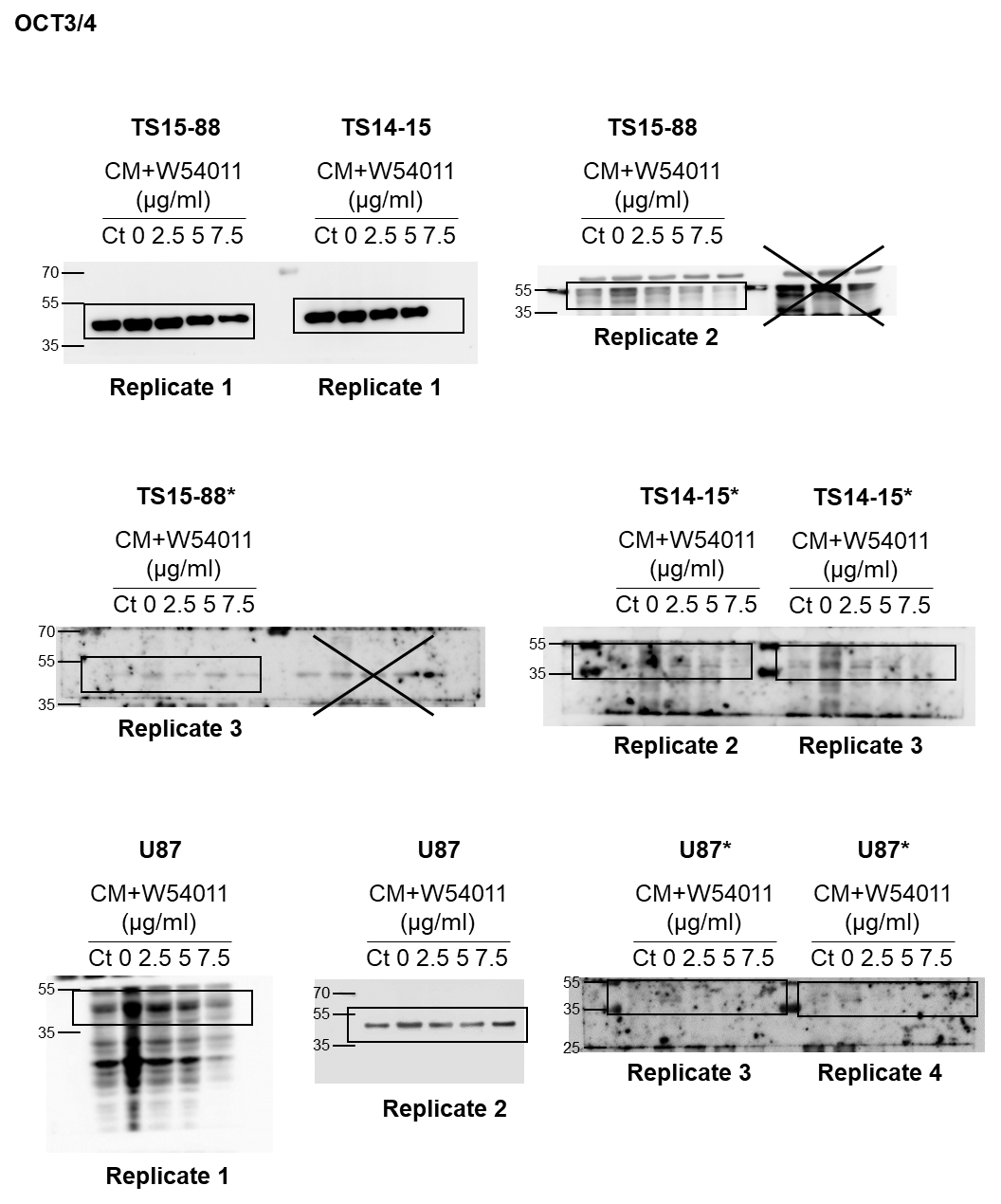
**

**
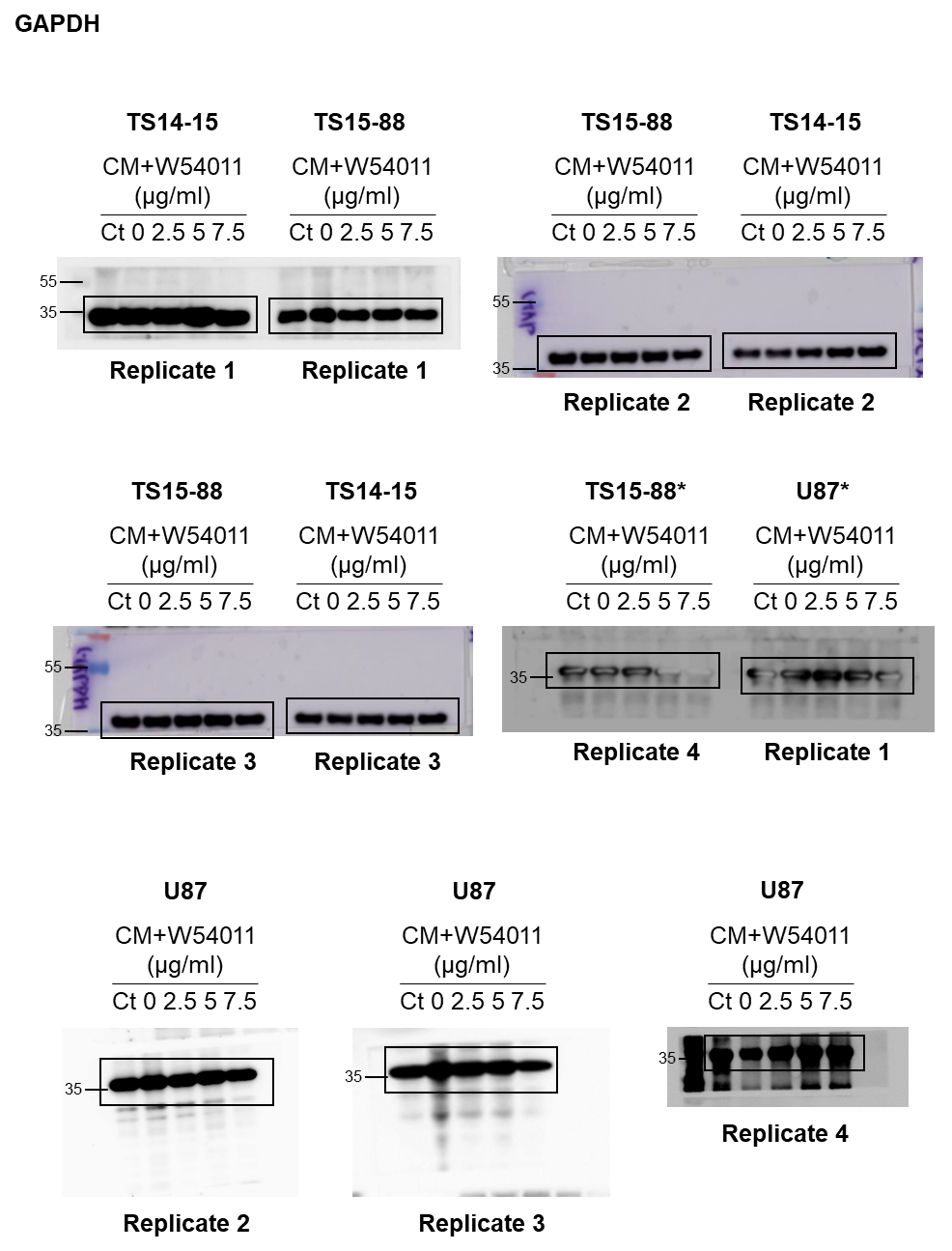
**

**
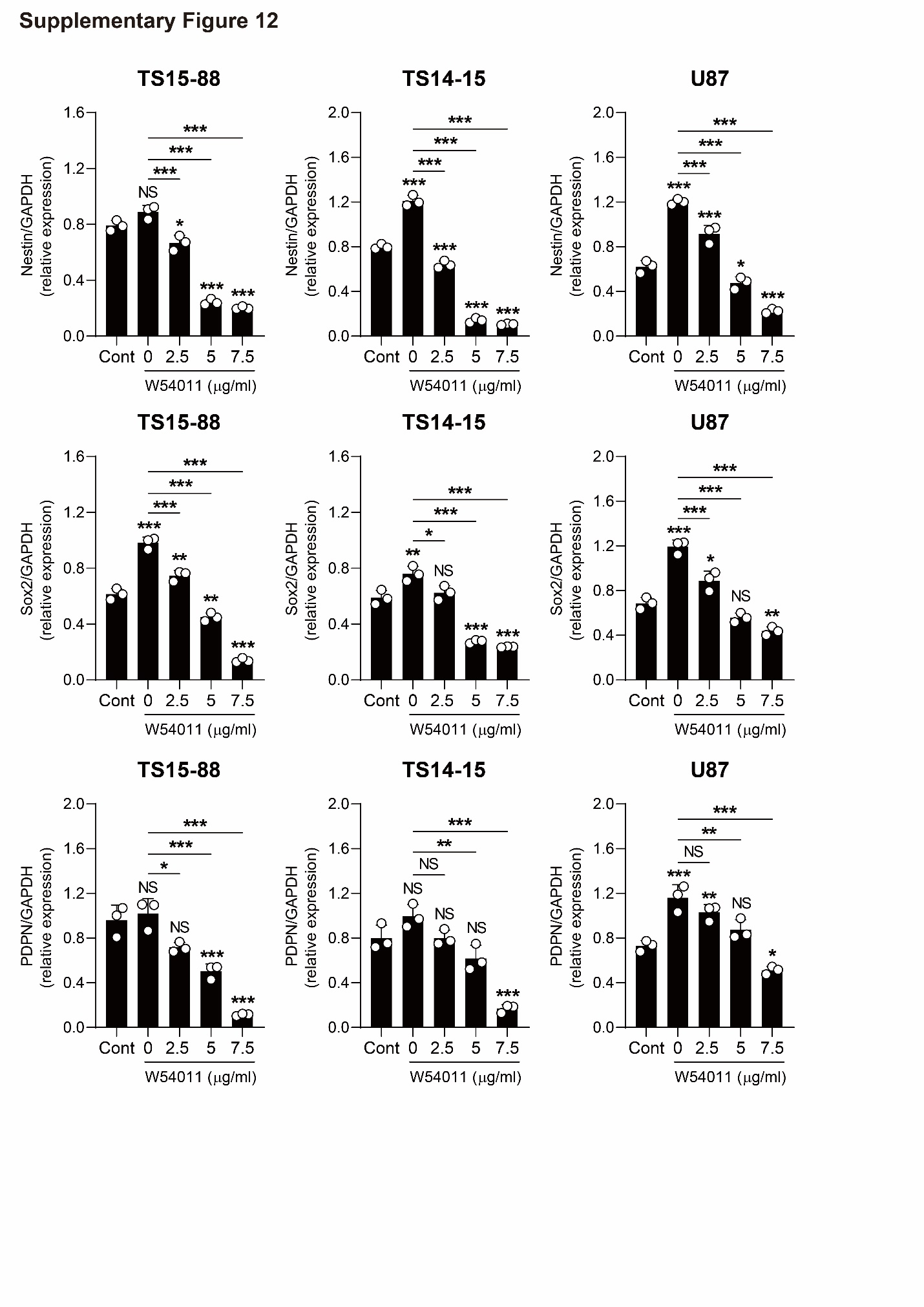
**

**
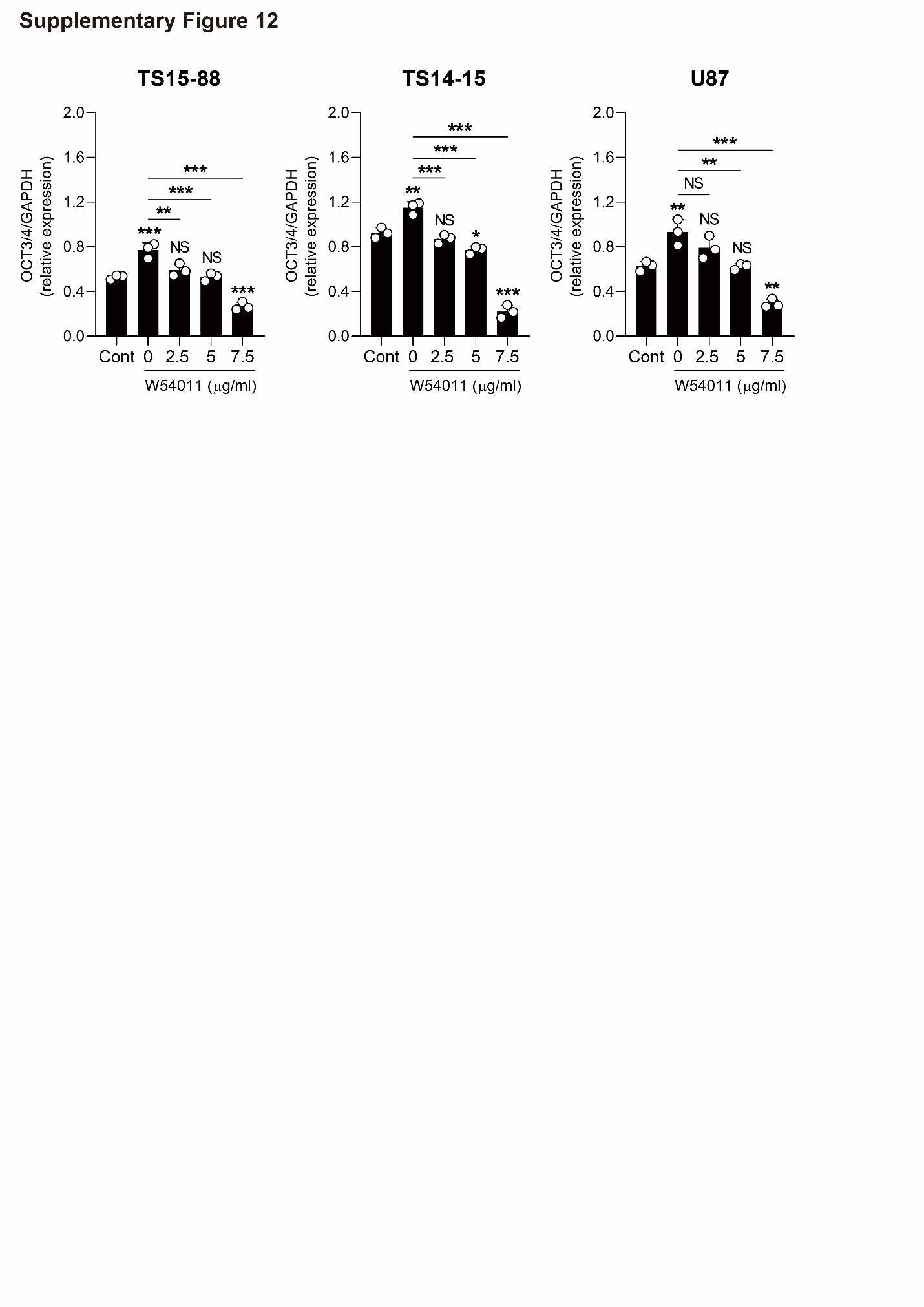
**

**Supplementary Fig. S11.** Raw western blot images corresponding to **Fig. 3c**. Patient-derived tumorspheres (TS15-88, TS14-15) and U87 cells were treated with tMSLC-derived conditioned medium (CM) in the presence of increasing concentrations of W54011 (0, 2.5, 5, and 7.5 µg/mL). Densitometric analyses of these blots are presented as bar graphs. Statistical significance was determined by one-way ANOVA followed by Tukey’s post hoc test. Data are shown as mean ± s.d., with significance denoted as P < 0.05, P < 0.01, and P < 0.001; NS, not significant.

Blots were sectioned prior to hybridisation to allow sequential probing for multiple targets; accordingly, full-length membranes are not available. Bands marked with an asterisk (*) represent replicate blots performed during the revision process using long-term stored samples.

**
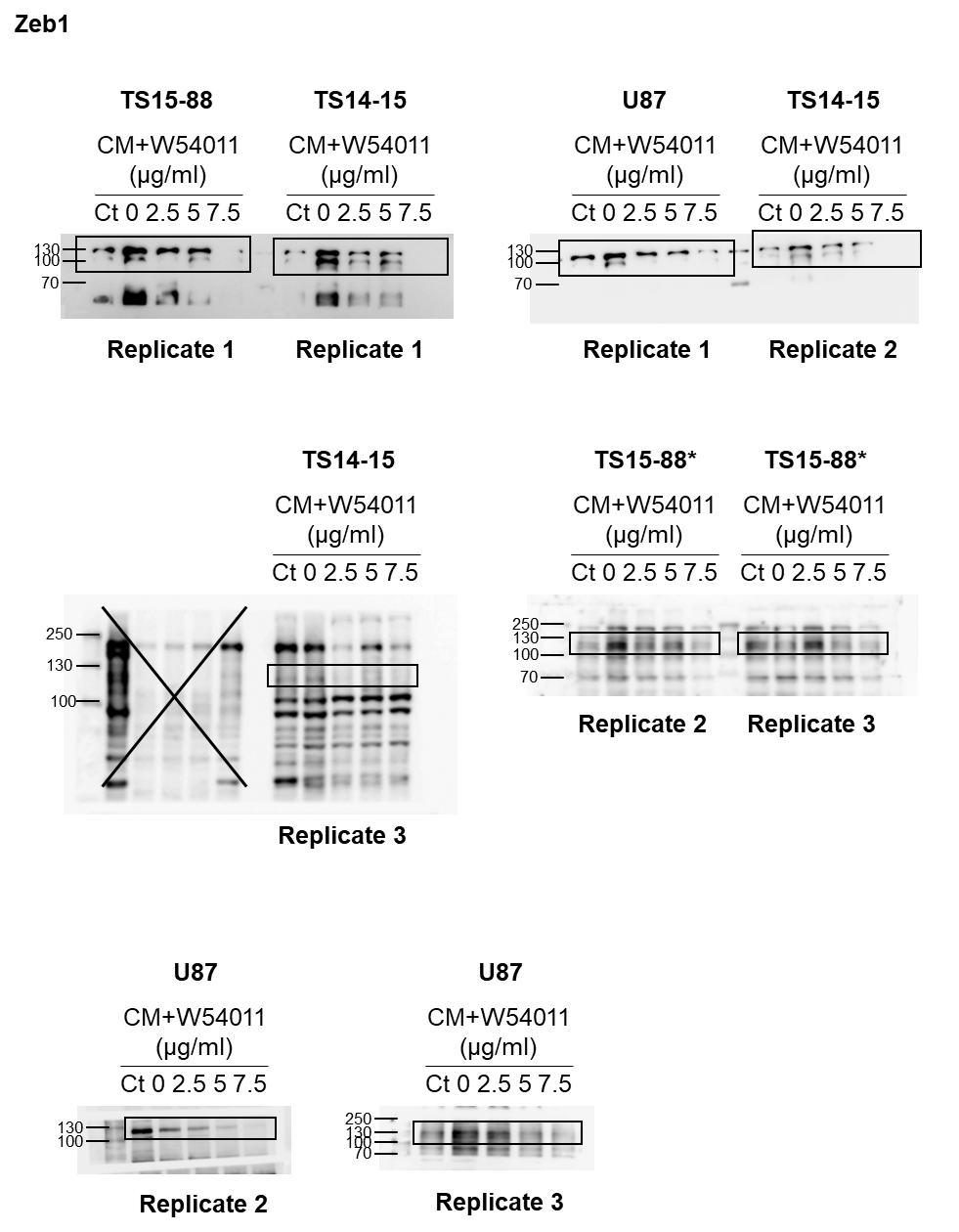
**

**
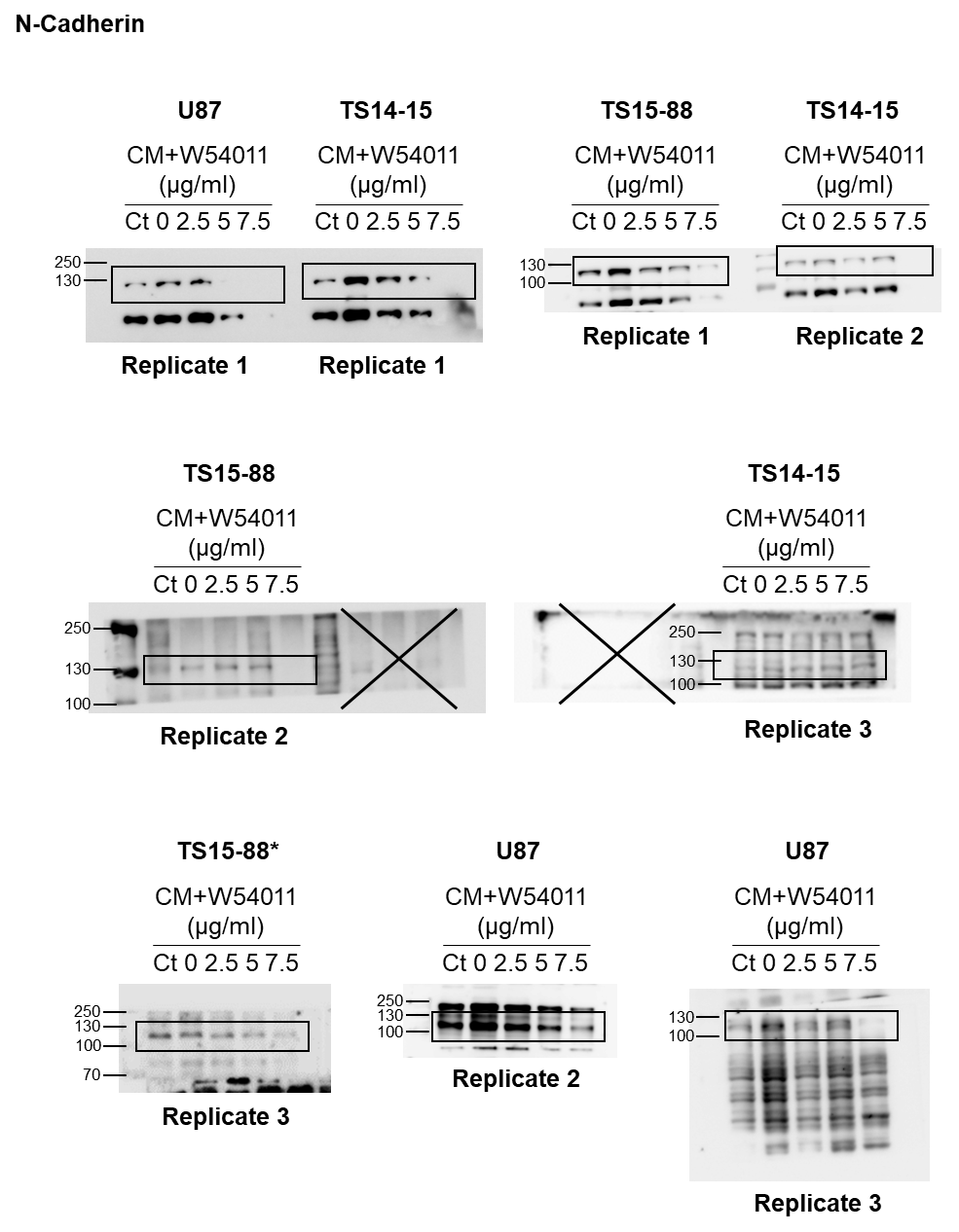
**

**
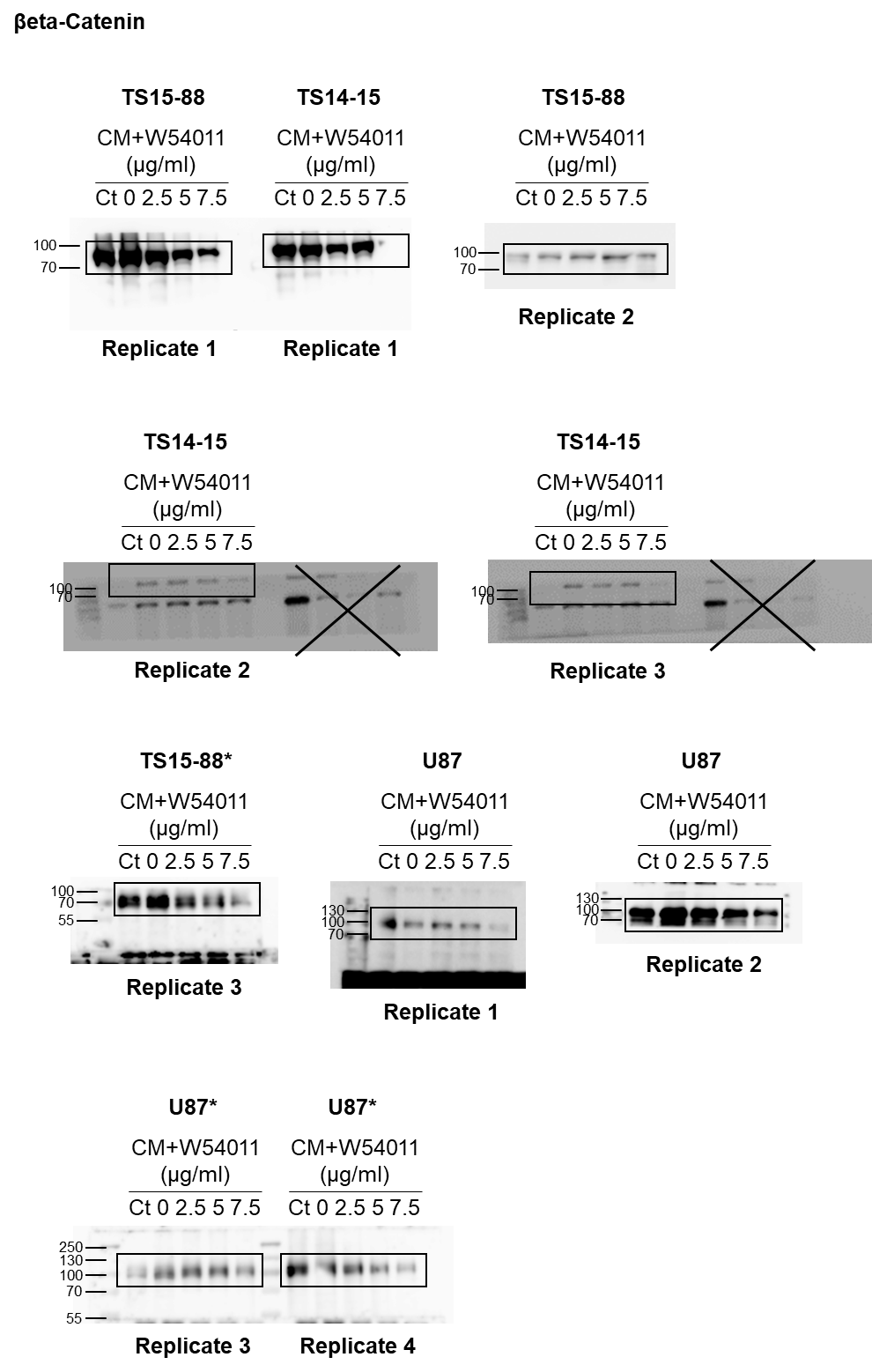
**

**
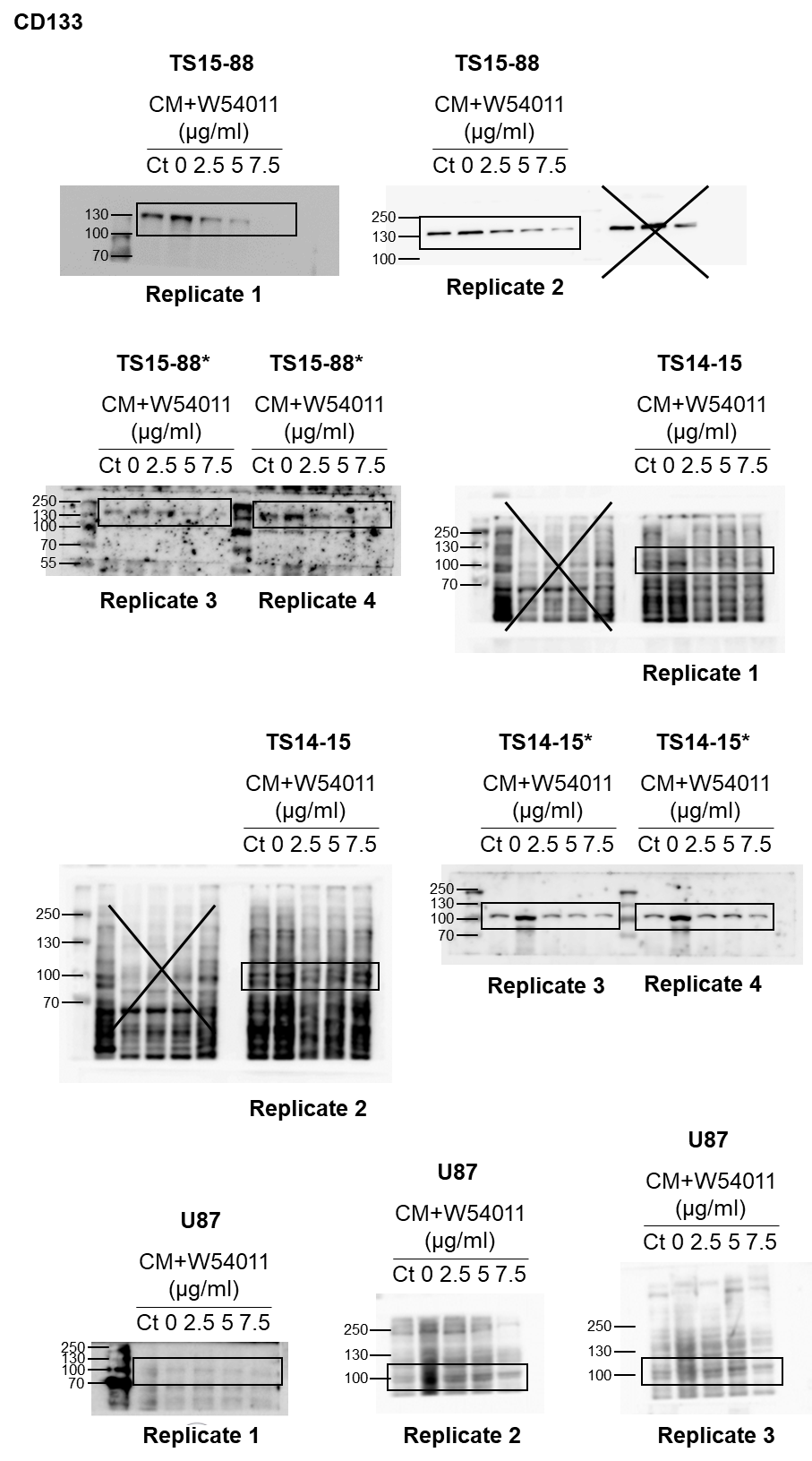

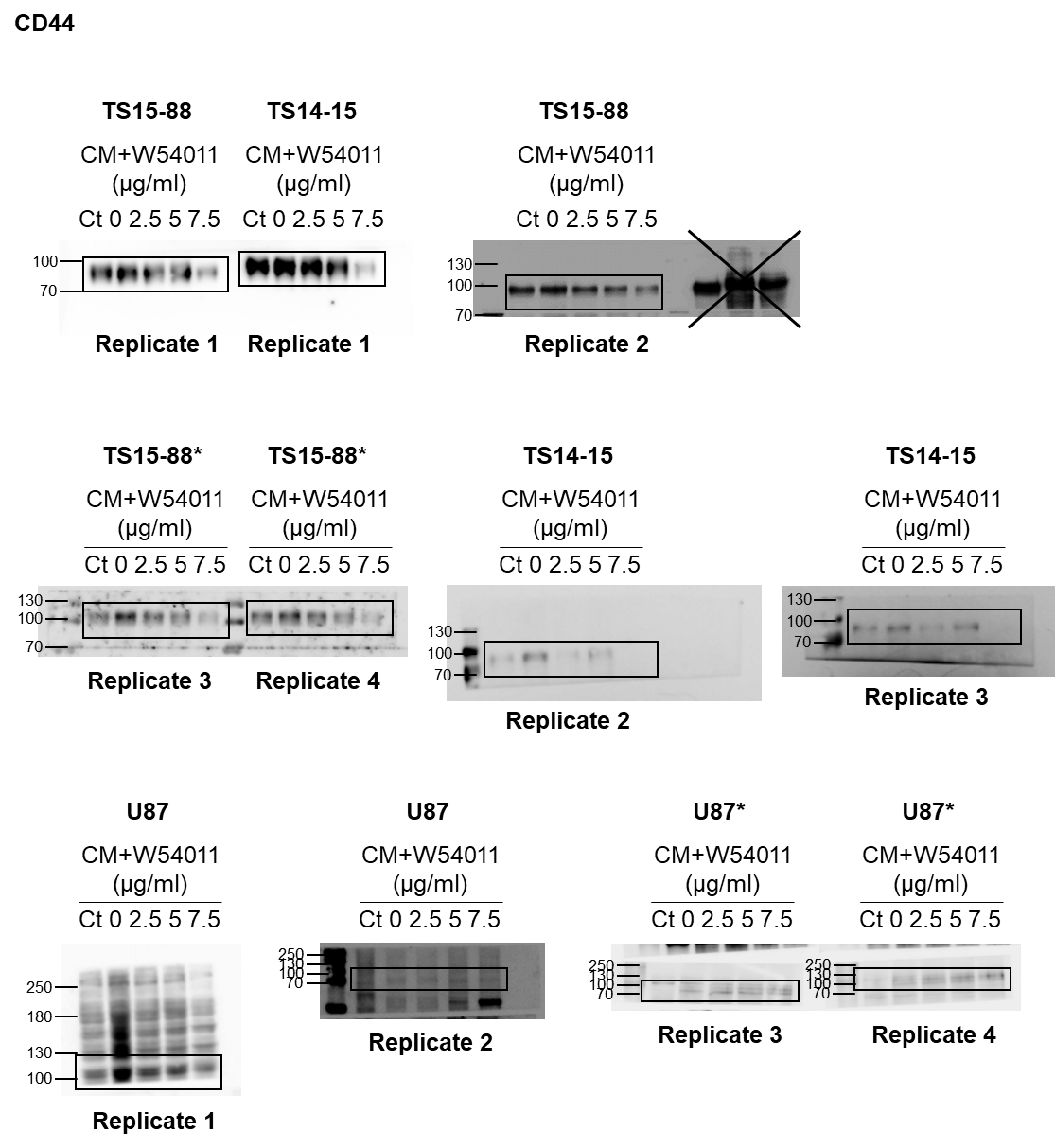
**

**
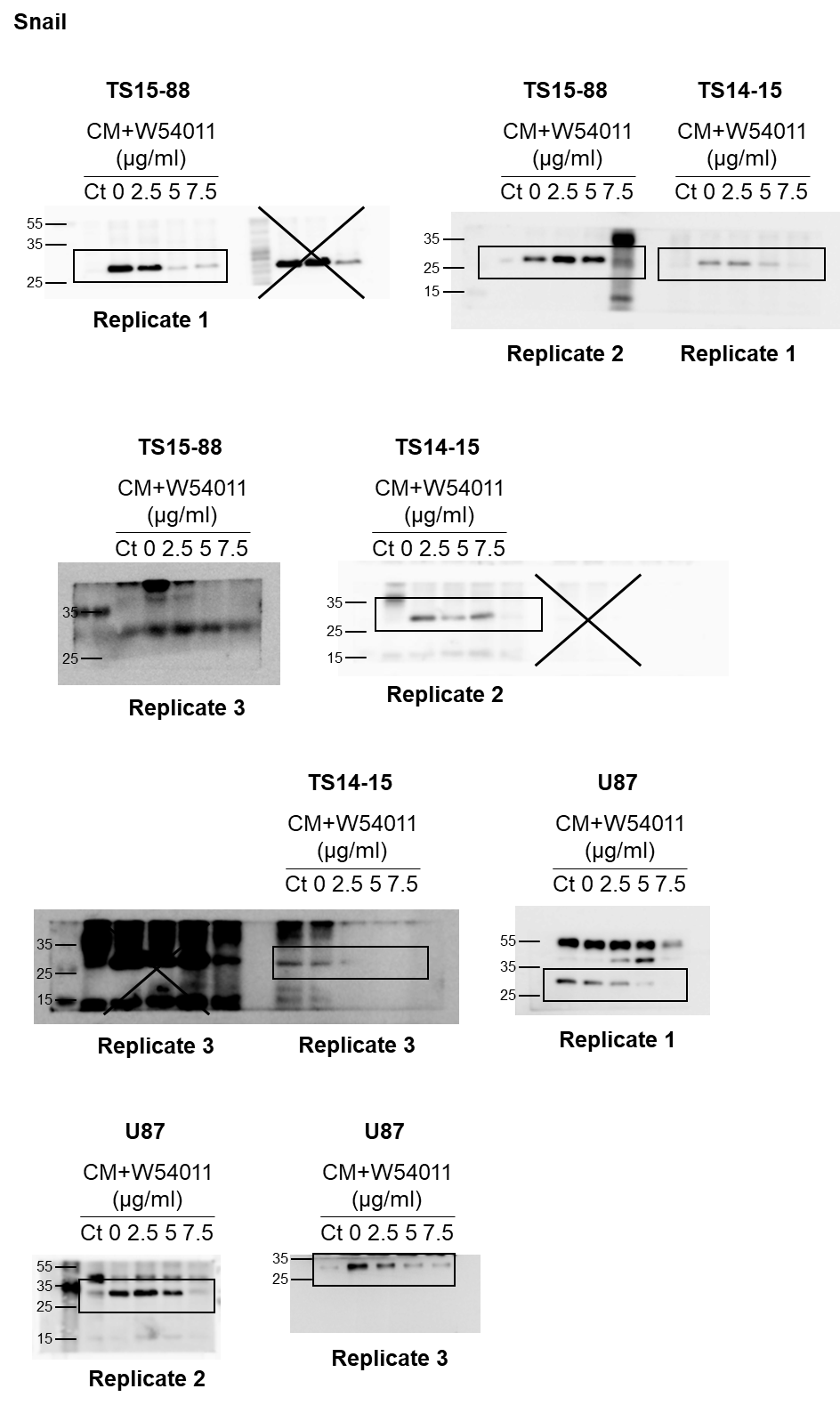

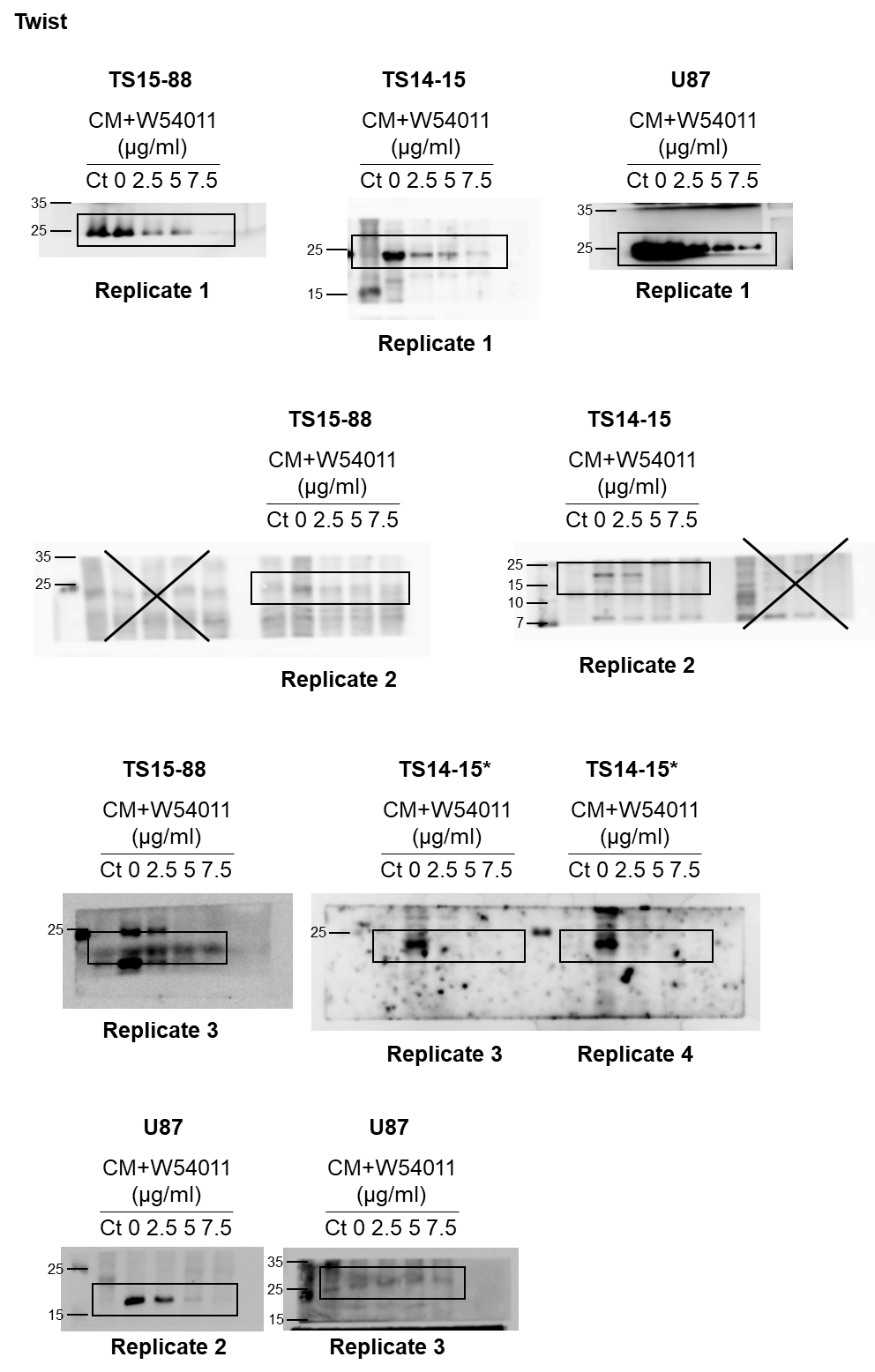

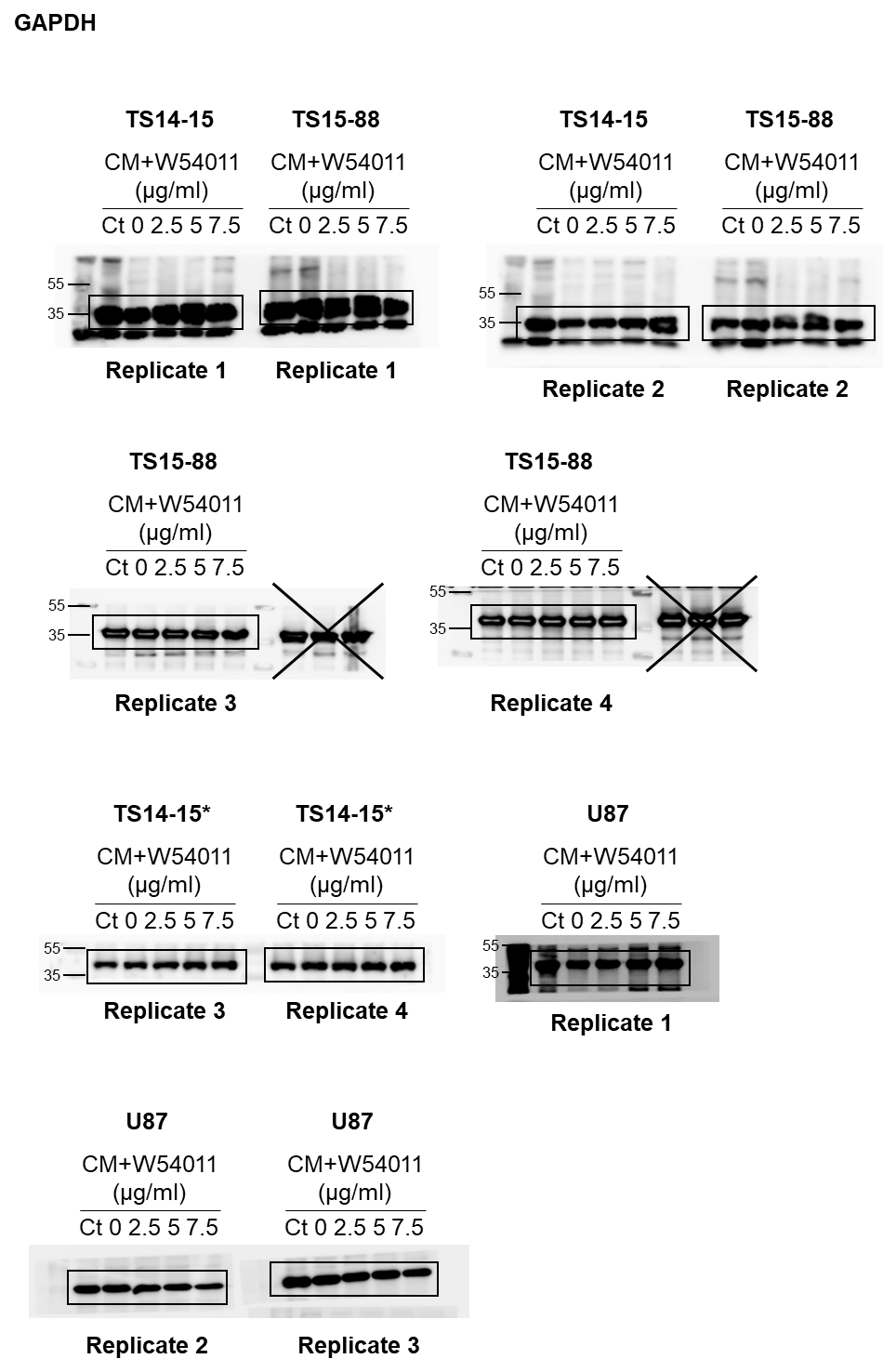

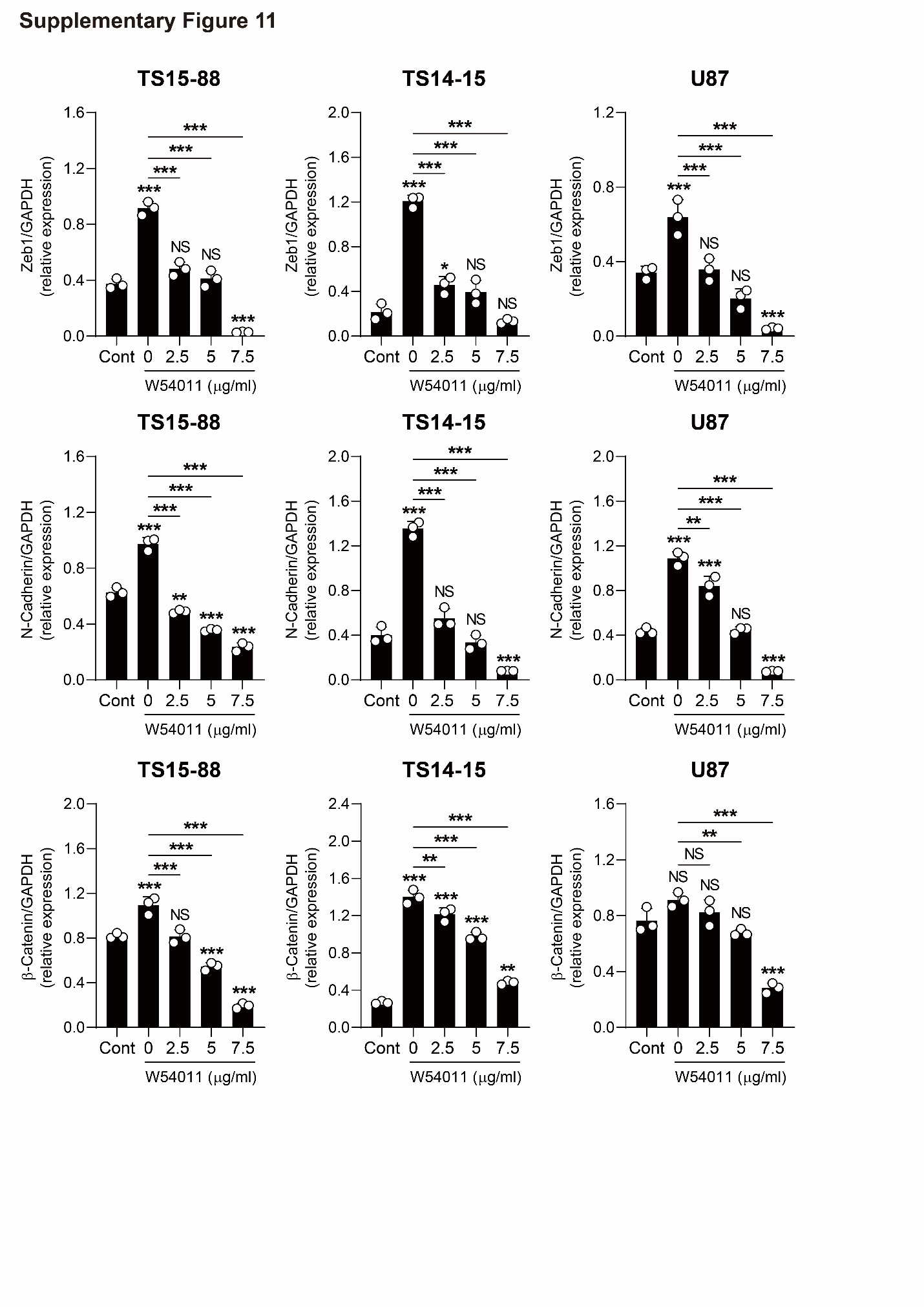
**

**
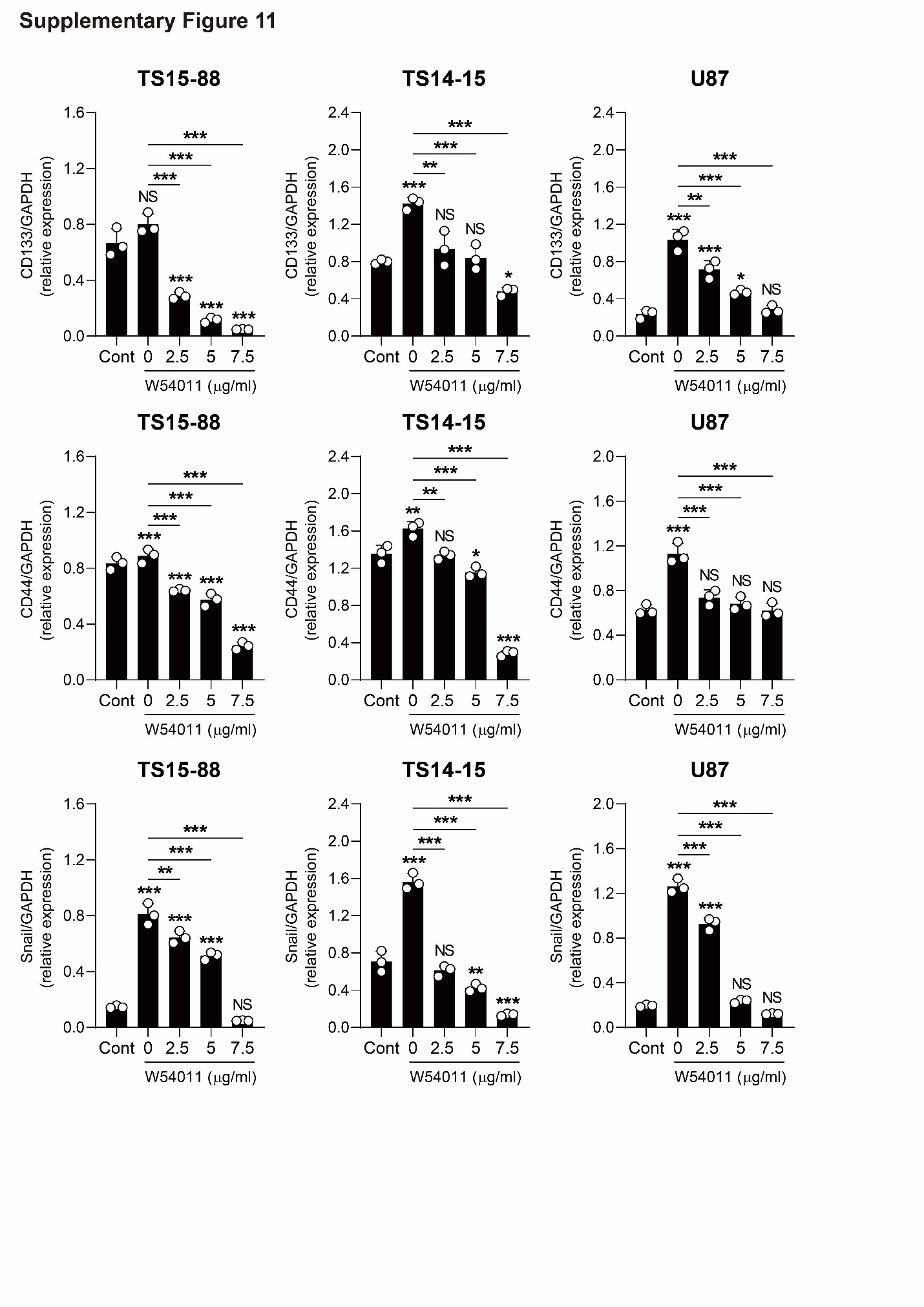
**

**
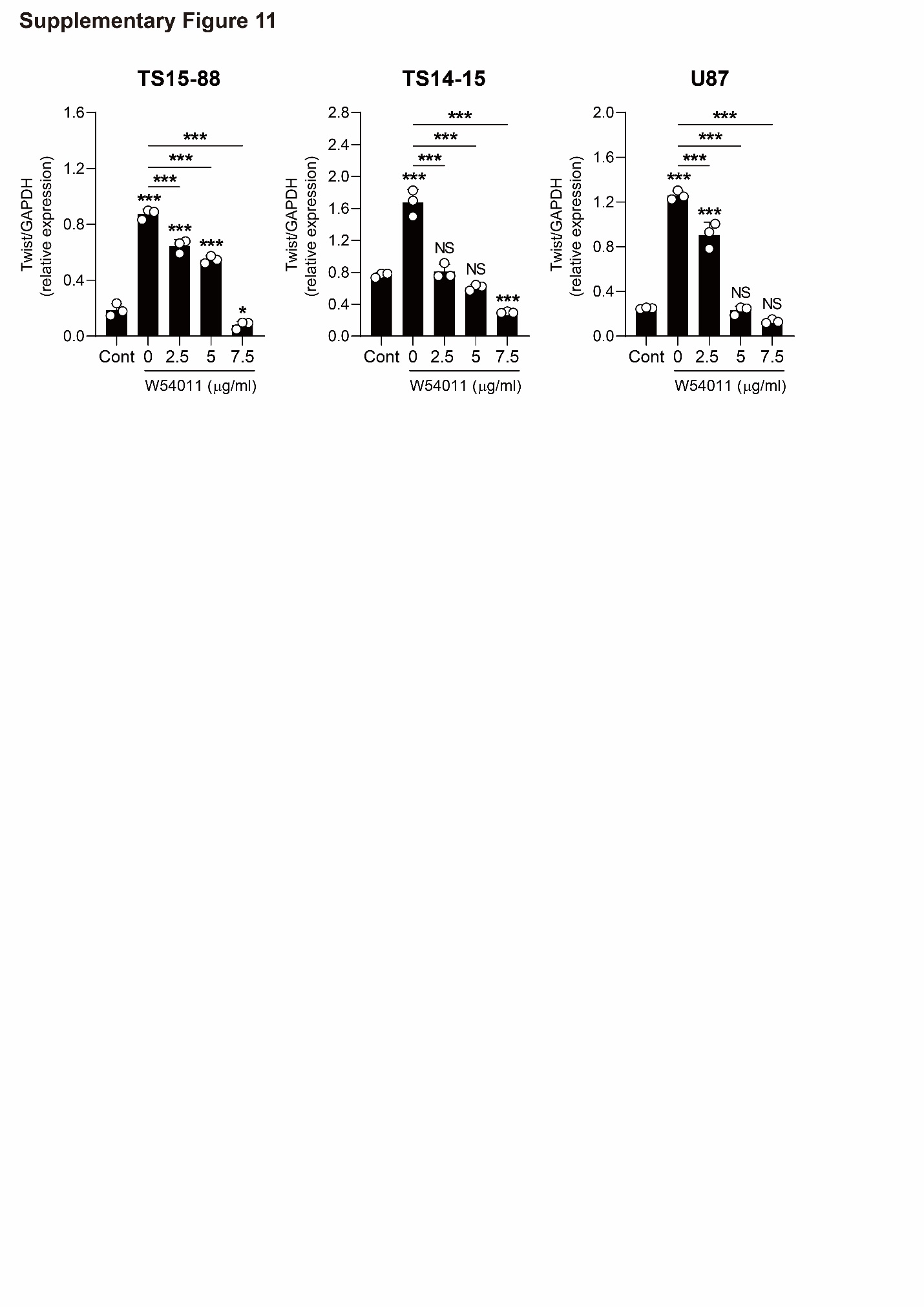
**

**Supplementary Fig. S12.** Raw western blot images corresponding to **Fig. 4c**. Patient-derived tumorspheres (TS15-88, TS14-15) and U87 cells were treated with tMSLC-derived conditioned medium (CM) in the presence of increasing concentrations of W54011 (0, 2.5, 5, and 7.5 µg/mL). Densitometric analyses of these blots are presented as bar graphs. Statistical significance was determined by one-way ANOVA followed by Tukey’s post hoc test. Data are shown as mean ± s.d., with significance denoted as P < 0.05, P < 0.01, and P < 0.001; NS, not significant.

Blots were sectioned prior to hybridisation to allow sequential probing for multiple targets; accordingly, full-length membranes are not available. Bands marked with an asterisk (*) represent replicate blots performed during the revision process using long-term stored samples.

**References**

1. Park, J. *et al.* C5α secreted by tumor mesenchymal stem-like cells mediates resistance to 5-aminolevulinic acid-based photodynamic therapy against glioblastoma tumorspheres. *J Cancer Res Clin* **149**, 4391-4402 (2023).

2. Lim, E. J. *et al.* Crosstalk between GBM cells and mesenchymal stemlike cells promotes the invasiveness of GBM through the C5a/p38/ZEB1 axis. *Neuro-Oncology* **22**, 1452-1462 (2020).

3. Park, J. *et al.* Mesenchymal Stem-Like Cells Derived from the Ventricle More Effectively Enhance Invasiveness of Glioblastoma Than Those Derived from the Tumor. *Yonsei Med J* **64**, 157-166 (2023).

4. Kong, S. H. *et al.* Influence of the Amount of Fresh Specimen on the Isolation of Tumor Mesenchymal Stem-Like Cells from High-Grade Glioma. *Yonsei Med J* **62**, 936-942 (2021).

5. Singh, S. K. *et al.* Identification of a cancer stem cell in human brain tumors. *Cancer Res* **63**, 5821-5828 (2003).

6. Singh, S. K. *et al.* Identification of human brain tumour initiating cells. *Nature* **432**, 396-401 (2004).

7. Robertson, N. *et al.* Structure of the complement C5a receptor bound to the extra-helical antagonist NDT9513727. *Nature* **553**, 111-+ (2018).
